# Supplementary material for: Survival and clinicopathological characteristics of different histological grades of oral cavity squamous cell carcinoma: A single-center retrospective study
Source: PLoS One. 2020 Aug 25;15(8):e0238103. doi: 10.1371/journal.pone.0238103 (PMC7447052; doi:10.1371/journal.pone.0238103)
Supplement: S1 File — (PDF) [file pone.0238103.s001.pdf]

Female0Male1	Survival time (month)	Survival condition(dath"0")	Age at OSCC diagnosed	tumor site	Smoking	Betel	Alcohol	Stage	T stage	N stage	ECS	CM	Recurrence	Recur"1"	ade of tumor	Npositive1
0	17.64	0	53	ant tongue	0	0	0	1	1	1	0	0	2009/10/12	1	2	1
0	25.08	0	51	ant tongue	0	0	0	1	1	x	x	0	0000/00/00	0	2	
0	118.56	1	70	ant tongue	0	0	0	1	1	0	0	0	0000/00/00	0	2	0
0	48.13	0	85	lip	1	1	0	2	2	0	0	0	0000/00/00	0	2	0
0	112.39	1	28	ant tongue	0	0	0	4	2	2	1	0	0000/00/00	0	2	1
0	51.18	0	41	alveolar ridge	0	0	0	4	4	1	0	0	0000/00/00	0	1	1
0	13.28	0	84	alveolar ridge	0	0	0	2	2	0	0	0	0000/00/00	0	2	0
0	106.72	1	78	ant tongue	0	0	0	1	1	0	0	0	0000/00/00	0	1	0
0	53.87	0	59	buccal muco	1	0	0	3	2	1	0	0	2011/1/31	1	3	1
0	106.10	1	74	lip	0	0	0	1	1	x	x	0	0000/00/00	0	2	
0	70.75	0	56	ant tongue	0	0	0	2	2	x	x	0	0000/00/00	0	2	
0	84.30	0	83	buccal muco	0	0	0	3	3	0	0	0	0000/00/00	0	2	0
0	21.34	0	52	ant tongue	0	0	0	1	1	0	0	0	2011/5/24	1	2	0
0	117.44	1	54	buccal muco	0	0	0	1	1	0	0	0	0000/00/00	0	1	0
0	16.23	0	68	alveolar ridge	0	0	0	2	2	0	0	0	2011/2/18	1	2	0
0	103.51	1	79	lip	0	0	0	1	1	x	x	1	0000/00/00	0	2	
0	101.70	1	59	ant tongue	1	0	1	2	2	0	0	0	0000/00/00	0	2	0
0	100.98	1	44	rmt	0	0	0	4	4	x	x	1	0000/00/00	0	3	
0	100.98	1	56	ant tongue	0	0	0	1	1	0	0	0	0000/00/00	0	2	0
0	101.31	1	37	ant tongue	0	0	0	2	2	0	0	0	2011/7/22	1	2	0
0	100.00	1	63	ant tongue	0	0	0	1	1	0	0	0	2015/6/12	1	2	0
0	55.41	0	51	ant tongue	0	0	0	1	1	0	1	0	0000/00/00	0	2	0
0	98.79	1	70	lip	0	0	0	1	1	x	x	0	0000/00/00	0	2	
0	98.92	1	38	ant tongue	0	0	0	1	1	x	x	0	0000/00/00	0	2	
0	98.82	1	49	lip	1	0	0	1	1	0	0	0	0000/00/00	0	2	0
0	20.85	0	70	ant tongue	0	0	0	1	1	x	x	0	2011/6/27	1	2	
0	95.41	1	50	rmt	1	0	0	4	4	0	0	0	0000/00/00	0	2	0
0	11.54	0	62	buccal muco	0	0	0	4	3	2	0	0	2012/2/7	1	2	1
0	29.41	0	71	ant tongue	0	0	0	1	1	0	0	0	0000/00/00	0	1	0
0	40.72	0	42	buccal muco	0	0	0	1	1	x	x	0	2012/3/22	1	1	
0	96.30	1	36	ant tongue	1	0	0	4	4	1	0	0	0000/00/00	0	2	1
0	10.75	0	64	ant tongue	0	0	0	1	1	x	x	0	2011/10/14	1	2	
0	94.36	1	79	buccal muco	1	0	0	1	1	x	x	0	0000/00/00	0	1	
0	91.38	1	35	ant tongue	0	0	0	1	1	0	0	1	0000/00/00	0	3	0
0	91.84	1	79	ant tongue	0	0	0	2	2	0	0	0	2012/6/11	1	2	0
0	90.69	1	69	ant tongue	0	0	0	2	2	0	0	0	0000/00/00	0	2	0
0	8.30	0	81	ant tongue	0	0	0	1	1	x	x	0	2012/2/17	1	2	
0	28.49	0	48	ant tongue	0	0	0	2	2	x	x	0	13-Aug	1	2	
0	89.51	1	52	alveolar ridge	0	0	0	1	1	0	0	0	0000/00/00	0	2	0
0	28.79	0	75	ant tongue	0	0	0	1	1	0	0	0	0000/00/00	0	2	0
0	89.28	1	63	ant tongue	0	0	0	2	2	0	0	0	0000/00/00	0	2	0
0	88.52	1	73	alveolar ridge	1	1	0	4	4	0	0	0	0000/00/00	0	2	0
0	93.54	1	79	alveolar ridge	1	0	0	1	1	x	x	0	0000/00/00	0	1	
0	85.67	1	75	buccal muco	0	0	0	1	1	0	0	0	0000/00/00	0	2	0
0	14.56	0	59	buccal muco	0	0	0	4	4	2	1	0	2012/12/3	1	2	1
0	21.74	0	83	ant tongue	0	0	0	2	2	0	0	0	2013/9/2	1	2	0
0	97.90	1	49	alveolar ridge	1	0	0	1	1	0	0	0	0000/00/00	0	2	0
0	82.56	1	46	ant tongue	0	0	0	3	1	1	0	0	0000/00/00	0	2	1
0	7.44	0	77	ant tongue	0	0	0	2	2	x	x	0	2012/11/29	1	2	
0	34.56	0	53	buccal muco	0	0	0	3	1	1	1	0	0000/00/00	0	2	1
0	78.56	1	61	ant tongue	0	0	0	1	1	x	x	0	0000/00/00	0	1	
0	41.90	0	35	alveolar ridge	0	0	0	4	4	0	0	0	2015/5/15	1	3	0
0	73.61	1	78	alveolar ridge	0	0	0	2	2	0	0	0	0000/00/00	0	2	0
0	61.77	0	85	buccal muco	0	0	0	2	2	0	0	0	0000/00/00	0	2	0
0	34.13	0	73	buccal muco	0	0	0	3	2	1	1	0	0000/00/00	0	2	1
0	8.30	0	50	buccal muco	0	0	0	4	4	x	x	0	0000/00/00	0	2	
0	68.20	1	56	ant tongue	0	0	0	1	1	0	0	0	0000/00/00	0	2	0
0	11.54	0	65	alveolar ridge	1	0	0	4	4	2	1	0	2014/11/10	1	2	1
0	59.80	1	53	ant tongue	0	0	0	1	1	x	x	0	2014/10/7	1	2	
0	59.48	1	46	ant tongue	0	0	0	1	1	0	0	0	0000/00/00	0	2	0
0	59.38	1	49	ant tongue	0	0	0	1	1	0	0	1	0000/00/00	0	2	0
0	7.93	0	51	ant tongue	0	0	1	4	4	0	0	0	2014/12/10	1	2	0
0	58.33	1	59	buccal muco	0	0	0	1	1	x	x	0	0000/00/00	0	1	
0	56.39	1	55	ant tongue	0	0	0	1	1	0	0	0	2017/8/4	1	1	0
0	56.26	1	31	ant tongue	0	0	0	1	1	x	x	0	0000/00/00	0	2	
0	19.08	0	66	buccal muco	0	0	0	4	4	1	1	1	2015/9/4	1	2	1
0	54.20	1	56	ant tongue	0	0	0	1	1	x	x	0	0000/00/00	0	2	
0	32.07	0	85	lip	1	1	0	1	1	0	0	0	2016/12/8	1	2	0
0	54.03	1	74	alveolar ridge	0	0	1	1	1	0	0	0	0000/00/00	0	2	0
0	21.31	0	87	ant tongue	0	0	0	4	1	2	1	0	0000/00/00	0	2	1
0	8.33	0	55	buccal muco	0	0	0	1	1	x	x	0	0000/00/00	0	3	

0	51.11	1	75	ant tongue	0	0	0	4	4	1	0	0	0000/00/00	0	2	1
0	50.85	1	75	ant tongue	0	0	0	1	1	0	0	0	0000/00/00	0	2	0
0	32.66	0	63	ant tongue	0	0	0	1	1 x	x		0	0000/00/00	0	2	
0	48.98	1	80	lip	0	0	0	2	2	0	0	0	0000/00/00	0	2	0
0	16.49	0	52	mouth floor	0	0	0	1	1	0	0	0	0000/00/00	0	2	0
0	45.97	1	69	ant tongue	0	0	0	1	1	0	0	0	0000/00/00	0	2	0
0	43.77	1	53	ant tongue	0	0	0	1	1	0	0	0	0000/00/00	0	2	0
0	43.02	1	58	ant tongue	0	0	0	1	1 x	x		0	0000/00/00	0	2	
0	42.79	1	74	alveolar ridg	0	0	0	4	4	0	0	1	0000/00/00	0	2	0
0	42.52	1	60	ant tongue	1	0	0	3	2	1	0	0	0000/00/00	0	2	1
0	10.59	0	38	ant tongue	1	1	1	4	4	2	1	0	2016/12/8	1	1	1
0	36.75	1	63	buccal muco	1	1	0	4	1	2	1	0	0000/00/00	0	2	1
0	34.46	1	36	alveolar ridg	0	0	0	4	4	0	0	0	0000/00/00	0	2	0
0	5.08	0	82	ant tongue	0	0	0	4	4	1	0	0	2016/11/18	1	2	1
0	31.70	1	73	buccal muco	0	0	0	1	1	0	0	0	0000/00/00	0	2	0
0	7.67	0	72	ant tongue	0	0	0	2	2	0	0	0	2017/5/3	1	2	0
0	30.43	1	64	ant tongue	1	1	0	1	1	0	0	0	0000/00/00	0	2	0
0	2.30	0	19	ant tongue	0	0	0	4	4	0	0	0	0000/00/00	0	3	0
0	26.89	1	63	ant tongue	0	0	0	1	1	0	0	0	0000/00/00	0	2	0
0	26.79	1	55	buccal muco	1	1	1	1	1	0	0	0	0000/00/00	0	2	0
0	1.87	0	33	ant tongue	0	0	0	4	4	2	1	0	0000/00/00	0	2	1
0	26.20	1	72	lip	0	0	0	1	1 x	x		0	0000/00/00	0	2	
0	26.03	1	71	buccal muco	1	0	0	1	1	0	0	0	2017/10/11	1	2	0
0	25.74	1	73	alveolar ridg	0	0	0	1	1 x	x		0	0000/00/00	0	2	
0	25.34	1	37	ant tongue	0	0	0	2	2 x	x		0	2018/4/10	1	2	
0	24.89	1	72	lip	0	0	0	3	2	1	1	0	0000/00/00	0	2	1
0	25.08	1	50	ant tongue	1	1	0	1	1	0	0	0	0000/00/00	0	1	0
0	44.79	1	83	buccal muco	1	1	0	2	2 x	x		0	0000/00/00	0	2	
0	12.33	0	81	rmt	0	0	0	4	4	0	0	0	2018/3/24	1	2	0
0	10.03	0	74	ant tongue	1	0	0	3	3 x	x		0	0000/00/00	0	2	
0	19.84	1	53	ant tongue	0	0	0	4	2	2	0	0	0000/00/00	0	2	1
0	19.41	1	59	ant tongue	0	0	0	1	1 x	x		0	0000/00/00	0	2	
0	18.26	1	71	ant tongue	1	1	1	1	1	0	0	0	0000/00/00	0	3	0
0	17.77	1	71	hard palate	0	0	0	4	2	2	0	1	0000/00/00	0	3	1
0	16.75	1	54	ant tongue	0	0	0	2	2	0	0	0	0000/00/00	0	3	0
0	31.38	1	73	lip	0	0	0	1	1	0	0	0	0000/00/00	0	2	0
0	16.16	1	54	ant tongue	0	0	0	1	1	0	0	0	0000/00/00	0	2	0
0	1.97	0	81	alveolar ridg	0	0	0	4	4	0	0	0	0000/00/00	0	2	0
0	15.05	1	72	rmt	1	0	0	4	4	0	0	0	0000/00/00	0	2	0
0	3.54	0	90	alveolar ridg	0	0	0	4	4	0	0	0	0000/00/00	0	2	0
0	14.46	1	56	buccal muco	0	0	0	1	1 x	x		0	0000/00/00	0	2	
0	9.51	1	75	ant tongue	1	0	0	3	3	0	0	0	0000/00/00	0	2	0
0	9.31	1	56	ant tongue	1	0	0	4	4	3	1	0	2019/5/15	1	2	1
0	9.28	1	51	alveolar ridg	0	0	0	4	4	1	0	0	0000/00/00	0	2	1
0	9.02	1	78	ant tongue	0	1	0	4	3	2	0	0	0000/00/00	0	2	1
0	8.82	1	65	alveolar ridg	0	0	0	4	4	3	0	0	0000/00/00	0	2	1
0	5.41	1	52	buccal muco	0	0	0	1	1 x	x		0	0000/00/00	0	2	
0	5.34	1	57	ant tongue	0	0	0	1	1	0	0	0	0000/00/00	0	2	0
1	17.54	0	44	buccal mucosa				4	4	2	1	0	0000/00/00	0	3	1
1	99.44	0	59	ant tongue				2	2	0	0	0	0000/00/00	0	2	0
1	135.31	1	38	rmt				1	1	0	0	0	2012/6/7	1	2	0
1	18.36	0	59	rmt				4	4	0	0	0	2009/5/15	1	2	0
1	118.85	0	46	ant tongue	1	1	1	1	1	0	0	0	2010/11/26	1	2	0
1	47.18	0	49	buccal mucosa				1	1	0	0	0	2008/10/1	1	2	0
1	52.10	0	51	ant tongue				4	4	2	1	0	0000/00/00	0	2	1
1	55.38	0	65	ant tongue				4	4	0	0	0	0000/00/00	0	1	0
1	18.59	0	39	buccal mucosa				4	4	2	1	0	0000/00/00	0	2	1
1	20.98	0	35	ant tongue				3	2	1	1	0	2009/4/21	1	3	1
1	116.98	0	59	alveolar ridge				4	4	2	0	0	2010/12/25	1	2	1
1	71.02	0	39	rmt				1	1	0	0	0	2013/1/21	1	2	0
1	42.62	0	68	ant tongue				1	1	0	0	0	0000/00/00	0	2	0
1	5.08	0	34	buccal mucosa				4	4	0	1	0	0000/00/00	0	2	0
1	16.10	0	43	ant tongue				4	4	0	0	0	2009/1/14	1	1	0
1	20.13	0	35	buccal mucosa				2	2	0	0	0	0000/00/00	0	2	0
1	51.38	0	61	ant tongue				2	2	0	0	0	0000/00/00	0	2	0
1	122.26	0	60	buccal mucosa				4	4	2	1	0	0000/00/00	0	2	1
1	56.39	0	28	ant tongue				2	2	0	0	0	2010/12/8	1	2	0
1	89.41	0	61	buccal mucosa				4	2	2	0	0	0000/00/00	0	2	1
1	132.26	1	46	rmt				3	1	1	0	0	0000/00/00	0	2	1
1	11.70	0	46	buccal mucosa				4	4	1	1	0	0000/00/00	0	2	1
1	10.52	0	75	ant tongue				4	4	2	1	0	2008/12/26	1	2	1
1	11.31	0	27	buccal mucosa				2	2	0	0	0	2008/11/26	1	2	0

1	37.57	0	73	buccal mucosa				無法		4	x	x		1	0000/00/00	0	1	
1	35.31	0	56	buccal mucosa					4	4		0	0	0	0000/00/00	0	2	0
1	34.33	0	71	alveolar ridge					2	2		0	0	0	0000/00/00	0	3	0
1	42.82	0	42	ant tongue					4	3		2	1	0	0000/00/00	0	2	1
1	38.66	0	67	ant tongue				無法		1	x	x		0	0000/00/00	0	2	
1	132.03	1	72	buccal mucosa					2	2		0	0	1	2012/2/14	1	1	0
1	7.90	0	41	rmt					4	4		0	0	0	2009/1/30	1	2	0
1	20.26	0	49	ant tongue					4	2		2	1	0	2009/2/25	1	2	1
1	58.52	0	56	alveolar ridge					1	1		0	0	0	2010/7/23	1	2	0
1	37.57	0	63	alveolar ridge					4	1		2	0	0	0000/00/00	0	2	1
1	130.49	1	69	lip					1	1		0	0	0	0000/00/00	0	2	0
1	61.61	0	43	alveolar ridge					4	4		0	0	0	0000/00/00	0	2	0
1	126.16	0	49	ant tongue					1	1		0	0	0	0000/00/00	0	2	0
1	118.75	0	50	buccal mucosa					4	4		0	0	0	2012/3/7	1	2	0
1	15.31	0	37	ant tongue				無法		1	x	x		0	0000/00/00	0	2	
1	66.23	0	48	alveolar ridge				無法		4	x	x		0	0000/00/00	0	2	
1	125.77	0	64	ant tongue					3	1		1	0	0	0000/00/00	0	2	1
1	61.02	0	61	ant tongue					1	1		0	0	0	2012/5/29	1	2	0
1	11.25	0	59	alveolar ridge					4	4		2	1	0	2009/2/21	1	2	1
1	54.72	0	53	buccal mucosa				無法		1	x	x		0	2012/10/2	1	1	
1	12.59	0	45	buccal mucosa					4	3		2	1	0	0000/00/00	0	3	1
1	12.10	0	59	mouth floor					1	1		0	0	0	0000/00/00	0	2	0
1	11.44	0	52	hard palate					4	4		1	0	0	0000/00/00	0	2	1
1	15.54	0	41	buccal mucosa				無法		1	x	x		0	2009/5/25	1	2	
1	8.46	0	43	rmt				無法		3	x	x		0	0000/00/00	0	3	
1	20.16	0	35	ant tongue					2	2		0	0	0	2009/9/19	1	2	0
1	40.10	0	59	buccal mucosa					2	2		0	0	0	0000/00/00	0	2	0
1	44.07	0	60	ant tongue					4	4		2	1	0	0000/00/00	0	2	1
1	20.30	0	48	alveolar ridge					4	4		2	1	1	2010/4/7	1	3	1
1	44.23	0	51	buccal mucosa					2	2		0	0	0	0000/00/00	0	2	0
1	13.80	0	51	ant tongue				無法		2	x		0	0	2009/6/27	1	2	
1	53.38	0	46	buccal mucosa					3	3		0	0	0	0000/00/00	0	2	0
1	95.28	0	59	ant tongue					1	1		0	0	0	0000/00/00	0	2	0
1	115.87	0	59	buccal mucosa					4	4		1	1	0	0000/00/00	0	2	1
1	29.61	0	53	alveolar ridge					4	4		2	1	0	0000/00/00	0	2	1
1	128.82	1	36	buccal mucosa					1	1		0	0	0	0000/00/00	0	2	0
1	6.62	0	48	ant tongue					4	4		2	1	1	0000/00/00	0	3	1
1	80.26	0	64	ant tongue					4	4		1	0	1	0000/00/00	0	2	1
1	105.90	0	46	buccal mucosa					1	1		0	0	0	0000/00/00	0	2	0
1	71.70	0	48	lip					2	2		0	0	0	0000/00/00	0	2	0
1	56.92	0	70	buccal mucosa				無法		1	x		0	0	2010/5/3	1	2	
1	23.80	0	46	buccal mucosa					4	3		2	1	0	0000/00/00	0	2	1
1	19.67	0	52	lip				無法		1	x		0	0	2009/7/17	1	2	
1	125.21	1	46	buccal mucosa					4	4		1	1	0	0000/00/00	0	2	1
1	122.62	0	72	buccal mucosa					1	1		0	0	0	0000/00/00	0	1	0
1	65.05	0	36	buccal mucosa					4	4		1	0	0	0000/00/00	0	2	1
1	30.49	0	78	alveolar ridge				無法		2	x	x		0	0000/00/00	0	1	
1	12.10	0	42	ant tongue					4	3		2	1	0	2009/4/3	1	2	1
1	123.61	1	51	ant tongue					1	1		0	0	0	0000/00/00	0	2	0
1	122.89	1	64	buccal muco	0	0	0		4	4		0	0	0	0000/00/00	0	2	0
1	24.62	0	60	ant tongue					2	2		0	0	0	0000/00/00	0	2	0
1	122.66	1	61	ant tongue					3	1		1	0	0	0000/00/00	0	1	1
1	37.11	0	67	rmt					4	4		0	0	0	0000/00/00	0	2	0
1	8.46	0	50	ant tongue					4	4		2	1	0	0000/00/00	0	2	1
1	121.90	1	61	mouth floor					4	4		0	0	0	0000/00/00	0	2	0
1	122.00	1	44	buccal mucosa					4	4		0	0	0	0000/00/00	0	1	0
1	121.74	1	46	buccal mucosa					4	4		0	0	0	0000/00/00	0	1	0
1	60.16	0	76	ant tongue					2	2		0	0	0	0000/00/00	0	2	0
1	33.90	0	75	lip				無法		2	x	x		0	2010/4/19	1	2	
1	22.89	0	62	alveolar ridge					4	4		0	0	0	0000/00/00	0	1	0
1	41.05	0	64	ant tongue					2	2		0	0	0	2012/4/13	1	2	0
1	18.23	0	85	ant tongue					2	2		0	0	0	2010/6/12	1	2	0
1	51.28	0	50	buccal mucosa					1	1		0	0	0	0000/00/00	0	1	0
1	120.43	1	47	buccal mucosa					1	1		0	0	0	0000/00/00	0	1	0
1	40.10	0	59	hard palate					2	2		0	0	0	0000/00/00	0	2	0
1	119.25	1	53	ant tongue					4	4		1	0	0	0000/00/00	0	2	1
1	120.16	1	65	lip					1	1		0	0	0	0000/00/00	0	2	0
1	20.20	0	46	alveolar ridge					4	4		0	0	0	0000/00/00	0	2	0
1	118.75	1	59	ant tongue					4	4		0	0	0	0000/00/00	0	2	0
1	17.02	0	66	ant tongue					2	2		0	0	0	0000/00/00	0	2	0
1	46.82	0	60	buccal mucosa				無法		2	x	x		0	0000/00/00	0	1	

1	58.20	0	69	ant tongue				2	2	0	0	0	0000/00/00	0	2	0
1	118.59	1	45	rmt				4	4	0	0	0	0000/00/00	0	2	0
1	118.43	1	42	lip				3	3	0	0	0	0000/00/00	0	2	0
1	118.72	1	57	buccal mucosa				4	4	0	0	0	0000/00/00	0	2	0
1	117.57	1	43	alveolar ridge				4	4	0	0	0	0000/00/00	0	2	0
1	117.44	1	45	buccal mucosa				4	4	1	0	0	0000/00/00	0	2	1
1	116.72	1	46	buccal mucosa				2	2	0	0	0	0000/00/00	0	2	0
1	15.64	0	53	hard palate				4	4	0	0	0	2010/4/25	1	2	0
1	56.39	0	43	ant tongue				4	4	2	0	0	0000/00/00	0	2	1
1	22.43	0	35	mouth floor				3	1	1	0	0	2010/11/12	1	2	1
1	19.77	0	42	ant tongue				4	4	2	0	0	0000/00/00	0	3	1
1	115.28	1	58	buccal mucosa				4	4	0	0	0	0000/00/00	0	2	0
1	88.33	0	38	ant tongue				3	2	1	0	0	0000/00/00	0	2	1
1	12.39	0	57	lip				1	1	0	0	0	0000/00/00	0	2	0
1	12.82	0	41	rmt				4	4	0	0	0	2010/6/14	1	2	0
1	113.93	1	29	buccal muco	1	1	1	2	2	0	0	0	0000/00/00	0	2	0
1	113.90	1	58	buccal mucosa				3	1	1	0	0	0000/00/00	0	2	1
1	49.02	0	37	buccal mucosa				2	2	0	0	0	2010/11/29	1	1	0
1	40.66	0	56	rmt				2	2	0	0	0	2010/11/3	1	2	0
1	21.11	0	45	buccal mucosa				4	4	2	0	0	2011/8/4	1	2	1
1	13.87	0	56	mouth floor				4	4	2	0	0	0000/00/00	0	1	1
1	113.15	1	52	buccal mucosa				1	1	0	0	1	0000/00/00	0	2	0
1	12.92	0	54	ant tongue				2	2	0	0	0	0000/00/00	0	1	0
1	24.89	0	55	buccal mucosa				2	2	0	0	0	0000/00/00	0	2	0
1	71.70	0	82	buccal mucosa				4	1	2	0	1	0000/00/00	0	2	1
1	112.75	1	50	buccal mucosa				2	2	0	0	0	0000/00/00	0	2	0
1	53.08	0	41	rmt				1	1	0	0	0	0000/00/00	0	2	0
1	112.59	1	49	mouth floor				4	4	1	0	0	0000/00/00	0	2	1
1	112.52	1	58	buccal mucosa				1	1	0	0	0	0000/00/00	0	1	0
1	38.72	0	56	ant tongue				1	1	0	0	0	0000/00/00	0	2	0
1	112.46	1	52	rmt				4	2	2	0	0	0000/00/00	0	2	1
1	28.20	0	49	ant tongue				3	1	1	0	0	2010/12/7	1	2	1
1	112.36	1	55	buccal mucosa				1	1	0	0	1	0000/00/00	0	2	0
1	58.62	0	70	buccal mucosa				4	4	0	0	0	0000/00/00	0	2	0
1	112.13	1	66	alveolar ridge				4	4	0	0	0	0000/00/00	0	2	0
1	49.34	0	63	alveolar ridge				4	4	x	x	0	2011/12/30	1	1	
1	52.43	0	54	rmt				4	4	0	0	0	0000/00/00	0	2	0
1	84.49	0	66	alveolar ridge				1	1	0	0	0	0000/00/00	0	2	0
1	111.90	1	58	rmt				4	4	0	0	0	0000/00/00	0	2	0
1	20.75	0	62	buccal mucosa				2	2	0	0	0	0000/00/00	0	2	0
1	57.31	0	53	mouth floor				2	2	0	0	0	0000/00/00	0	2	0
1	1.84	0	61	ant tongue				4	3	3	1	0	0000/00/00	0	2	1
1	44.20	0	76	ant tongue				2	2	0	0	0	2013/10/26	1	2	0
1	88.10	0	74	rmt				4	4	0	0	0	0000/00/00	0	2	0
1	112.39	1	46	buccal mucosa				3	2	1	0	0	0000/00/00	0	2	1
1	28.62	0	67	buccal mucosa				1	1	0	0	0	0000/00/00	0	2	0
1	111.84	1	56	buccal mucosa				2	2	0	0	0	0000/00/00	0	2	0
1	110.89	1	46	alveolar ridge				4	4	2	0	0	0000/00/00	0	2	1
1	110.92	1	73	ant tongue				2	2	0	0	0	0000/00/00	0	2	0
1	17.77	0	66	ant tongue				4	4	2	0	0	0000/00/00	0	2	1
1	100.89	0	62	lip				2	2	0	0	0	0000/00/00	0	2	0
1	81.02	0	48	mouth floor				1	1	0	0	0	2014/1/12	1	2	0
1	110.82	1	44	buccal mucosa				1	1	x	x	0	0000/00/00	0	1	
1	110.79	1	53	ant tongue				1	1	0	0	0	0000/00/00	0	1	0
1	31.28	0	54	alveolar ridge				1	1	0	0	0	0000/00/00	0	2	0
1	77.08	0	74	alveolar ridge				4	4	0	0	0	0000/00/00	0	2	0
1	19.97	0	84	rmt				4	4	0	0	0	2011/1/31	1	2	0
1	110.62	1	37	ant tongue				1	1	0	0	0	0000/00/00	0	2	0
1	110.72	1	52	buccal mucosa				1	1	0	0	0	0000/00/00	0	2	0
1	110.62	1	49	buccal mucosa				4	1	2	0	0	0000/00/00	0	2	1
1	112.62	1	72	buccal mucosa				3	3	0	0	0	0000/00/00	0	1	0
1	110.43	1	45	buccal mucosa				4	4	2	0	0	0000/00/00	0	2	1
1	110.43	1	47	buccal mucosa				1	1	0	0	0	0000/00/00	0	2	0
1	10.95	0	60	alveolar ridge				4	4	2	0	1	2010/10/9	1	2	1
1	110.66	1	64	buccal mucosa				1	1	0	0	0	0000/00/00	0	2	0
1	39.87	0	57	buccal mucosa				2	2	0	0	0	0000/00/00	0	1	0
1	10.79	0	57	hard palate				4	4	x	x	0	2010/11/20	1	2	
1	55.31	0	51	mouth floor				1	1	0	0	0	2013/11/27	1	2	0
1	110.03	1	71	buccal mucosa				2	2	0	0	0	0000/00/00	0	2	0
1	111.15	1	42	buccal mucosa				1	1	0	0	0	0000/00/00	0	1	0
1	51.05	0	50	hard palate				4	4	x	x	0	0000/00/00	0	2	
1	110.07	1	62	buccal mucosa				1	1	0	0	0	0000/00/00	0	2	0

1	109.57	1	53	lip		4	2		2		0	1	0000/00/00	0	2	1
1	109.57	1	45	buccal mucosa		2	2		0		0	0	2010/12/2	1	2	0
1	110.03	1	57	rmt		1	1		0		0	0	2013/10/29	1	2	0
1	109.51	1	58	lip		1	1		0		0	0	0000/00/00	0	2	0
1	110.33	1	53	lip		4	2		2		0	0	0000/00/00	0	2	1
1	109.34	1	43	buccal mucosa		1	1		0		0	0	0000/00/00	0	2	0
1	109.77	1	40	buccal mucosa		1	1		0		0	0	0000/00/00	0	1	0
1	109.18	1	55	lip		2	2		0		0	0	0000/00/00	0	1	0
1	109.18	1	67	ant tongue		2	2		0		0	0	0000/00/00	0	2	0
1	31.02	0	55	buccal mucosa		3	1		1		0	0	0000/00/00	0	2	1
1	51.28	0	69	lip		1	1	x		x		0	0000/00/00	0	1	
1	110.75	1	42	ant tongue		4	2		2		0	0	0000/00/00	0	2	1
1	109.11	1	62	buccal mucosa		4	4		0		0	0	0000/00/00	0	3	0
1	85.34	0	61	alveolar ridge		4	4		0		0	0	0000/00/00	0	2	0
1	18.85	0	57	rmt		4	1		2		0	0	2010/12/16	1	2	1
1	15.31	0	46	ant tongue		1	1	x		x		0	2011/4/29	1	2	
1	108.66	1	41	ant tongue		3	2		1		0	0	0000/00/00	0	3	1
1	108.59	1	58	ant tongue		1	1		0		0	0	0000/00/00	0	2	0
1	91.80	0	61	hard palate		1	1	x		x		0	2010/12/25	1	2	
1	108.59	1	61	rmt		1	1		0		0	0	2014/2/14	1	2	0
1	108.66	1	42	alveolar ridge		4	4		0		0	0	0000/00/00	0	2	0
1	108.69	1	68	lip		2	2		0		0	0	0000/00/00	0	1	0
1	109.11	1	56	lip		4	4	x		x		0	0000/00/00	0	3	
1	108.46	1	60	buccal mucosa		1	1		0		0	0	2013/9/25	1	2	0
1	109.31	1	64	buccal mucosa		3	1		1		0	0	0000/00/00	0	3	1
1	109.11	1	54	alveolar ridge		2	2		0		0	0	0000/00/00	0	1	0
1	108.26	1	72	buccal mucosa		1	1		0		0	0	0000/00/00	0	2	0
1	108.03	1	53	buccal mucosa		1	1	x		x		0	2011/2/21	1	1	
1	21.38	0	75	buccal mucosa		4	4		2		1	0	0000/00/00	0	2	1
1	108.69	1	67	buccal mucosa	無法		2	x		x		0	2012/5/29	1	2	
1	108.20	1	35	ant tongue		4	2		2		0	0	0000/00/00	0	1	1
1	63.87	0	61	buccal mucosa		2	2		0		0	0	0000/00/00	0	2	0
1	24.46	0	72	mouth floor		1	1		0		0	0	0000/00/00	0	2	0
1	12.33	0	75	rmt		2	2	x		x		0	2010/9/8	1	3	
1	49.15	0	73	buccal mucosa		4	1		2		0	0	0000/00/00	0	2	1
1	35.61	0	57	buccal mucosa		1	1		0		0	0	2012/5/6	1	2	0
1	108.62	1	49	alveolar ridge		1	1		0		0	0	0000/00/00	0	2	0
1	107.93	1	69	buccal mucosa		1	1		0		0	0	2014/8/5	1	1	0
1	107.05	1	52	buccal mucosa		1	1		0		0	0	0000/00/00	0	2	0
1	107.05	1	54	ant tongue		1	1		0		0	0	0000/00/00	0	2	0
1	107.11	1	67	buccal mucosa		2	2	x		x		0	0000/00/00	0	1	
1	106.82	1	46	buccal mucosa		1	1		0		0	0	0000/00/00	0	2	0
1	38.92	0	59	buccal mucosa		1	1	x		x		0	2012/4/21	1	2	
1	109.84	1	51	buccal mucosa		1	1		0		0	0	0000/00/00	0	2	0
1	106.72	1	55	mouth floor		1	1		0		0	0	0000/00/00	0	2	0
1	106.79	1	32	ant tongue		1	1		0		0	0	0000/00/00	0	2	0
1	104.36	0	76	buccal mucosa		1	1	x		x		0	0000/00/00	0	1	
1	106.59	1	60	ant tongue		4	4		0		0	0	0000/00/00	0	2	0
1	109.34	1	48	buccal mucosa		1	1		0		0	0	0000/00/00	0	2	0
1	75.48	0	49	buccal mucosa		1	1		0		0	0	0000/00/00	0	2	0
1	106.52	1	50	alveolar ridge		4	4		1		0	0	0000/00/00	0	2	1
1	106.33	1	46	buccal mucosa		1	1		0		0	0	0000/00/00	0	2	0
1	106.13	1	58	buccal mucosa		1	1		0		0	0	2013/10/2	1	2	0
1	20.30	0	39	buccal mucosa		4	2		2		0	0	2012/3/3	1	2	1
1	106.52	1	61	buccal mucosa		1	1		0		0	0	0000/00/00	0	1	0
1	105.90	1	44	alveolar ridge		2	2		0		0	0	0000/00/00	0	2	0
1	105.57	1	40	alveolar ridge		1	1	x		x		0	0000/00/00	0	2	
1	106.33	1	54	hard palate		4	4		0		0	0	0000/00/00	0	1	0
1	7.41	0	80	alveolar ridge		4	4		2		0	0	0000/00/00	0	2	1
1	105.64	1	56	rmt		1	1	x		x		0	0000/00/00	0	1	
1	70.75	0	44	ant tongue	無法		1	x		x		0	0000/00/00	0	1	
1	15.11	0	58	ant tongue		3	3		0		0	0	0000/00/00	0	2	0
1	78.30	0	59	ant tongue		3	3		1		0	0	0000/00/00	0	2	1
1	105.74	1	52	buccal mucosa		1	1		0		0	0	0000/00/00	0	2	0
1	105.57	0	43	buccal mucosa		4	4		0		0	0	0000/00/00	0	2	0
1	105.44	1	60	alveolar ridge		4	4		0		0	0	0000/00/00	0	1	0
1	104.92	1	57	ant tongue		1	1		0		0	0	0000/00/00	0	2	0
1	104.82	1	69	ant tongue		4	4		1		0	0	0000/00/00	0	2	1
1	50.00	0	61	ant tongue		3	1		1		0	0	0000/00/00	0	2	1
1	104.59	1	46	ant tongue		4	4		0		0	0	0000/00/00	0	2	0
1	39.31	0	82	alveolar ridge		1	1	x		x		0	0000/00/00	0	1	
1	104.43	1	77	alveolar ridge		1	1	x		x		0	0000/00/00	0	2	

1	105.31	1	55	ant tongue				3	3	x	x	0	0000/00/00	0	1	
1	105.51	1	48	buccal mucosa				4	2	2	0	0	0000/00/00	0	2	1
1	105.25	1	39	buccal mucosa				1	1	0	0	0	0000/00/00	0	2	0
1	21.08	0	41	ant tongue				4	4	2	0	0	0000/00/00	0	2	1
1	104.46	1	58	buccal mucosa				1	1	x	x	0	0000/00/00	0	2	
1	104.13	1	53	buccal mucosa				2	2	0	0	0	0000/00/00	0	2	0
1	104.13	1	39	ant tongue				4	4	2	1	0	0000/00/00	0	2	1
1	104.56	1	44	ant tongue				1	1	0	0	0	0000/00/00	0	1	0
1	19.93	0	37	buccal mucosa				1	1	0	0	0	2011/4/16	1	2	0
1	104.00	1	56	lip				1	1	0	0	0	0000/00/00	0	2	0
1	104.00	1	61	buccal mucosa				1	1	0	0	0	0000/00/00	0	1	0
1	104.10	1	71	lip				1	1	x	x	0	0000/00/00	0	2	
1	103.84	1	47	ant tongue				4	2	2	0	0	0000/00/00	0	2	1
1	27.48	0	58	buccal mucosa				1	1	x	x	0	0000/00/00	0	1	
1	23.97	0	49	ant tongue				1	1	x	x	0	2011/12/15	1	2	
1	105.11	1	48	alveolar ridge				1	1	0	0	0	0000/00/00	0	2	0
1	103.67	1	59	ant tongue				4	4	2	0	0	0000/00/00	0	2	1
1	64.82	0	71	ant tongue				2	2	0	0	0	0000/00/00	0	2	0
1	103.44	1	57	buccal mucosa				1	1	x	x	0	2015/2/26	1	2	
1	13.31	0	66	lip				4	1	2	0	0	2011/7/30	1	2	1
1	103.38	1	46	buccal mucosa				2	2	0	0	0	0000/00/00	0	2	0
1	110.75	1	52	buccal mucosa				1	1	x	x	0	0000/00/00	0	1	
1	24.03	0	57	ant tongue				4	4	1	0	0	2012/9/10	1	2	1
1	105.11	0	54	ant tongue				2	2	0	0	0	0000/00/00	0	2	0
1	78.16	0	75	ant tongue				1	1	x	x	0	0000/00/00	0	2	
1	103.28	1	50	buccal mucosa				1	1	0	0	0	0000/00/00	0	2	0
1	103.34	1	66	lip				1	1	0	0	0	0000/00/00	0	2	0
1	104.10	1	61	ant tongue				2	2	0	0	0	0000/00/00	0	2	0
1	63.44	0	66	rmt				4	4	0	0	0	2012/12/12	1	2	0
1	57.77	0	36	buccal mucosa				1	1	0	0	0	0000/00/00	0	2	0
1	103.31	1	41	alveolar ridge				4	4	0	0	0	0000/00/00	0	2	0
1	20.00	0	46	buccal mucosa				4	4	2	0	0	2011/7/4	1	3	1
1	11.21	0	54	ant tongue				3	3	x	x	0	2011/5/10	1	2	
1	66.00	0	70	buccal mucosa				2	2	x	x	0	0000/00/00	0	1	
1	64.16	0	71	lip				3	2	1	0	0	0000/00/00	0	2	1
1	102.85	1	58	rmt				2	2	0	0	0	0000/00/00	0	2	0
1	13.34	0	61	ant tongue				4	2	2	1	0	2011/3/3	1	2	1
1	102.49	1	47	alveolar ridge				4	4	1	0	0	2012/12/28	1	2	1
1	103.31	1	38	buccal mucosa				1	1	0	0	0	0000/00/00	0	2	0
1	17.02	0	47	ant tongue				2	2	0	0	0	2011/5/27	1	2	0
1	102.39	1	53	buccal mucosa				3	2	1	0	0	2012/8/25	1	2	1
1	73.05	0	56	buccal mucosa				2	2	0	0	0	0000/00/00	0	2	0
1	102.49	1	56	ant tongue				1	1	x	x	0	0000/00/00	0	2	
1	11.90	0	58	mouth floor				2	2	x	x	0	0000/00/00	0	2	
1	87.25	0	42	ant tongue				2	2	0	0	0	2011/4/22	1	2	0
1	12.10	0	47	alveolar ridge				4	4	1	0	0	2011/5/2	1	2	1
1	102.39	1	79	rmt				2	2	0	0	0	0000/00/00	0	1	0
1	105.02	1	67	buccal mucosa				3	3	x	x	0	0000/00/00	0	1	
1	101.57	1	68	buccal mucosa				1	1	x	x	0	0000/00/00	0	1	
1	101.38	1	48	lip				1	1	0	0	0	0000/00/00	0	2	0
1	101.21	1	56	buccal mucosa				1	1	0	0	0	0000/00/00	0	2	0
1	59.90	0	49	rmt				2	2	0	0	0	0000/00/00	0	2	0
1	101.02	1	50	buccal mucosa				1	1	0	0	0	0000/00/00	0	2	0
1	100.98	1	55	buccal mucosa				4	4	0	0	0	2011/8/17	1	2	0
1	100.92	1	40	rmt				無法	1	x	x	0	0000/00/00	0	2	
1	45.41	0	72	lip				1	1	0	0	0	0000/00/00	0	2	0
1	14.30	0	58	ant tongue				3	1	1	0	0	2011/7/13	1	2	1
1	50.10	0	57	ant tongue				1	1	0	0	0	0000/00/00	0	2	0
1	100.85	1	54	lip				1	1	0	0	0	0000/00/00	0	2	0
1	100.82	1	56	alveolar ridge				4	4	0	0	0	0000/00/00	0	2	0
1	102.00	1	49	lip				2	2	0	0	0	0000/00/00	0	1	0
1	8.03	0	52	lip	0	0	0	2	2	x	x	0	2011/4/12	1	3	
1	101.15	1	59	buccal mucosa				4	4	0	0	0	0000/00/00	0	2	0
1	89.18	0	57	buccal mucosa				1	1	0	0	0	0000/00/00	0	2	0
1	100.43	1	42	buccal muco	1	1	1	2	2	0	0	0	0000/00/00	0	2	0
1	100.66	1	58	ant tongue	0	0	1	1	1	0	0	0	0000/00/00	0	2	0
1	23.38	0	39	lip	1	1		4	2	2	1	0	0000/00/00	0	2	1
1	100.43	1	56	buccal muco	1	0	0	1	1	x	x	0	0000/00/00	0	1	
1	101.18	1	61	ant tongue				1	1	0	0	0	0000/00/00	0	2	0
1	100.20	1	53	buccal muco	0	0	0	4	4	0	0	0	0000/00/00	0	2	0
1	17.90	0	53	ant tongue				4	4	x	x	0	2011/8/4	1	2	
1	100.13	1	57	ant tongue	1	0	0	1	1	x	x	0	0000/00/00	0	1	

1	83.67	0	72	lip	1	1	0	4	4	0	0	0	0000/00/00	0	2	0
1	100.00	1	51	alveolar ridge	1	1	0	1	1	x	x	0	0000/00/00	0	2	
1	15.28	0	53	buccal muco	1	1		1	1	x	x	0	2011/9/8	1	2	
1	100.10	1	44	buccal muco	1	0	1	4	4	0	0	0	0000/00/00	0	2	0
1	100.43	1	53	lip	0	0	0	1	1	0	0	0	0000/00/00	0	2	0
1	100.62	1	38	ant tongue	1	1	0	1	1	0	0	0	0000/00/00	0	2	0
1	101.34	1	57	buccal mucosa			0	1	1	0	0	0	0000/00/00	0	1	0
1	99.87	1	58	buccal muco	1	1	0	1	1	x	x	0	0000/00/00	0	3	
1	48.10	0	47	mouth floor	0	0	0	2	2	0	0	0	0000/00/00	0	2	0
1	8.49	0	44	alveolar ridge	1	1	1	1	1	x	x	0	0000/00/00	0	2	
1	24.00	0	56	ant tongue				4	4	0	0	0	0000/00/00	0	2	0
1	99.48	1	62	buccal muco	1	0	0	2	2	0	0	0	0000/00/00	0	2	0
1	11.48	0	62	alveolar ridge	0	0	0	4	4	0	0	0	0000/00/00	0	2	0
1	99.51	1	40	ant tongue	1	1	1	2	2	0	0	0	0000/00/00	0	2	0
1	98.07	0	51	buccal muco	1	1	0	2	2	0	0	0	0000/00/00	0	2	0
1	99.34	1	43	buccal muco	1	0	0	1	1	0	0	0	0000/00/00	0	2	0
1	21.15	0	66	ant tongue	0	0	0	4	2	2	1	0	2012/5/17	1	2	1
1	13.70	0	54	ant tongue	1	1		2	2	0	0	0	2011/9/8	1	2	0
1	99.54	1	71	ant tongue	0	0	0	3	1	1	0	0	0000/00/00	0	2	1
1	90.10	0	53	hard palate	1	1	0	2	2	0	0	0	0000/00/00	0	2	0
1	99.18	1	45	ant tongue	0	0	0	1	1	0	0	0	0000/00/00	0	2	0
1	80.13	0	59	buccal muco	1	0	0	1	1	0	0	0	0000/00/00	0	2	0
1	58.39	0	64	ant tongue	0	0	1	2	2	0	0	0	0000/00/00	0	2	0
1	99.02	1	60	buccal muco	1	1	0	1	1	0	0	0	0000/00/00	0	2	0
1	99.21	1	57	lip	1	1	1	1	1	x	x	0	0000/00/00	0	2	
1	100.23	1	57	ant tongue	0	0	0	1	1	0	0	0	0000/00/00	0	1	0
1	99.25	1	56	hard palate	0	0	0	4	4	0	0	0	0000/00/00	0	1	0
1	99.15	1	62	ant tongue	1	0	0	1	1	0	0	0	0000/00/00	0	2	0
1	99.08	1	59	buccal muco	0	0	0	4	4	0	0	0	0000/00/00	0	2	0
1	82.66	0	49	ant tongue	1	1	0	1	1	0	0	0	0000/00/00	0	2	0
1	98.79	1	37	rmt	0	0	0	4	4	x	x	0	0000/00/00	0	2	
1	18.82	0	31	ant tongue	1	0	0	1	1	0	0	0	0000/00/00	0	2	0
1	99.41	1	41	ant tongue	1	0	1	4	4	2	1	0	0000/00/00	0	2	1
1	98.56	1	58	ant tongue	0	0	0	4	4	0	0	0	0000/00/00	0	2	0
1	98.36	1	53	buccal muco	1	1	0	3	1	1	0	0	0000/00/00	0	2	1
1	93.02	0	59	buccal muco	1	0	1	4	4	2	0	0	0000/00/00	0	2	1
1	98.39	1	37	ant tongue	0	0	0	4	4	0	0	0	0000/00/00	0	2	0
1	98.16	1	60	buccal muco	0	0	0	1	1	0	0	0	0000/00/00	0	2	0
1	98.13	1	44	ant tongue	0	0	0	4	4	2	1	0	0000/00/00	0	2	1
1	98.26	1	51	alveolar ridge	0	1	0	1	1	0	0	0	0000/00/00	0	1	0
1	98.10	1	49	buccal muco	1	1	1	4	4	1	0	0	0000/00/00	0	2	1
1	16.36	0	68	lip	0	0	0	4	4	0	0	0	0000/00/00	0	2	0
1	17.41	0	48	buccal muco	1	0	0	4	4	2	1	1	2012/7/11	1	2	1
1	98.72	1	63	rmt	1	1	0	4	4	0	0	0	0000/00/00	0	2	0
1	42.82	0	74	ant tongue	0	0	0	1	1	x	x	0	2012/4/18	1	2	
1	11.08	0	54	buccal muco	1	0	0	4	4	2	0	1	0000/00/00	0	2	1
1	13.18	0	71	buccal muco	1	0	0	4	4	1	0	0	0000/00/00	0	1	1
1	12.59	0	83	buccal muco	0	0	0	4	4	0	0	0	0000/00/00	0	3	0
1	102.62	1	56	buccal mucosa				無法	2	x	x	0	0000/00/00	0	3	
1	98.07	1	48	buccal muco	1	0	0	2	2	0	0	0	0000/00/00	0	2	0
1	2.92	0	77	buccal muco	0	0	0	4	3	2	0	0	0000/00/00	0	2	1
1	97.41	1	51	hard palate	1	0	0	1	1	0	0	0	0000/00/00	0	2	0
1	109.31	1	71	ant tongue				2	2	0	0	0	0000/00/00	0	2	0
1	4.79	0	56	buccal muco	0	0	0	4	4	0	0	0	0000/00/00	0	2	0
1	18.26	0	59	ant tongue	1	1	1	4	4	0	0	0	0000/00/00	0	2	0
1	50.33	0	49	alveolar ridge	1	1	0	4	4	2	0	0	0000/00/00	0	2	1
1	97.41	1	74	buccal muco	0	0	0	2	2	0	0	0	0000/00/00	0	2	0
1	97.70	1	58	ant tongue	1	0	0	2	2	0	0	0	2011/9/1	1	2	0
1	102.00	1	49	buccal mucosa				1	1	0	0	0	0000/00/00	0	1	0
1	19.11	0	37	ant tongue	0	0	0	1	1	0	0	0	2012/2/21	1	2	0
1	57.54	0	48	ant tongue	1	0	0	1	1	0	0	0	0000/00/00	0	2	0
1	92.30	0	70	lip	0	0	0	1	1	0	0	0	0000/00/00	0	2	0
1	101.77	1	37	buccal mucosa				4	4	0	0	0	0000/00/00	0	2	0
1	97.38	1	48	alveolar ridge	0	0	0	4	4	0	0	0	0000/00/00	0	2	0
1	96.98	1	59	buccal muco	1	0	0	2	2	0	0	0	0000/00/00	0	1	0
1	67.05	0	59	alveolar ridge	0	0	0	4	4	x	x	0	2012/10/20	1	2	
1	97.54	1	52	alveolar ridge	1	0	0	2	2	0	0	0	0000/00/00	0	1	0
1	21.93	0	70	ant tongue	0	0	0	1	1	0	0	0	0000/00/00	0	2	0
1	39.74	0	71	alveolar ridge	1	1	0	4	4	0	0	0	0000/00/00	0	2	0
1	97.21	1	54	ant tongue	1	0	1	1	1	0	0	0	0000/00/00	0	2	0
1	97.41	1	56	mouth floor	1	1	1	2	2	0	0	0	0000/00/00	0	3	0
1	96.75	1	44	buccal muco	1	0	0	1	1	0	0	0	0000/00/00	0	2	0

1	96.89	1	43	buccal muco	1	0	0	4	4	0	0	0	0000/00/00	0	2	0
1	96.66	1	53	ant tongue	1	1	0	2	2	0	0	0	0000/00/00	0	2	0
1	8.36	0	51	ant tongue	1	0	0	4	4	2	1	0	2011/11/3	1	2	1
1	96.69	1	57	alveolar ridg	1	0	0	3	2	1	0	0	0000/00/00	0	2	1
1	32.72	0	55	ant tongue	1	1	1	2	2	0	0	0	0000/00/00	0	2	0
1	11.08	0	35	buccal muco	0	0	0	4	4	2	1	0	2011/6/26	1	2	1
1	109.31	1	62	hard palate				1	1	0	0	0	0000/00/00	0	2	0
1	101.77	1	37	lip				2	2	0	0	0	0000/00/00	0	2	0
1	97.93	1	56	alveolar ridg	1	0	0	4	4	2	1	0	0000/00/00	0	2	1
1	15.67	0	47	ant tongue	1	0	0	4	4	2	1	0	2012/3/12	1	2	1
1	96.23	1	37	buccal muco	1	1	0	1	1	0	0	0	0000/00/00	0	2	0
1	96.39	1	47	buccal muco	1	0	0	4	4	0	0	0	2013/3/20	1	2	0
1	29.48	0	56	buccal muco	0	0	0	1	1	x	x	0	2012/5/16	1	2	
1	32.13	0	73	buccal muco	0	0	0	2	2	0	0	0	2013/11/26	1	2	0
1	13.08	0	61	buccal muco	1	0	0	1	1	x	x	0	2012/6/16	1	1	
1	9.80	0	58	ant tongue	1	0	0	4	4	0	0	0	0000/00/00	0	2	0
1	14.26	0	58	alveolar ridg	1	1	0	4	1	2	0	0	2012/4/20	1	2	1
1	96.03	1	77	buccal muco	0	1	0	1	1	x	x	0	0000/00/00	0	1	
1	95.61	1	71	lip	0	0	0	1	1	0	0	0	0000/00/00	0	2	0
1	95.54	1	56	ant tongue	0	0	0	3	3	1	0	1	0000/00/00	0	2	1
1	95.61	1	58	alveolar ridg	1	1	0	4	4	0	0	0	0000/00/00	0	2	0
1	95.38	1	56	ant tongue	1	0	0	2	2	0	0	0	2014/2/19	1	2	0
1	95.41	1	41	buccal muco	1	1	0	1	1	0	0	0	2015/3/30	1	2	0
1	72.46	0	48	hard palate	1	0	0	4	4	1	0	0	0000/00/00	0	2	1
1	7.41	0	57	hard palate	0	0	1	1	1	x	x	0	0000/00/00	0	2	
1	95.18	1	59	ant tongue	0	0	0	1	1	x	x	0	2015/2/6	1	2	
1	21.67	0	60	buccal muco	1	1	0	2	2	0	0	0	0000/00/00	0	2	0
1	42.33	0	76	buccal muco	1	1	0	3	3	0	0	0	0000/00/00	0	1	0
1	95.08	1	41	ant tongue	1	0	0	3	3	0	0	0	0000/00/00	0	2	0
1	95.08	1	52	alveolar ridg	0	0	0	1	1	x	x	0	2012/3/8	1	2	
1	25.41	0	78	lip	0	0	0	1	1	x	x	0	2012/2/24	1	2	
1	63.90	0	71	alveolar ridg	0	0	0	4	4	0	0	0	0000/00/00	0	3	0
1	10.59	0	58	buccal muco	0	0	0	4	4	2	1	0	2012/3/12	1	2	1
1	15.48	0	55	buccal muco	1	1	1	2	2	0	0	0	2011/11/16	1	2	0
1	12.98	0	62	alveolar ridg	0	0	0	4	4	0	0	0	2011/12/2	1	2	0
1	94.85	1	48	alveolar ridg	0	0	0	1	1	x	x	0	0000/00/00	0	2	
1	30.23	0	56	ant tongue	1	1		4	4	2	0	0	0000/00/00	0	2	1
1	62.79	0	63	buccal muco	0	0		1	1	0	0	0	0000/00/00	0	2	0
1	21.25	0	38	buccal muco	1	0	0	3	2	1	0	0	0000/00/00	0	2	1
1	12.66	0	54	rmt	1	1		1	1	0	0	0	2011/9/8	1	2	0
1	19.18	0	49	rmt	0	0	0	4	4	1	0	0	2012/7/19	1	2	1
1	53.28	0	53	buccal muco	1	1	0	3	3	1	1	0	0000/00/00	0	2	1
1	94.89	1	54	lip	0	0		1	1	0	0	0	0000/00/00	0	2	0
1	94.56	1	56	buccal muco	1	1		4	1	2	1	0	0000/00/00	0	2	1
1	10.26	0	38	ant tongue	1	1	1	4	4	2	1	0	0000/00/00	0	2	1
1	38.98	0	52	alveolar ridg	0	0	0	4	4	0	0	0	0000/00/00	0	1	0
1	94.82	1	53	buccal muco	0	1	1	2	2	0	0	0	0000/00/00	0	2	0
1	66.49	0	45	rmt	1	0	0	4	1	2	0	0	2016/2/11	1	2	1
1	104.00	1	61	hard palate				1	1	0	0	0	0000/00/00	0	1	0
1	94.26	1	37	ant tongue	1	0	1	2	2	0	0	0	0000/00/00	0	2	0
1	94.66	1	45	ant tongue	1	0	0	2	2	0	0	0	0000/00/00	0	2	0
1	94.20	1	30	ant tongue	1	0	0	4	4	2	0	0	0000/00/00	0	2	1
1	94.20	1	51	buccal muco	0	0	0	1	1	x	x	0	2014/12/18	1	2	
1	12.89	0	57	buccal muco	0	0		4	2	2	1	0	2012/3/7	1	2	1
1	94.13	1	55	alveolar ridg	1	0	1	4	4	0	0	0	0000/00/00	0	2	0
1	17.70	0	54	buccal muco	1	0	0	2	2	0	0	0	2011/10/20	1	2	0
1	89.41	0	53	lip	0	1	0	1	1	x	x	0	0000/00/00	0	2	
1	94.03	1	72	buccal muco	1	0	0	4	2	2	1	0	0000/00/00	0	2	1
1	94.95	1	52	ant tongue	0	0	0	1	1	0	0	0	0000/00/00	0	2	0
1	9.57	0	49	ant tongue	0	0	1	2	2	0	0	0	0000/00/00	0	2	0
1	28.95	0	67	ant tongue	0	0	0	4	4	2	1	0	2012/11/22	1	2	1
1	47.18	0	73	lip	1	0	0	1	1	0	0	0	2013/12/5	1	1	0
1	16.49	0	68	alveolar ridg	0	0	0	4	4	0	0	0	2012/9/26	1	2	0
1	93.74	1	56	ant tongue	1	0	0	4	4	0	0	0	0000/00/00	0	1	0
1	68.66	0	66	lip	0	0	0	2	2	0	0	0	0000/00/00	0	2	0
1	93.54	1	54	buccal muco	1	0	0	4	2	2	0	0	0000/00/00	0	2	1
1	93.28	1	81	ant tongue	0	0	0	1	1	0	0	0	0000/00/00	0	1	0
1	93.31	1	38	ant tongue	1	1		2	2	0	0	0	0000/00/00	0	2	0
1	93.05	1	57	ant tongue	1	0	0	1	1	0	0	0	0000/00/00	0	2	0
1	93.80	1	52	alveolar ridg	1	1		2	2	0	0	0	0000/00/00	0	2	0
1	11.08	0	58	alveolar ridg	0	0	0	4	4	0	0	0	2012/6/5	1	2	0
1	92.95	1	57	ant tongue	0	0	0	4	4	0	0	0	0000/00/00	0	2	0

1	93.90	1	53	buccal muco	1	1	0	2	2	0	0	0	0000/00/00	0	1	0
1	92.75	1	56	rmt	0	0	0	4	4	0	0	0	0000/00/00	0	2	0
1	15.61	0	47	ant tongue	0	0	0	4	4	2	1	0	2012/5/10	1	2	1
1	18.36	0	56	ant tongue	0	0	0	2	2	x	x	0	2012/8/29	1	2	
1	22.85	0	52	buccal muco	0	0	0	4	4	0	0	0	2012/2/21	1	2	0
1	92.82	1	45	buccal muco	1	1	0	2	2	0	0	0	0000/00/00	0	1	0
1	24.95	0	77	buccal muco	0	0	0	2	2	0	0	0	0000/00/00	0	2	0
1	23.54	0	39	buccal muco	1	1	1	4	4	0	0	0	0000/00/00	0	2	0
1	92.43	1	61	ant tongue	0	0	0	4	4	0	0	0	0000/00/00	0	2	0
1	103.41	1	66	buccal mucosa				1	1	0	0	0	0000/00/00	0	2	0
1	91.97	1	50	buccal muco	1	0	1	4	4	0	0	0	2015/5/12	1	2	0
1	92.07	1	53	alveolar ridge	1	1	0	4	4	2	1	0	0000/00/00	0	2	1
1	91.93	1	44	ant tongue	0	0	0	1	1	0	0	0	0000/00/00	0	2	0
1	13.57	0	41	ant tongue	1	0	0	4	3	2	1	0	2012/4/19	1	2	1
1	91.90	1	50	buccal muco	0	0	0	1	1	0	0	0	0000/00/00	0	2	0
1	40.85	0	77	buccal muco	0	0	0	2	2	0	0	0	0000/00/00	0	2	0
1	92.20	1	55	rmt	1	0	1	4	4	2	1	1	0000/00/00	0	2	1
1	92.79	1	51	buccal muco	0	0	0	4	4	0	0	0	0000/00/00	0	2	0
1	92.39	1	56	ant tongue	1	0	1	2	2	0	0	0	0000/00/00	0	2	0
1	91.57	1	49	alveolar ridge	1	0	1	2	2	0	0	0	0000/00/00	0	2	0
1	91.38	1	57	ant tongue	0	0	0	1	1	0	0	0	0000/00/00	0	2	0
1	91.48	1	37	ant tongue	1	1	1	4	4	1	0	0	0000/00/00	0	2	1
1	84.92	0	56	alveolar ridge	1	1	1	4	4	0	0	0	0000/00/00	0	2	0
1	92.36	1	42	buccal muco	1	1	0	2	2	0	0	0	0000/00/00	0	2	0
1	35.21	0	36	buccal muco	0	0	1	1	1	0	0	0	0000/00/00	0	2	0
1	4.82	0	71	buccal muco	1	1	1	3	2	1	0	0	0000/00/00	0	2	1
1	103.44	1	65	buccal mucosa				2	2	0	0	0	2013/1/18	1	2	0
1	91.21	1	74	lip	0	0	0	3	3	x	x	0	0000/00/00	0	2	
1	93.80	1	83	alveolar ridge	0	0	0	3	3	0	0	0	0000/00/00	0	1	0
1	73.61	0	70	lip	1	1	0	1	1	0	0	0	0000/00/00	0	2	0
1	90.79	1	41	ant tongue	0	0	0	1	1	x	x	0	0000/00/00	0	2	
1	90.82	1	63	alveolar ridge	0	1	0	4	4	0	0	0	0000/00/00	0	2	0
1	74.56	0	50	buccal muco	1	1	1	1	1	x	x	0	2014/11/11	1	2	
1	90.56	1	61	buccal muco	0	0	0	4	4	0	0	0	0000/00/00	0	1	0
1	2.56	0	75	lip	1	0	0	3	1	1	1	0	0000/00/00	0	2	1
1	90.49	1	44	ant tongue	1	1	1	4	2	2	0	0	0000/00/00	0	2	1
1	90.69	1	67	buccal muco	1	0	0	1	1	0	0	0	0000/00/00	0	2	0
1	65.11	0	53	buccal muco	0	0	0	4	3	2	0	0	2016/8/7	1	2	1
1	91.34	1	54	buccal muco	0	0	0	1	1	0	0	0	2012/7/18	1	2	0
1	90.36	1	80	buccal muco	0	0	0	2	2	0	0	0	0000/00/00	0	1	0
1	47.28	0	61	buccal muco	0	0	0	4	4	0	0	0	0000/00/00	0	2	0
1	90.30	1	81	ant tongue	0	0	0	1	1	x	x	0	2012/2/3	1	2	
1	90.30	1	38	rmt	1	0	1	4	2	2	1	0	0000/00/00	0	2	1
1	90.26	1	59	lip	1	1	1	1	1	x	x	0	0000/00/00	0	2	
1	20.59	0	54	alveolar ridge	0	0	0	4	4	0	0	0	2012/4/25	1	1	0
1	90.33	1	55	lip	0	0	0	1	1	0	0	0	0000/00/00	0	1	0
1	90.72	1	53	buccal muco	0	0	0	1	1	x	x	0	0000/00/00	0	1	
1	89.87	1	64	ant tongue	0	0	0	4	4	0	0	0	0000/00/00	0	2	0
1	89.87	1	46	ant tongue	0	0	0	4	4	2	1	0	2014/4/30	1	2	1
1	90.92	1	35	ant tongue	1	1	0	4	4	2	1	0	2016/7/1	1	2	1
1	90.30	1	55	alveolar ridge	0	0	0	2	2	0	0	0	0000/00/00	0	2	0
1	90.33	1	47	buccal muco	1	1	0	3	3	1	1	0	0000/00/00	0	2	1
1	90.33	1	42	buccal muco	1	1	0	1	1	0	0	0	0000/00/00	0	2	0
1	61.02	0	67	alveolar ridge	0	0	0	4	4	0	0	0	2016/4/22	1	2	0
1	63.64	0	69	ant tongue	1	0	0	1	1	x	x	0	0000/00/00	0	2	
1	28.33	0	56	alveolar ridge	1	1	0	3	3	0	0	0	0000/00/00	0	3	0
1	90.46	1	50	buccal muco	0	0	0	1	1	0	0	0	0000/00/00	0	2	0
1	90.36	1	53	alveolar ridge	0	1	0	1	1	0	0	0	0000/00/00	0	2	0
1	39.44	0	57	ant tongue	1	0	1	4	4	0	0	0	0000/00/00	0	2	0
1	14.69	0	70	ant tongue	0	0	0	2	2	0	0	0	2012/12/11	1	2	0
1	62.62	0	53	hard palate	0	0	0	3	3	1	0	0	0000/00/00	0	2	1
1	89.18	1	40	ant tongue	1	0	1	3	3	0	0	0	0000/00/00	0	2	0
1	89.84	1	40	rmt	0	0	1	1	1	0	0	0	0000/00/00	0	2	0
1	90.23	1	57	rmt	0	0	0	2	2	0	0	0	0000/00/00	0	2	0
1	73.11	0	60	alveolar ridge	0	0	0	4	4	0	0	0	0000/00/00	0	2	0
1	89.61	1	50	ant tongue	1	1	1	4	4	0	0	0	0000/00/00	0	2	0
1	88.98	1	64	mouth floor	0	1	0	2	2	0	0	0	2012/7/6	1	2	0
1	44.82	0	74	rmt	0	0	0	4	4	0	0	0	2014/9/11	1	2	0
1	88.89	1	66	buccal muco	0	0	0	1	1	0	0	0	0000/00/00	0	2	0
1	89.28	1	48	buccal muco	1	1	0	4	4	0	0	0	2012/7/3	1	2	0
1	88.75	1	51	lip	1	1	1	4	1	2	0	0	2012/9/27	1	2	1
1	88.66	1	52	ant tongue	1	1	1	1	1	0	0	0	0000/00/00	0	2	0

1	10.39	0	50	mouth floor	1	1	1	1	1	0	0	1	0000/00/00	0	2	0
1	88.62	1	59	ant tongue	1	1	0	4	4	0	0	0	0000/00/00	0	2	0
1	59.70	0	46	buccal muco	1	0		1	1	x	x	1	0000/00/00	0	1	
1	88.85	1	56	buccal muco	0	0	1	3	3	0	0	0	0000/00/00	0	1	0
1	85.48	0	56	ant tongue	0	0	0	1	1	0	0	0	0000/00/00	0	1	0
1	88.92	1	43	ant tongue	1	1	1	1	1	0	0	0	0000/00/00	0	2	0
1	88.52	1	49	buccal muco	1	1	1	1	1	0	0	0	0000/00/00	0	2	0
1	88.52	1	55	buccal muco	0	1	1	2	2	0	0	0	2012/5/17	1	2	0
1	88.39	1	58	ant tongue	1	1	1	4	4	0	0	0	0000/00/00	0	2	0
1	10.20	0	50	ant tongue	1	1	1	4	4	2	0	0	2012/9/6	1	2	1
1	35.41	0	63	mouth floor	0	0	0	4	4	0	0	0	2013/11/5	1	3	0
1	9.02	0	72	buccal muco	1	0	1	4	2	2	1	0	0000/00/00	0	2	1
1	87.90	1	71	buccal muco	1	0	0	4	4	0	0	0	0000/00/00	0	2	0
1	87.70	1	62	ant tongue	1	0	1	1	1	0	0	0	0000/00/00	0	3	0
1	88.39	1	49	alveolar ridg	1	0	0	4	4	0	0	0	0000/00/00	0	2	0
1	87.77	1	61	ant tongue	1	0	0	2	2	0	0	0	0000/00/00	0	1	0
1	88.49	1	45	buccal muco	1	1	1	無法	1	x	x	0	0000/00/00	0	2	
1	87.54	1	47	buccal muco	1	1	1	2	2	0	0	0	0000/00/00	0	2	0
1	38.75	0	58	alveolar ridg	1	1	1	1	1	x	x	0	0000/00/00	0	1	
1	87.38	1	47	ant tongue	1	1	1	1	1	x	x	0	0000/00/00	0	1	
1	87.34	1	61	buccal muco	1	0	0	4	4	0	0	0	0000/00/00	0	2	0
1	20.92	0	57	buccal muco	0	0	0	1	1	x	x	1	0000/00/00	0	2	
1	87.21	1	53	buccal muco	1	1	0	2	2	0	0	0	0000/00/00	0	2	0
1	87.74	1	55	buccal muco	0	0	0	1	1	0	0	0	0000/00/00	0	1	0
1	87.38	1	52	buccal muco	1	0	1	1	1	x	x	0	2013/12/4	1	2	
1	98.13	1	54	alveolar ridg	1	1	0	1	1	x	x	0	2012/2/14	1	2	
1	87.90	1	61	lip	0	0	1	2	2	0	0	0	0000/00/00	0	2	0
1	87.15	1	60	ant tongue	1	0	1	4	2	2	1	1	0000/00/00	0	2	1
1	0.85	0	63	buccal muco	1	0	1	4	2	2	1	0	0000/00/00	0	3	1
1	87.25	1	60	lip	1	1	0	2	2	0	0	0	0000/00/00	0	2	0
1	87.08	1	38	buccal muco	1	1	1	1	1	x	x	0	0000/00/00	0	2	
1	10.52	0	44	mouth floor	1	1	1	2	2	0	0	0	2012/8/28	1	2	0
1	9.93	0	41	buccal muco	1	1		4	4	x	x	1	2012/4/9	1	2	
1	55.80	0	69	hard palate	1	1	1	1	1	x	x	0	2015/2/11	1	1	
1	86.79	1	79	buccal muco	1	1	1	1	1	0	0	0	0000/00/00	0	2	0
1	96.69	1	37	ant tongue	0	0	0	2	2	0	0	1	2012/3/1	1	1	0
1	48.10	0	52	ant tongue	1	1	1	3	3	0	0	0	0000/00/00	0	2	0
1	32.56	0	59	mouth floor	1	1	1	2	2	0	0	0	0000/00/00	0	1	0
1	52.07	0	54	buccal muco	1	1	1	2	2	0	0	0	0000/00/00	0	2	0
1	7.48	0	63	mouth floor	1	1	1	1	1	0	0	0	0000/00/00	0	2	0
1	42.92	0	76	lip	1	1	0	2	2	x	x	0	0000/00/00	0	2	
1	35.93	0	68	ant tongue	1	1	1	1	1	0	0	0	0000/00/00	0	2	0
1	86.43	1	75	lip	1	1	1	2	2	0	0	0	0000/00/00	0	2	0
1	86.46	1	45	alveolar ridg	1	1	0	1	1	x	x	0	0000/00/00	0	2	
1	31.05	0	73	buccal muco	0	0	0	1	1	x	x	0	0000/00/00	0	1	
1	86.39	1	58	alveolar ridg	1	1	1	無法	1	x	x	0	0000/00/00	0	2	
1	16.75	0	79	buccal muco	0	0	0	2	2	x	x	1	0000/00/00	0	2	
1	86.89	1	50	alveolar ridg	1	1	1	4	4	2	0	0	2012/8/14	1	2	1
1	86.46	1	43	lip	1	1	1	1	1	0	0	0	0000/00/00	0	2	0
1	86.43	1	48	lip	1	0	1	1	1	0	0	0	0000/00/00	0	2	0
1	48.66	0	51	ant tongue	1	0	1	1	1	0	0	0	0000/00/00	0	2	0
1	87.08	1	45	buccal muco	1	1	1	3	2	1	1	1	0000/00/00	0	2	1
1	86.69	1	45	ant tongue	1	1	1	4	1	2	0	0	0000/00/00	0	2	1
1	86.36	1	55	buccal muco	1	0	1	1	1	0	0	0	0000/00/00	0	2	0
1	17.51	0	76	alveolar ridg	1	1	0	4	4	0	0	0	2012/7/16	1	2	0
1	86.89	1	46	buccal muco	1	0		1	1	x	x	0	0000/00/00	0	2	
1	9.74	0	45	lip	0	1	1	4	4	2	1	0	2012/11/5	1	2	1
1	86.23	1	44	buccal muco	1	1	1	4	4	2	1	0	0000/00/00	0	3	1
1	86.79	1	55	lip	1	1	1	無法	3	x	x	0	0000/00/00	0	1	
1	86.07	1	62	buccal muco	1	0	0	1	1	x	x	1	0000/00/00	0	2	
1	86.16	1	77	buccal muco	0	0	1	1	1	x	x	0	0000/00/00	0	2	
1	36.33	0	32	ant tongue	1	1	1	4	4	2	1	0	0000/00/00	0	2	1
1	87.48	1	74	buccal muco	1	1	1	1	1	x	x	0	0000/00/00	0	1	
1	86.66	1	54	buccal muco	1	1	1	4	4	0	0	1	0000/00/00	0	1	0
1	85.93	1	61	buccal muco	1	0	0	2	2	0	0	0	2016/1/18	1	2	0
1	85.97	1	56	buccal muco	1	0	1	1	1	0	0	0	0000/00/00	0	1	0
1	38.49	0	73	ant tongue	1	1		3	3	0	0	0	0000/00/00	0	1	0
1	85.93	1	61	ant tongue	1	0	1	3	2	1	0	0	0000/00/00	0	2	1
1	86.56	1	52	buccal muco	1	0	0	2	2	0	0	0	0000/00/00	0	1	0
1	85.74	1	57	alveolar ridg	0	0	0	1	1	x	x	0	0000/00/00	0	1	
1	76.82	0	68	ant tongue	0	0	0	1	1	x	x	0	0000/00/00	0	2	
1	4.39	0	74	alveolar ridg	1	0	0	4	4	2	1	0	0000/00/00	0	2	1

1	49.38	0	56	ant tongue	0	0	0	4	4	1	0	0	0000/00/00	0	2	1
1	20.85	0	56	ant tongue	0	0	0	1	1	0	0	0	0000/00/00	0	2	0
1	85.67	1	61	ant tongue	1	0		2	2	0	0	0	0000/00/00	0	2	0
1	10.95	0	60	buccal muco	0	0	0	2	2	x	x	0	0000/00/00	0	2	
1	5.57	0	84	ant tongue	0	0	0	2	2	x	x	0	0000/00/00	0	2	
1	86.43	1	62	lip	0	0	0	1	1	x	x	0	0000/00/00	0	2	
1	95.18	1	58	buccal muco	1	1	0	1	1	0	0	0	2014/8/11	1	2	0
1	85.28	1	51	buccal muco	1	0	1	2	2	x	x	0	0000/00/00	0	2	
1	62.85	0	63	lip	0	0	0	2	2	x	x	0	0000/00/00	0	2	
1	18.43	0	55	ant tongue	0	1	0	4	4	1	0	0	2013/1/4	1	2	1
1	85.67	1	47	alveolar ridge	1	1	1	1	1	x	x	0	0000/00/00	0	1	
1	85.15	1	59	ant tongue	1	1	1	2	2	0	0	0	0000/00/00	0	2	0
1	6.46	0	49	ant tongue	0	0	0	4	2	2	1	0	0000/00/00	0	2	1
1	85.02	1	59	buccal muco	1	0	0	2	2	0	0	0	2013/7/29	1	2	0
1	83.80	0	41	ant tongue	1	1	1	4	4	0	0	0	0000/00/00	0	2	0
1	85.51	1	70	ant tongue	0	0	0	1	1	x	x	0	2013/3/22	1	2	
1	82.23	0	44	alveolar ridge	1	1	0	2	2	0	0	0	0000/00/00	0	2	0
1	84.92	1	48	buccal muco	1	1	1	3	1	1	0	0	0000/00/00	0	2	1
1	50.59	0	59	buccal muco	1	0	0	4	4	0	0	0	2015/4/14	1	2	0
1	22.49	0	68	alveolar ridge	1	1	0	2	2	0	0	0	0000/00/00	0	2	0
1	85.05	1	57	lip	0	0	0	1	1	x	x	0	2013/11/26	1	2	
1	85.25	1	41	lip	1	1	0	2	2	0	0	0	0000/00/00	0	2	0
1	23.90	0	44	buccal muco	0	0	0	1	1	0	0	0	2013/9/6	1	2	0
1	26.26	0	74	alveolar ridge	1	0	0	4	4	x	x	0	0000/00/00	0	2	
1	85.02	1	61	buccal muco	1	0	0	無法	2	x	x	0	0000/00/00	0	1	
1	21.28	0	55	ant tongue	1	0	0	1	1	0	0	0	0000/00/00	0	2	0
1	84.36	1	45	mouth floor	1	1	1	4	4	0	0	0	0000/00/00	0	2	0
1	7.11	0	48	ant tongue	1	1	0	4	4	2	1	0	2012/11/12	1	2	1
1	4.79	0	33	ant tongue	1	0	1	4	4	2	1	0	0000/00/00	0	2	1
1	84.49	1	55	ant tongue	0	0	0	4	4	0	0	0	0000/00/00	0	2	0
1	84.30	1	44	buccal muco	0	0	0	1	1	0	0	0	0000/00/00	0	1	0
1	84.79	1	75	ant tongue	0	0	0	2	2	0	0	0	0000/00/00	0	2	0
1	84.59	1	54	buccal muco	0	0	1	1	1	x	x	0	0000/00/00	0	1	
1	5.05	0	59	mouth floor	0	0	0	4	4	1	1	0	2012/9/10	1	2	1
1	84.62	1	38	buccal muco	0	1	0	2	2	0	0	0	0000/00/00	0	2	0
1	27.67	0	66	rmt	0	0	0	2	2	0	0	0	0000/00/00	0	2	0
1	83.80	1	58	buccal muco	1	1	0	1	1	0	0	0	0000/00/00	0	2	0
1	45.67	0	71	rmt	0	1	0	4	4	0	0	0	0000/00/00	0	2	0
1	8.23	0	50	buccal muco	1	0	0	4	4	2	1	1	0000/00/00	0	2	1
1	15.87	0	55	buccal muco	1	0	0	4	4	2	0	1	0000/00/00	0	3	1
1	8.13	0	42	ant tongue	1	1	0	4	4	2	1	0	0000/00/00	0	3	1
1	83.21	1	51	buccal muco	1	1	0	1	1	0	0	0	0000/00/00	0	2	0
1	11.28	0	51	buccal muco	1	0	1	1	1	x	x	0	0000/00/00	0	2	
1	83.02	1	78	ant tongue	0	0	0	1	1	x	x	0	0000/00/00	0	2	
1	28.66	0	69	ant tongue	1	0	0	3	3	1	1	0	0000/00/00	0	2	1
1	8.69	0	53	buccal muco	0	0	0	4	2	2	1	0	2013/1/24	1	3	1
1	82.89	1	44	ant tongue	1	1	1	4	4	0	0	0	0000/00/00	0	2	0
1	83.02	1	36	ant tongue	1	1	1	1	1	0	0	0	0000/00/00	0	1	0
1	77.02	0	48	rmt	1	1	1	4	2	2	1	0	0000/00/00	0	2	1
1	84.36	1	63	alveolar ridge	1	1	1	1	1	0	0	0	0000/00/00	0	2	0
1	18.00	0	74	hard palate	1	0	1	1	1	x	x	1	2013/3/13	1	2	
1	83.48	1	47	ant tongue	1	1	1	1	1	x	x	0	0000/00/00	0	1	
1	82.52	1	60	buccal muco	0	0	0	4	4	0	0	0	0000/00/00	0	2	0
1	81.11	0	55	alveolar ridge	1	0	1	無法	1	x	x	0	2014/8/5	1	1	
1	82.46	1	81	lip	1	0	0	1	1	0	0	0	0000/00/00	0	2	0
1	82.46	1	69	buccal muco	0	0	0	1	1	0	0	0	0000/00/00	0	2	0
1	82.95	1	41	hard palate	0	0	1	3	3	0	0	0	0000/00/00	0	2	0
1	83.41	1	56	buccal muco	0	0	0	4	4	0	0	0	0000/00/00	0	2	0
1	82.33	1	52	ant tongue	1	1	1	2	2	0	0	0	0000/00/00	0	2	0
1	55.70	0	65	buccal muco	1	1	0	3	3	x	x	0	0000/00/00	0	1	
1	11.31	0	67	ant tongue	0	0	0	1	1	0	0	0	2013/1/19	1	2	0
1	82.26	1	59	buccal muco	1	0	1	4	4	1	0	0	0000/00/00	0	2	1
1	83.11	1	55	rmt	1	1	0	2	2	0	0	0	2013/8/10	1	1	0
1	11.02	0	46	ant tongue	0	0	0	3	2	1	0	0	2013/1/25	1	2	1
1	82.52	1	65	buccal muco	0	0	0	1	1	0	0	0	2012/9/10	1	2	0
1	81.74	1	40	buccal muco	1	1	1	1	1	0	0	0	2013/9/26	1	2	0
1	81.64	1	50	rmt	1	1	1	4	4	0	0	0	0000/00/00	0	1	0
1	81.61	1	55	alveolar ridge	1	0	0	4	4	0	0	0	0000/00/00	0	2	0
1	15.61	0	56	buccal muco	1	1	0	4	2	2	1	0	0000/00/00	0	2	1
1	50.46	0	79	ant tongue	0	0	0	2	2	0	0	0	2013/5/7	1	2	0
1	10.03	0	87	lip	1	0	0	2	2	x	x	0	0000/00/00	0	2	
1	81.34	1	60	buccal muco	1	1	1	1	1	x	x	0	0000/00/00	0	2	

1	3.84	0	53	buccal muco	0	0	1	3	3	1	0	0	2012/9/28	1	3	1
1	81.28	1	42	buccal muco	0	0	0	2	2	0	0	0	0000/00/00	0	2	0
1	81.31	1	51	buccal muco	0	0	0	1	1	x	x	0	2015/1/14	1	2	
1	49.74	0	47	ant tongue	1	0		2	2	0	0	0	2014/5/13	1	3	0
1	81.15	1	61	alveolar ridg	0	0	0	1	1	0	0	0	0000/00/00	0	1	0
1	65.31	0	61	buccal muco	0	0	0	3	1	1	1	0	0000/00/00	0	2	1
1	58.07	0	40	ant tongue	1	1	1	3	1	1	0	0	0000/00/00	0	2	1
1	0.82	0	69	ant tongue	1	0	0	1	1	0	0	0	0000/00/00	0	2	0
1	81.51	1	60	buccal muco	1	1	0	4	4	0	0	0	0000/00/00	0	2	0
1	80.79	1	53	lip	0	0	0	1	1	x	x	0	0000/00/00	0	2	
1	80.66	1	46	buccal muco	0	0	0	1	1	0	0	0	0000/00/00	0	2	0
1	29.08	0	64	buccal muco	1	1	1	3	3	0	0	0	0000/00/00	0	2	0
1	80.59	1	70	ant tongue	0	0	0	2	2	0	0	0	0000/00/00	0	2	0
1	80.56	1	65	mouth floor	1	1	1	4	4	2	1	0	0000/00/00	0	2	1
1	45.87	0	59	lip	0	0	0	4	4	0	0	0	0000/00/00	0	2	0
1	10.30	0	53	alveolar ridg	1	0	0	4	4	0	0	0	2013/3/1	1	2	0
1	11.64	0	55	buccal muco	0	0	0	4	4	2	1	1	2013/2/20	1	2	1
1	80.33	1	56	alveolar ridg	0	1	1	1	1	x	x	1	0000/00/00	0	2	
1	17.54	0	37	buccal muco	1	0	1	4	4	0	0	0	2013/2/12	1	1	0
1	26.23	0	59	buccal muco	1	1	1	1	1	x	x	0	0000/00/00	0	2	
1	41.02	0	62	ant tongue	1	1	1	2	2	0	0	0	0000/00/00	0	2	0
1	80.16	1	41	alveolar ridg	0	1	0	2	2	0	0	0	0000/00/00	0	2	0
1	80.20	1	38	buccal muco	1	1	1	4	2	2	0	0	0000/00/00	0	3	1
1	10.85	0	71	ant tongue	1	1	1	1	1	0	0	0	2013/3/4	1	2	0
1	23.70	0	67	ant tongue	0	0	0	3	2	1	1	0	0000/00/00	0	2	1
1	48.39	0	58	lip	0	0	0	3	3	0	0	0	0000/00/00	0	1	0
1	20.62	0	65	alveolar ridg	0	0	0	4	4	0	0	0	0000/00/00	0	2	0
1	47.48	0	51	ant tongue	1	0	1	1	1	0	0	0	0000/00/00	0	2	0
1	79.90	1	48	buccal muco	1	0	0	1	1	0	0	0	0000/00/00	0	2	0
1	80.59	1	59	buccal muco	0	0	0	1	1	0	0	0	0000/00/00	0	1	0
1	79.87	1	39	ant tongue	1	0		1	1	0	0	0	0000/00/00	0	2	0
1	18.30	0	48	ant tongue	1	0	0	3	1	1	0	0	2013/6/19	1	1	1
1	7.34	0	60	ant tongue	1	0	1	3	3	0	0	0	0000/00/00	0	3	0
1	81.31	1	67	alveolar ridg	1	0		4	4	0	0	0	0000/00/00	0	2	0
1	33.21	0	50	ant tongue	1	1		2	2	0	0	0	0000/00/00	0	2	0
1	79.44	1	62	alveolar ridg	1	1	0	4	4	0	0	0	0000/00/00	0	2	0
1	81.28	1	43	ant tongue	1	0	1	1	1	0	0	0	0000/00/00	0	3	0
1	79.48	1	59	buccal muco	1	1	1	1	1	0	0	0	0000/00/00	0	2	0
1	47.57	0	47	ant tongue	1	0	0	2	2	0	0	0	2016/2/11	1	3	0
1	44.89	0	49	buccal muco	1	0	1	4	4	0	0	0	0000/00/00	0	2	0
1	79.21	1	69	alveolar ridg	1	1	0	4	4	2	1	1	0000/00/00	0	2	1
1	8.07	0	73	alveolar ridg	1	1	1	4	4	2	0	0	0000/00/00	0	3	1
1	16.00	0	75	alveolar ridg	0	0	0	4	4	2	1	0	2014/1/15	1	2	1
1	79.28	1	40	alveolar ridg	1	0	0	1	1	0	0	0	0000/00/00	0	2	0
1	90.79	1	41	buccal muco	0	0	0	1	1	x	x	0	0000/00/00	0	2	
1	79.21	1	49	ant tongue	1	0	0	3	3	0	0	0	0000/00/00	0	2	0
1	79.54	1	45	lip	1	1		1	1	x	x	0	2016/1/9	1	1	
1	7.67	0	50	buccal muco	1	0	1	1	1	0	0	0	0000/00/00	0	2	0
1	78.98	1	62	alveolar ridg	1	0	0	4	4	0	0	0	0000/00/00	0	1	0
1	78.98	1	67	lip	0	0	1	1	1	x	x	0	0000/00/00	0	1	
1	79.54	1	40	buccal muco	1	0	1	2	2	0	0	0	0000/00/00	0	2	0
1	31.08	0	50	rmt	0	0	0	4	4	0	0	0	0000/00/00	0	2	0
1	78.49	1	53	buccal muco	1	0	1	1	1	0	0	0	0000/00/00	0	1	0
1	6.39	0	63	alveolar ridg	1	0	0	4	4	2	1	0	0000/00/00	0	2	1
1	78.36	1	46	ant tongue	1	0	1	1	1	0	0	0	0000/00/00	0	2	0
1	43.64	0	48	buccal muco	1	0	0	4	4	0	0	1	2014/5/8	1	1	0
1	78.30	1	56	buccal muco	0	0	0	4	4	0	0	0	0000/00/00	0	2	0
1	78.62	1	56	buccal muco	1	1	0	1	1	x	x	0	0000/00/00	0	1	
1	79.31	1	60	buccal muco	0	0		4	4	0	0	0	0000/00/00	0	1	0
1	78.79	1	44	ant tongue	0	0	1	2	2	0	0	0	0000/00/00	0	2	0
1	79.21	1	65	rmt	0	1	1	3	3	0	0	0	0000/00/00	0	2	0
1	29.80	0	50	alveolar ridg	1	0	0	4	4	0	0	0	0000/00/00	0	2	0
1	78.36	1	56	lip	1	0	0	3	2	1	0	0	0000/00/00	0	1	1
1	6.20	0	49	alveolar ridg	1	1	1	4	4	2	1	0	2013/5/7	1	2	1
1	77.97	1	44	ant tongue	1	0	1	4	4	0	0	0	0000/00/00	0	2	0
1	78.62	1	49	alveolar ridg	1	1	1	4	4	0	0	0	0000/00/00	0	1	0
1	78.62	1	60	ant tongue	0	0	1	2	2	0	0	0	0000/00/00	0	2	0
1	36.82	0	70	ant tongue	1	1	1	2	2	0	0	0	0000/00/00	0	2	0
1	9.93	0	42	ant tongue	1	1	1	3	3	1	0	0	2013/5/27	1	2	1
1	77.90	1	69	lip	0	0	0	1	1	0	0	0	0000/00/00	0	1	0
1	40.03	0	72	alveolar ridg	0	1	0	4	4	0	0	0	0000/00/00	0	2	0
1	78.13	0	61	buccal muco	0	1	1	4	4	1	0	0	2016/6/6	1	2	1

1	77.84	1	72	lip	0	0	0	1	1	0	0	0	0000/00/00	0	2	0
1	18.52	0	51	ant tongue	1	1	0	1	1	0	0	0	0000/00/00	0	2	0
1	2.43	0	61	buccal muco	0	0	0	4	4	0	0	0	0000/00/00	0	2	0
1	3.74	0	76	buccal muco	0	0	0	4	4	0	0	0	0000/00/00	0	2	0
1	77.48	1	54	alveolar ridge	1	1	1	4	4	0	0	0	0000/00/00	0	2	0
1	21.05	0	51	ant tongue	1	0	0	2	2	0	0	0	2013/7/18	1	1	0
1	77.25	1	44	buccal muco	1	0	0	1	1	0	0	0	0000/00/00	0	2	0
1	77.15	1	51	alveolar ridge	0	0	0	4	4	0	0	0	0000/00/00	0	2	0
1	38.20	0	48	ant tongue	1	0	0	1	1	0	0	0	2015/7/9	1	2	0
1	77.44	1	58	buccal muco	0	0	0	2	2	0	0	0	2014/2/12	1	2	0
1	44.98	0	64	ant tongue	0	0	1	1	1	0	0	0	0000/00/00	0	2	0
1	77.28	1	73	buccal muco	1	0	0	1	1	x	x	0	2014/10/13	1	1	
1	76.98	1	57	buccal muco	1	1	0	4	2	2	1	0	0000/00/00	0	2	1
1	76.92	1	67	alveolar ridge	0	0	0	1	1	x	x	0	0000/00/00	0	2	
1	77.67	1	55	buccal muco	0	0	0	3	3	0	0	0	2013/12/6	1	2	0
1	78.30	1	53	alveolar ridge	1	1	1	4	4	2	0	0	0000/00/00	0	2	1
1	77.51	1	48	buccal muco	0	0	0	1	1	0	0	0	0000/00/00	0	1	0
1	15.80	0	70	buccal muco	0	0	0	3	1	1	0	1	0000/00/00	0	2	1
1	24.85	0	48	alveolar ridge	1	0	0	4	2	2	0	0	2014/2/20	1	2	1
1	48.89	0	69	alveolar ridge	0	0	0	1	1	x	x	0	2013/8/14	1	1	
1	24.49	0	48	alveolar ridge	1	0	1	4	4	0	0	0	0000/00/00	0	2	0
1	18.75	0	45	buccal muco	1	1	1	4	4	2	0	0	0000/00/00	0	2	1
1	76.36	1	54	buccal muco	0	0	0	1	1	x	x	0	0000/00/00	0	1	
1	77.70	1	73	alveolar ridge	1	1	0	2	2	0	0	0	0000/00/00	0	1	0
1	8.98	0	52	ant tongue	1	1	1	4	4	2	1	0	2013/6/25	1	2	1
1	78.16	1	53	buccal muco	0	0	0	1	1	x	x	0	2016/3/29	1	2	
1	6.26	0	46	ant tongue	1	1		4	4	2	1	0	0000/00/00	0	2	1
1	13.02	0	46	alveolar ridge	1	0	0	4	3	2	1	0	2013/8/13	1	2	1
1	76.49	1	47	buccal muco	1	1	0	1	1	0	0	0	0000/00/00	0	3	0
1	17.87	0	54	ant tongue	1	1	1	1	1	0	0	0	0000/00/00	0	2	0
1	76.26	1	47	buccal muco	0	0	0	1	1	x	x	0	2013/3/9	1	2	
1	54.98	0	70	alveolar ridge	1	0	0	1	1	0	0	0	0000/00/00	0	2	0
1	76.00	1	54	buccal muco	1	1	1	2	2	0	0	0	0000/00/00	0	1	0
1	76.03	1	73	buccal muco	0	0	0	2	2	0	0	0	0000/00/00	0	2	0
1	11.48	0	40	alveolar ridge	0	1	0	4	4	x	x	1	0000/00/00	0	2	
1	75.77	1	65	ant tongue	1	0	0	1	1	0	0	0	0000/00/00	0	1	0
1	77.11	1	60	buccal muco	1	1	0	1	1	0	0	0	0000/00/00	0	1	0
1	75.67	1	60	lip	0	0	0	1	1	0	0	0	0000/00/00	0	2	0
1	11.25	0	64	alveolar ridge	0	0	0	4	4	0	0	0	0000/00/00	0	2	0
1	78.72	1	60	buccal muco	1	0	0	2	2	0	0	0	0000/00/00	0	2	0
1	55.74	0	64	buccal muco	1	0	0	1	1	0	0	0	0000/00/00	0	1	0
1	75.34	1	62	ant tongue	0	0	0	2	2	0	0	0	0000/00/00	0	2	0
1	76.10	1	50	alveolar ridge	1	1	1	4	4	0	0	0	2018/3/7	1	2	0
1	15.25	0	74	buccal muco	1	1	0	4	3	2	1	0	2013/11/6	1	3	1
1	75.31	1	52	rmt	0	0	0	1	1	x	x	0	0000/00/00	0	2	
1	35.38	0	44	ant tongue	1	1		4	4	2	1	1	0000/00/00	0	2	1
1	58.07	0	61	lip	1	0	0	1	1	0	0	0	0000/00/00	0	2	0
1	75.28	1	63	buccal muco	1	0	0	4	4	0	0	0	0000/00/00	0	2	0
1	75.11	1	62	alveolar ridge	1	1	1	4	4	x	x	0	0000/00/00	0	2	
1	74.98	1	61	ant tongue	1	0	1	1	1	0	0	0	0000/00/00	0	2	0
1	74.89	1	52	alveolar ridge	1	0	1	4	4	2	0	0	0000/00/00	0	2	1
1	75.87	1	39	buccal muco	0	0	0	2	2	0	0	0	0000/00/00	0	2	0
1	33.21	0	43	buccal muco	1	0		2	2	0	0	0	2013/5/27	1	2	0
1	74.98	1	51	buccal muco	1	0	1	2	2	0	0	0	0000/00/00	0	2	0
1	34.69	0	69	lip	1	0	0	1	1	0	0	0	2015/8/27	1	2	0
1	74.46	1	55	mouth floor	1	1	1	1	1	0	0	0	2016/11/23	1	2	0
1	74.49	1	52	buccal muco	1	0	1	1	1	x	x	0	0000/00/00	0	1	
1	74.39	1	52	lip	1	1	1	4	4	0	0	0	0000/00/00	0	2	0
1	34.56	0	76	ant tongue	1	0	1	4	4	0	0	0	0000/00/00	0	2	0
1	42.26	0	69	buccal muco	1	1	1	2	2	0	0	0	0000/00/00	0	1	0
1	2.89	0	46	buccal muco	1	1	1	4	4	2	1	0	2013/5/6	1	2	1
1	74.26	1	51	ant tongue	1	0	1	4	4	2	1	0	0000/00/00	0	2	1
1	74.43	1	57	buccal muco	1	0	0	4	4	0	0	0	0000/00/00	0	2	0
1	75.15	1	77	mouth floor	0	0	0	1	1	0	0	0	0000/00/00	0	2	0
1	74.16	1	45	ant tongue	1	1		1	1	0	0	0	0000/00/00	0	2	0
1	30.49	0	40	ant tongue	0	0	0	3	2	1	0	0	2015/9/9	1	2	1
1	32.39	0	40	mouth floor	1	0	1	1	1	0	0	0	2015/4/30	1	2	0
1	74.66	1	52	ant tongue	1	0	1	1	1	0	0	0	0000/00/00	0	2	0
1	74.39	1	52	buccal muco	0	0	0	2	2	0	0	0	2016/12/16	1	2	0
1	74.03	1	60	alveolar ridge	1	1	0	1	1	0	0	0	0000/00/00	0	2	0
1	17.97	0	50	ant tongue	1	0	1	1	1	0	0	0	2014/5/5	1	1	0
1	74.59	1	52	hard palate	1	0	1	3	3	0	0	0	0000/00/00	0	1	0

1	76.26	1	68	lip	0	0	1	1	1	x	x	0	0000/00/00	0	2	
1	30.16	0	69	alveolar ridg	1	0	0	2	2	0	0	0	2015/6/8	1	1	0
1	34.36	0	54	ant tongue	0	0	0	4	4	2	0	0	0000/00/00	0	2	1
1	5.70	0	64	buccal muco	0	0	0	3	3	0	0	0	2013/8/6	1	2	0
1	85.48	1	43	ant tongue	1	1	1	1	1	0	0	0	0000/00/00	0	2	0
1	22.72	0	59	alveolar ridg	1	0	0	4	4	1	0	0	0000/00/00	0	2	1
1	73.77	1	63	mouth floor	1	0		1	1	0	0	0	0000/00/00	0	2	0
1	10.20	0	49	buccal muco	1	1	1	4	4	0	0	1	0000/00/00	0	1	0
1	74.23	1	49	buccal muco	1	1	0	4	2	2	1	0	0000/00/00	0	2	1
1	73.61	1	45	buccal muco	0	0	1	3	1	1	1	0	0000/00/00	0	2	1
1	73.80	1	52	buccal muco	0	0	0	2	2	0	0	0	0000/00/00	0	2	0
1	32.26	0	73	buccal muco	1	1	0	3	1	1	1	0	2015/10/15	1	2	1
1	16.43	0	39	ant tongue	0	0	0	4	2	2	1	0	2014/4/26	1	2	1
1	74.23	1	78	lip	0	0	1	1	1	x	x	0	0000/00/00	0	1	
1	73.51	1	46	ant tongue	0	1	1	1	1	0	0	0	0000/00/00	0	2	0
1	73.31	1	57	ant tongue	1	0	0	1	1	0	0	0	0000/00/00	0	1	0
1	63.18	0	78	alveolar ridg	1	0	0	4	4	0	0	0	2017/10/3	1	1	0
1	73.70	1	43	ant tongue	1	0	0	1	1	x	x	0	0000/00/00	0	3	
1	73.31	1	62	rmt	1	1	0	4	4	0	0	0	0000/00/00	0	2	0
1	73.48	1	40	buccal muco	1	0	1	4	4	2	1	0	0000/00/00	0	2	1
1	20.30	0	59	rmt	1	1		4	4	2	1	0	0000/00/00	0	2	1
1	27.21	0	69	buccal muco	1	1	0	1	1	x	x	0	0000/00/00	0	2	
1	72.89	1	57	buccal muco	0	0	1	4	4	1	1	0	0000/00/00	0	2	1
1	34.20	0	48	buccal muco	1	1	0	1	1	0	0	0	2014/5/19	1	2	0
1	76.56	1	62	alveolar ridg	0	0	0	1	1	0	0	0	0000/00/00	0	1	0
1	72.85	1	49	ant tongue	1	0	1	3	3	1	0	0	0000/00/00	0	2	1
1	72.92	1	64	buccal muco	0	0	0	4	2	2	1	0	0000/00/00	0	2	1
1	74.49	1	79	buccal muco	1	0	0	1	1	0	0	0	0000/00/00	0	1	0
1	38.23	0	44	ant tongue	0	0	0	1	1	0	0	0	0000/00/00	0	2	0
1	72.75	1	61	ant tongue	1	1	1	無法	1	x	x	0	0000/00/00	0	1	
1	72.69	1	54	buccal muco	1	1		4	2	2	1	0	0000/00/00	0	2	1
1	72.69	1	52	buccal muco	0	0	0	2	2	0	0	0	0000/00/00	0	2	0
1	73.15	1	57	buccal muco	1	1	1	3	3	0	0	0	0000/00/00	0	1	0
1	72.56	1	66	ant tongue	1	1	1	4	4	2	1	0	0000/00/00	0	2	1
1	73.08	1	52	mouth floor	1	1	0	2	2	0	0	0	0000/00/00	0	2	0
1	30.56	0	50	ant tongue	1	0	0	3	1	1	0	0	2014/4/29	1	1	1
1	72.59	1	50	alveolar ridg	1	0	1	4	4	0	0	0	0000/00/00	0	2	0
1	18.49	0	48	ant tongue	1	1	1	2	2	0	0	0	2014/5/13	1	2	0
1	52.26	0	69	hard palate	0	1	0	4	4	x	x	0	2017/2/10	1	2	
1	72.69	1	70	ant tongue	1	1	1	1	1	x	x	0	0000/00/00	0	2	
1	72.33	1	51	buccal muco	1	0	0	4	4	2	1	0	0000/00/00	0	2	1
1	72.46	1	47	ant tongue	1	1	1	2	2	0	0	0	0000/00/00	0	2	0
1	25.80	0	55	ant tongue	1	1	1	1	1	x	x	0	0000/00/00	0	2	
1	53.15	0	40	ant tongue	1	1	0	2	2	0	0	0	0000/00/00	0	2	0
1	27.11	0	57	buccal muco	1	0	0	2	2	x	x	0	0000/00/00	0	1	
1	71.97	1	73	buccal muco	0	0	0	1	1	0	0	0	2015/2/4	1	2	0
1	72.30	1	55	ant tongue	1	1		1	1	0	0	0	0000/00/00	0	2	0
1	71.97	1	69	ant tongue	0	0	0	1	1	x	x	0	0000/00/00	0	2	
1	71.87	1	63	buccal muco	0	0	1	2	2	0	0	0	0000/00/00	0	2	0
1	4.07	0	67	hard palate	0	0	0	2	2	x	x	0	0000/00/00	0	2	
1	42.46	0	72	ant tongue	0	0	0	2	2	0	0	0	0000/00/00	0	3	0
1	71.64	1	68	alveolar ridg	1	1	1	4	4	x	x	0	0000/00/00	0	1	
1	71.93	1	59	alveolar ridg	0	0	0	1	1	x	x	0	0000/00/00	0	2	
1	6.89	0	66	alveolar ridg	1	0	1	4	4	2	1	0	0000/00/00	0	2	1
1	7.44	0	62	buccal muco	0	0	0	4	4	x	x	0	0000/00/00	0	2	
1	71.41	1	53	rmt	1	0	0	2	2	x	x	0	0000/00/00	0	2	
1	38.79	0	72	alveolar ridg	1	1	0	1	1	0	0	0	0000/00/00	0	2	0
1	71.21	1	44	lip	1	1	1	2	2	0	0	0	0000/00/00	0	2	0
1	71.70	1	81	buccal muco	1	1	0	4	2	2	1	0	0000/00/00	0	2	1
1	71.15	1	61	buccal muco	1	1	1	1	1	x	x	0	2014/9/13	1	2	
1	71.05	1	55	alveolar ridg	1	1	1	1	1	x	x	0	0000/00/00	0	2	
1	70.85	1	60	alveolar ridg	0	0	0	4	4	2	1	0	0000/00/00	0	1	1
1	18.56	0	67	mouth floor	0	0	0	2	2	x	x	0	2013/12/4	1	2	
1	49.64	0	70	buccal muco	1	0	0	無法	1	x	0	0	0000/00/00	0	2	
1	70.62	1	51	mouth floor	0	0	0	1	1	0	0	0	0000/00/00	0	2	0
1	70.62	1	53	alveolar ridg	1	1	0	1	1	0	0	0	0000/00/00	0	2	0
1	6.92	0	52	buccal muco	1	1	0	4	4	x	x	0	2013/10/9	1	2	
1	70.59	1	48	ant tongue	1	1	0	1	1	0	0	0	0000/00/00	0	2	0
1	71.15	1	63	ant tongue	1	1	1	2	2	0	0	0	0000/00/00	0	1	0
1	70.39	1	74	ant tongue	0	0	0	2	2	x	x	0	0000/00/00	0	2	
1	70.23	0	75	buccal muco	1	1	0	4	4	0	0	0	0000/00/00	0	1	0
1	6.20	0	53	ant tongue	1	1	0	4	4	2	1	0	2013/11/1	1	2	1

1	70.16	1	49	lip	1	1	1	2	2	x	x	0	0000/00/00	0	1	
1	70.03	1	52	lip	1	0	1	4	4	1	0	0	0000/00/00	0	2	1
1	70.59	1	37	buccal muco	1	1	0	1	1	0	0	0	2013/10/11	1	1	0
1	70.03	1	68	ant tongue	1	1	0	2	2	0	0	0	2014/9/10	1	2	0
1	46.36	0	64	hard palate	0	0	0	4	4	0	0	0	0000/00/00	0	2	0
1	69.67	1	66	buccal muco	1	1	0	2	2	0	0	0	0000/00/00	0	2	0
1	70.16	1	33	alveolar ridge	1	0	1	1	1	0	0	0	0000/00/00	0	2	0
1	42.10	0	60	ant tongue	0	0	1	1	1	x	x	0	2014/4/3	1	2	
1	69.61	1	56	buccal muco	0	0	0	1	1	x	x	0	2014/1/24	1	2	
1	69.44	1	40	ant tongue	1	0	1	1	1	0	0	0	0000/00/00	0	2	0
1	81.61	0	44	buccal muco	1	1	0	1	1	0	0	0	0000/00/00	0	2	0
1	69.70	1	71	buccal muco	0	0	0	1	1	0	0	0	0000/00/00	0	1	0
1	69.84	1	38	rmt	1	1		2	2	0	0	0	0000/00/00	0	2	0
1	69.31	1	53	ant tongue	1	1	1	2	2	0	0	0	0000/00/00	0	2	0
1	70.23	1	58	lip	0	0	0	3	1	1	1	0	0000/00/00	0	2	1
1	69.18	1	62	ant tongue	1	1	0	2	2	0	0	0	0000/00/00	0	2	0
1	69.67	1	45	ant tongue	1	0	1	1	1	x	x	0	0000/00/00	0	2	
1	69.80	1	40	ant tongue	1	1	0	1	1	0	0	0	0000/00/00	0	2	0
1	69.15	1	76	buccal muco	0	0	0	1	1	x	x	0	2013/10/2	1	1	
1	8.82	0	74	lip	1	1	0	4	3	2	1	0	2013/11/6	1	3	1
1	68.95	1	44	buccal muco	1	1	1	3	3	0	0	0	0000/00/00	0	2	0
1	11.70	0	61	hard palate	0	0	0	1	1	x	x	0	2014/3/27	1	1	
1	68.69	1	57	ant tongue	1	0	0	1	1	0	0	0	0000/00/00	0	2	0
1	68.72	1	56	buccal muco	0	0	0	2	2	0	0	0	0000/00/00	0	2	0
1	68.66	1	62	buccal muco	0	0	0	2	2	0	0	0	0000/00/00	0	2	0
1	69.25	1	46	buccal muco	1	0	1	1	1	x	x	0	0000/00/00	0	1	
1	57.05	0	62	alveolar ridge	1	0	0	4	4	0	0	0	2017/8/17	1	1	0
1	8.23	0	53	ant tongue	1	0	0	4	4	2	1	0	0000/00/00	0	2	1
1	69.61	1	55	buccal muco	1	1	1	1	1	0	0	0	0000/00/00	0	3	0
1	26.20	0	54	buccal muco	1	1	1	4	2	2	1	0	2014/11/14	1	2	1
1	68.49	1	56	buccal muco	1	1	0	1	1	x	x	0	0000/00/00	0	1	
1	5.38	0	61	ant tongue	1	1	1	4	2	2	1	0	2014/2/14	1	2	1
1	52.79	0	43	buccal muco	1	0	1	3	1	1	0	0	0000/00/00	0	2	1
1	54.66	0	39	buccal muco	1	1	1	4	4	1	0	0	2016/7/4	1	2	1
1	68.59	1	67	buccal muco	1	1	1	4	4	0	0	0	0000/00/00	0	2	0
1	22.92	0	45	mouth floor	1	0	0	1	1	x	x	0	0000/00/00	0	3	
1	68.30	1	53	buccal muco	1	0	1	3	3	0	0	0	0000/00/00	0	2	0
1	68.26	1	44	buccal muco	0	1	1	1	1	0	0	0	0000/00/00	0	2	0
1	68.49	1	53	lip	1	0	0	2	2	0	0	0	0000/00/00	0	1	0
1	67.87	1	49	ant tongue	1	1	0	1	1	x	x	0	0000/00/00	0	2	
1	67.93	1	65	ant tongue	0	0	0	4	3	2	0	0	0000/00/00	0	2	1
1	9.41	0	47	ant tongue	0	1		4	1	2	1	0	2014/3/24	1	2	1
1	67.57	1	48	mouth floor	1	0	1	1	1	0	0	0	0000/00/00	0	2	0
1	67.74	1	53	ant tongue	1	1	1	4	2	2	0	0	0000/00/00	0	3	1
1	67.74	1	67	buccal muco	1	0	0	1	1	x	x	0	0000/00/00	0	1	
1	67.51	1	48	rmt	1	0	0	4	4	x	x	1	0000/00/00	0	2	
1	67.84	1	54	ant tongue	1	1	1	1	1	0	0	0	2014/5/23	1	2	0
1	67.51	1	52	ant tongue	1	0		4	2	2	0	0	0000/00/00	0	2	1
1	15.93	0	62	lip	1	0	0	3	2	1	0	0	2014/8/20	1	2	1
1	34.62	0	43	lip	1	1		無法	1	x	x	0	0000/00/00	0	1	
1	67.18	1	41	ant tongue	1	1	0	1	1	0	0	0	0000/00/00	0	2	0
1	67.18	1	54	ant tongue	1	0	0	1	1	0	0	0	0000/00/00	0	1	0
1	67.31	1	44	lip	1	1	1	2	2	x	x	0	0000/00/00	0	2	
1	67.97	1	36	ant tongue	0	0	0	1	1	x	x	0	0000/00/00	0	2	
1	67.31	1	68	buccal muco	1	1		1	1	x	x	0	0000/00/00	0	1	
1	67.11	1	58	ant tongue	0	1	0	2	2	0	0	0	0000/00/00	0	1	0
1	67.05	1	62	lip	1	0		4	4	0	0	0	0000/00/00	0	1	0
1	3.28	0	80	buccal muco	0	0	0	1	1	0	0	0	0000/00/00	0	2	0
1	41.93	0	58	buccal muco	1	1	1	4	4	2	1	0	0000/00/00	0	2	1
1	12.82	0	50	buccal muco	1	0	0	4	3	2	1	0	2014/6/5	1	2	1
1	67.11	1	62	buccal muco	1	1	0	1	1	0	0	0	0000/00/00	0	1	0
1	67.08	1	64	buccal muco	1	0	0	1	1	x	x	0	0000/00/00	0	1	
1	33.41	0	52	mouth floor	1	0	1	1	1	0	0	0	2014/8/1	1	2	0
1	3.90	0	76	alveolar ridge	0	0	0	4	4	0	0	0	2013/11/26	1	2	0
1	41.51	0	80	hard palate	0	0	0	2	2	x	x	0	2015/11/6	1	2	
1	19.44	0	54	buccal muco	0	0	1	1	1	0	0	0	2014/4/4	1	2	0
1	51.15	0	80	alveolar ridge	0	0	0	4	4	0	0	0	2017/10/24	1	2	0
1	66.85	1	83	buccal muco	0	0	0	3	3	0	0	0	0000/00/00	0	2	0
1	66.82	1	59	buccal muco	0	1	1	1	1	0	0	0	0000/00/00	0	1	0
1	66.85	1	48	buccal muco	0	0	1	2	2	0	0	0	2016/9/26	1	2	0
1	66.72	1	66	rmt	1	0	1	4	4	0	0	0	0000/00/00	0	2	0
1	27.25	0	68	ant tongue	1	1	1	1	1	0	0	0	0000/00/00	0	2	0

1	3.90	0	72	alveolar ridg	1	1		4	4	0	0	0	0000/00/00	0	1	0
1	18.82	0	56	ant tongue	0	0	0	無法	1	x	x	0	0000/00/00	0	2	
1	20.49	0	72	buccal muco	0	0	0	2	2	x	x	0	2014/2/14	1	2	
1	11.67	0	56	lip	0	0	0	4	4	1	1	0	2014/9/22	1	2	1
1	14.16	0	45	ant tongue	1	0	1	4	4	0	0	0	2014/7/23	1	2	0
1	66.43	1	53	buccal muco	1	1	0	1	1	x	x	0	0000/00/00	0	2	
1	14.62	0	62	buccal muco	0	0	0	2	2	0	0	0	2014/3/11	1	2	0
1	66.26	1	54	lip	1	1	1	2	2	0	0	0	0000/00/00	0	1	0
1	66.23	1	44	mouth floor	1	1	1	1	1	0	0	0	0000/00/00	0	2	0
1	65.21	0	56	buccal muco	1	1	0	4	4	0	0	0	0000/00/00	0	2	0
1	45.02	0	65	lip	1	1	1	4	4	0	0	0	0000/00/00	0	2	0
1	66.03	1	53	buccal muco	1	0	1	1	1	0	0	0	0000/00/00	0	2	0
1	65.97	1	77	ant tongue	0	0	1	1	1	0	0	0	2016/3/22	1	2	0
1	66.66	1	45	buccal muco	1	0	0	3	1	1	0	0	0000/00/00	0	2	1
1	66.43	1	39	buccal muco	1	1		4	4	0	0	0	0000/00/00	0	2	0
1	65.97	1	59	ant tongue	1	1	1	2	2	0	0	0	0000/00/00	0	2	0
1	67.08	1	70	buccal muco	1	1	0	1	1	x	x	0	2014/12/19	1	2	
1	10.20	0	57	hard palate	1	1	0	4	2	2	1	0	0000/00/00	0	2	1
1	78.66	1	62	ant tongue	1	0	0	1	1	0	0	0	2014/2/24	1	1	0
1	25.18	0	45	buccal muco	1	0	1	4	4	2	1	0	2014/12/26	1	2	1
1	14.26	0	56	buccal muco	1	0	0	2	2	0	0	0	2014/9/25	1	2	0
1	65.93	1	70	buccal muco	0	0	1	1	1	x	x	0	2013/12/13	1	1	
1	7.28	0	69	rmt	1	0	1	4	4	2	1	0	2014/5/8	1	2	1
1	65.54	1	45	lip	1	1	0	2	2	0	0	0	0000/00/00	0	2	0
1	65.54	1	58	buccal muco	0	1	0	1	1	x	x	0	0000/00/00	0	2	
1	65.51	1	59	ant tongue	0	0	0	1	1	0	0	0	0000/00/00	0	1	0
1	66.00	1	41	ant tongue	1	0	1	1	1	0	0	0	2016/11/4	1	1	0
1	51.67	0	63	lip	0	0	0	1	1	x	x	0	2014/5/10	1	2	
1	67.77	1	55	rmt	1	0	0	4	4	1	0	0	0000/00/00	0	2	1
1	65.41	1	63	buccal muco	1	0	1	無法	1	x	x	0	2018/3/23	1	2	
1	65.28	1	63	alveolar ridg	1	1	0	4	4	0	0	0	0000/00/00	0	2	0
1	65.77	1	64	buccal muco	0	0	0	1	1	x	x	0	0000/00/00	0	2	
1	9.38	0	44	buccal muco	0	1		3	3	0	0	0	2014/6/24	1	2	0
1	65.05	1	58	rmt	0	0	0	4	4	0	0	0	0000/00/00	0	1	0
1	66.39	1	52	buccal muco	1	0	1	3	2	1	1	0	0000/00/00	0	2	1
1	64.98	1	43	buccal muco	1	1	0	2	2	0	0	0	2016/6/17	1	2	0
1	33.64	0	51	buccal muco	1	0		2	2	0	0	0	0000/00/00	0	1	0
1	65.38	1	37	ant tongue	1	1		1	1	0	0	0	2016/1/6	1	2	0
1	8.95	0	58	mouth floor	0	0	0	1	1	0	0	0	0000/00/00	0	2	0
1	64.89	1	63	buccal muco	1	0	0	1	1	0	0	0	0000/00/00	0	2	0
1	5.51	0	47	alveolar ridg	1	1	1	4	4	2	1	0	2014/4/18	1	2	1
1	9.15	0	39	buccal muco	1	1	1	4	3	2	0	0	2014/9/17	1	2	1
1	65.08	1	49	lip	0	0	0	1	1	x	x	0	0000/00/00	0	1	
1	65.18	1	50	mouth floor	1	1		4	1	2	0	0	0000/00/00	0	2	1
1	9.80	0	50	ant tongue	1	0	0	4	2	2	1	0	0000/00/00	0	2	1
1	56.26	0	64	alveolar ridg	0	0	0	1	1	x	x	0	0000/00/00	0	2	
1	64.52	1	63	alveolar ridg	0	0	0	4	4	0	0	0	0000/00/00	0	2	0
1	64.39	1	68	buccal muco	0	0	0	2	2	0	0	0	0000/00/00	0	2	0
1	64.49	1	69	buccal muco	1	0		2	2	x	x	0	0000/00/00	0	1	
1	65.11	1	48	ant tongue	0	0	1	1	1	x	x	0	0000/00/00	0	1	
1	64.79	1	48	buccal muco	1	1	1	4	4	0	0	0	0000/00/00	0	2	0
1	24.33	0	58	ant tongue	1	1	1	1	1	0	0	0	0000/00/00	0	2	0
1	64.98	1	58	buccal muco	0	0	0	1	1	0	0	0	0000/00/00	0	1	0
1	26.43	0	60	ant tongue	1	0	1	2	2	x	x	0	0000/00/00	0	2	
1	17.31	0	51	lip	0	0	0	4	4	0	0	0	2014/5/29	1	3	0
1	64.36	1	54	buccal muco	1	1	0	1	1	0	0	0	0000/00/00	0	1	0
1	64.10	1	54	buccal muco	1	0	0	2	2	0	0	0	0000/00/00	0	3	0
1	22.85	0	55	lip	0	0	0	1	1	x	x	0	2015/8/30	1	2	
1	64.07	1	47	ant tongue	0	0	0	1	1	x	x	0	0000/00/00	0	2	
1	64.20	1	50	alveolar ridg	1	0	0	4	4	0	0	0	0000/00/00	0	2	0
1	63.70	1	57	lip	0	1	0	1	1	0	0	0	0000/00/00	0	2	0
1	64.39	1	54	hard palate	1	0	1	1	1	x	x	0	0000/00/00	0	1	
1	63.64	1	46	buccal muco	1	1	1	3	3	0	0	0	0000/00/00	0	2	0
1	63.44	1	61	lip	0	0	0	4	4	0	0	0	0000/00/00	0	3	0
1	25.31	0	57	ant tongue	1	0	1	4	4	2	1	0	2014/10/31	1	2	1
1	10.92	0	58	buccal muco	1	1		4	2	2	0	0	0000/00/00	0	2	1
1	63.21	1	56	lip	0	0	0	1	1	x	x	0	0000/00/00	0	2	
1	63.18	1	45	buccal muco	0	1	0	1	1	0	0	0	0000/00/00	0	2	0
1	63.05	1	46	lip	1	0	0	3	1	1	0	0	0000/00/00	0	2	1
1	64.75	1	47	buccal muco	1	1	1	1	1	x	x	0	0000/00/00	0	1	
1	63.05	1	54	alveolar ridg	1	1	1	4	4	0	0	0	0000/00/00	0	1	0
1	37.54	0	66	buccal muco	1	0	1	3	3	0	0	0	0000/00/00	0	1	0

1	63.02	1	38	lip	1	1		2	2	0	0	0	0000/00/00	0	2	0
1	62.98	1	56	buccal muco	1	1	0	4	4	0	0	0	0000/00/00	0	2	0
1	62.98	1	51	buccal muco	0	0	0	4	2	2	1	0	0000/00/00	0	2	1
1	54.52	0	56	lip	0	0	0	1	1	x	x	0	0000/00/00	0	2	
1	64.43	1	71	buccal muco	0	0	0	1	1	x	x	0	0000/00/00	0	2	
1	26.30	0	58	alveolar ridge	1	1	1	4	4	0	0	0	2015/5/6	1	2	0
1	62.69	1	57	buccal muco	1	1	1	4	4	1	0	0	0000/00/00	0	2	1
1	62.59	1	67	buccal muco	1	1	1	3	1	1	0	0	0000/00/00	0	2	1
1	62.69	1	75	buccal muco	0	0	0	1	1	0	0	0	0000/00/00	0	2	0
1	11.84	0	70	buccal muco	0	0	0	4	1	0	0	0	0000/00/00	0	2	0
1	62.75	1	38	ant tongue	1	1	0	4	4	2	1	0	0000/00/00	0	2	1
1	64.98	1	45	buccal muco	1	1	0	1	1	0	0	0	0000/00/00	0	2	0
1	46.46	0	71	buccal muco	0	0	0	1	1	x	x	0	0000/00/00	0	2	
1	10.20	0	74	alveolar ridge	1	1	0	1	1	0	0	0	0000/00/00	0	2	0
1	8.10	0	48	buccal muco	0	0	0	4	4	0	0	0	2014/9/24	1	2	0
1	0.82	0	55	buccal muco	1	0	0	1	1	0	0	0	0000/00/00	0	1	0
1	4.16	0	70	buccal muco	0	0	0	4	3	2	1	0	2014/5/14	1	2	1
1	62.13	1	44	ant tongue	1	0	0	1	1	0	0	0	0000/00/00	0	1	0
1	62.13	1	48	ant tongue	1	0	0	1	1	0	0	0	0000/00/00	0	2	0
1	62.13	1	64	lip	1	1	0	3	3	0	0	0	0000/00/00	0	2	0
1	25.41	0	48	rmt	0	0	0	1	1	0	0	0	0000/00/00	0	1	0
1	15.51	0	54	buccal muco	1	1	0	4	4	2	1	0	0000/00/00	0	2	1
1	10.46	0	53	hard palate	1	1		4	4	2	1	0	0000/00/00	0	2	1
1	61.77	1	57	alveolar ridge	0	0	0	1	1	x	x	0	0000/00/00	0	2	
1	61.77	1	60	ant tongue	0	0	0	1	1	0	0	0	0000/00/00	0	2	0
1	61.67	1	50	alveolar ridge	0	0	0	4	4	0	0	0	2018/5/14	1	1	0
1	62.36	1	36	rmt	1	1	1	2	2	0	0	0	0000/00/00	0	2	0
1	10.13	0	51	buccal muco	0	0	0	4	4	2	1	0	0000/00/00	0	1	1
1	61.84	1	42	buccal muco	0	0	0	1	1	0	0	0	0000/00/00	0	1	0
1	52.46	0	56	buccal muco	1	0	0	1	1	0	0	0	2017/2/27	1	2	0
1	15.70	0	70	alveolar ridge	0	0	0	1	1	x	x	0	0000/00/00	0	2	
1	61.41	1	57	buccal muco	0	0	0	1	1	0	0	0	0000/00/00	0	2	0
1	61.80	1	46	buccal muco	1	0	1	3	3	0	0	0	0000/00/00	0	1	0
1	3.97	0	46	lip	1	0	0	2	2	x	x	1	0000/00/00	0	2	
1	61.31	1	43	buccal muco	1	1	0	2	2	0	0	0	0000/00/00	0	2	0
1	25.41	0	48	hard palate	0	0	0	2	2	0	0	1	0000/00/00	0	2	0
1	6.89	0	53	buccal muco	1	0	0	3	2	1	0	0	2014/9/10	1	2	1
1	61.44	1	61	alveolar ridge	1	0	0	4	4	2	1	0	0000/00/00	0	2	1
1	61.67	1	43	mouth floor	1	0	0	1	1	0	0	0	0000/00/00	0	2	0
1	0.59	0	58	buccal muco	1	0		3	3	0	0	0	0000/00/00	0	2	0
1	61.21	1	56	alveolar ridge	1	0	0	4	4	0	0	0	0000/00/00	0	2	0
1	7.08	0	76	buccal muco	1	1	0	4	4	0	0	0	0000/00/00	0	2	0
1	61.57	1	45	ant tongue	1	0		4	4	0	0	0	0000/00/00	0	2	0
1	4.23	0	39	ant tongue	1	1		4	4	3	1	0	0000/00/00	0	3	1
1	61.41	1	34	buccal muco	1	1		2	2	0	0	0	0000/00/00	0	1	0
1	60.85	1	54	hard palate	1	0	0	1	1	x	x	0	0000/00/00	0	2	
1	60.62	1	58	alveolar ridge	1	0	0	1	1	0	0	0	0000/00/00	0	2	0
1	8.82	0	49	ant tongue	1	0	0	1	1	0	0	0	2014/6/19	1	2	0
1	60.69	1	57	alveolar ridge	1	0	0	4	4	0	0	0	0000/00/00	0	2	0
1	39.90	0	62	hard palate	0	0	0	1	1	0	0	0	0000/00/00	0	2	0
1	60.89	1	48	ant tongue	1	1	1	1	1	0	0	0	0000/00/00	0	1	0
1	16.56	0	44	alveolar ridge	1	1		4	4	0	0	0	2015/5/21	1	2	0
1	60.46	1	53	ant tongue	1	0		2	2	0	0	0	0000/00/00	0	2	0
1	60.30	1	66	buccal muco	0	0		1	1	0	0	0	0000/00/00	0	2	0
1	60.39	1	57	ant tongue	0	0	0	4	4	0	0	1	0000/00/00	0	2	0
1	10.20	0	61	buccal muco	0	0	0	2	2	0	0	0	0000/00/00	0	2	0
1	60.66	1	50	ant tongue	1	0	1	1	1	x	x	0	0000/00/00	0	2	
1	6.75	0	51	hard palate	0	0	0	3	3	1	0	1	0000/00/00	0	3	1
1	54.98	0	58	buccal muco	0	1		4	4	2	1	1	0000/00/00	0	2	1
1	60.23	1	60	alveolar ridge	1	1		4	4	0	0	0	2017/1/14	1	2	0
1	60.03	1	57	alveolar ridge	1	0	0	2	2	0	0	0	0000/00/00	0	2	0
1	19.74	0	60	rmt	1	1	0	4	4	0	0	0	2015/5/27	1	2	0
1	60.39	1	40	buccal muco	1	0	0	1	1	x	x	0	0000/00/00	0	1	
1	19.05	0	53	alveolar ridge	0	0	0	1	1	0	0	0	0000/00/00	0	1	0
1	3.84	0	64	alveolar ridge	0	0	0	無法	4	x	x	0	0000/00/00	0	2	
1	59.93	1	43	lip	1	1	1	1	1	0	0	0	0000/00/00	0	2	0
1	59.93	1	66	alveolar ridge	0	0	0	1	1	0	0	0	0000/00/00	0	1	0
1	12.79	0	61	buccal muco	0	0	0	2	2	0	0	0	0000/00/00	0	1	0
1	60.39	1	55	rmt	0	0	0	2	2	0	0	0	0000/00/00	0	2	0
1	59.80	1	44	buccal muco	0	0	0	1	1	0	0	0	0000/00/00	0	2	0
1	59.70	1	69	alveolar ridge	0	0	0	1	1	x	x	0	0000/00/00	0	2	
1	44.39	0	55	lip	1	1	0	1	1	0	0	0	0000/00/00	0	2	0

1	9.25	0	55	alveolar ridg	1	0	0	4	4	0	0	0	0000/00/00	0	2	0
1	4.66	0	46	lip	1	0	1	4	4	2	1	0	2014/10/6	1	2	1
1	59.51	1	41	buccal muco	1	1	0	1	1	x	x	0	0000/00/00	0	1	
1	59.28	1	79	ant tongue	0	0	0	2	2	0	0	0	0000/00/00	0	2	0
1	59.38	1	50	ant tongue	1	1	0	1	1	x	x	0	0000/00/00	0	1	
1	22.49	0	56	buccal muco	1	1	1	2	2	0	0	0	2015/5/21	1	2	0
1	59.48	1	47	lip	0	0	0	1	1	x	x	0	2016/6/27	1	2	
1	59.31	1	66	ant tongue	0	0	0	1	1	x	x	0	0000/00/00	0	2	
1	59.54	1	70	ant tongue	0	0		2	2	0	0	0	0000/00/00	0	2	0
1	59.21	1	56	buccal muco	0	0	1	2	2	0	0	0	0000/00/00	0	1	0
1	16.39	0	55	ant tongue	1	0	0	3	1	1	0	0	2014/11/18	1	2	1
1	31.18	0	68	alveolar ridg	1	0	0	4	4	0	0	0	0000/00/00	0	1	0
1	59.11	1	61	alveolar ridg	0	0	1	3	3	0	0	0	0000/00/00	0	2	0
1	59.11	1	67	ant tongue	1	0	1	2	2	0	0	0	0000/00/00	0	1	0
1	59.77	1	41	buccal muco	0	0	0	2	2	0	0	0	0000/00/00	0	2	0
1	59.44	1	54	buccal muco	0	0	0	4	4	0	0	0	0000/00/00	0	2	0
1	40.10	0	53	buccal muco	1	0	1	2	2	0	0	0	0000/00/00	0	2	0
1	59.08	1	78	lip	0	0	0	1	1	x	x	0	0000/00/00	0	2	
1	6.07	0	46	rmt	1	0	0	4	4	2	1	0	2014/12/24	1	2	1
1	32.49	0	45	ant tongue	1	1		4	1	2	1	0	2015/8/27	1	2	1
1	58.85	1	61	rmt	1	0	1	1	1	0	0	0	0000/00/00	0	2	0
1	36.13	0	56	ant tongue	1	1	1	4	4	1	1	0	2016/8/9	1	2	1
1	60.00	1	52	buccal muco	0	0	0	1	1	0	0	0	0000/00/00	0	1	0
1	58.79	1	67	ant tongue	1	1	1	1	1	0	0	0	0000/00/00	0	2	0
1	58.79	1	55	ant tongue	1	1	1	2	2	0	0	0	0000/00/00	0	3	0
1	58.66	1	70	buccal muco	0	0	0	1	1	0	0	0	0000/00/00	0	2	0
1	58.56	1	54	buccal muco	1	0	1	1	1	0	0	0	0000/00/00	0	2	0
1	58.82	1	58	buccal muco	1	1	1	1	1	0	0	0	0000/00/00	0	1	0
1	19.31	0	90	buccal muco	1	0	0	2	2	0	0	0	0000/00/00	0	1	0
1	6.03	0	58	lip	1	1		1	1	x	x	0	0000/00/00	0	1	
1	58.56	1	62	buccal muco	0	0	0	3	3	0	0	0	0000/00/00	0	2	0
1	58.23	1	77	buccal muco	0	0	0	1	1	x	x	0	0000/00/00	0	2	
1	12.85	0	59	ant tongue	0	0	0	1	1	x	x	0	2014/9/15	1	3	
1	9.38	0	63	ant tongue	1	0	1	4	4	2	1	0	2015/2/10	1	2	1
1	20.39	0	62	ant tongue	1	1	1	4	2	2	0	0	2015/8/17	1	2	1
1	58.20	1	59	buccal muco	1	0	1	3	2	1	0	0	0000/00/00	0	2	1
1	46.75	0	53	buccal muco	0	0	0	1	1	x	x	0	0000/00/00	0	2	
1	5.05	0	57	hard palate	1	0	1	4	4	0	0	1	0000/00/00	0	2	0
1	58.16	1	58	ant tongue	1	0	1	1	1	x	x	0	0000/00/00	0	2	
1	5.38	0	52	buccal muco	0	0	0	4	4	2	1	0	0000/00/00	0	2	1
1	60.92	1	45	ant tongue	1	1	1	2	2	0	0	0	0000/00/00	0	2	0
1	58.30	1	43	ant tongue	1	0	1	1	1	0	0	0	0000/00/00	0	2	0
1	58.85	1	57	buccal muco	1	0	1	3	3	0	0	0	0000/00/00	0	1	0
1	12.07	0	51	alveolar ridg	1	0		4	4	0	0	0	0000/00/00	0	2	0
1	6.46	0	56	ant tongue	1	0	1	1	1	0	0	0	2014/11/26	1	2	0
1	57.84	1	43	buccal muco	0	0	0	1	1	x	x	0	0000/00/00	0	2	
1	24.85	0	44	buccal muco	0	0	0	1	1	x	x	0	2014/11/14	1	1	
1	58.62	1	57	buccal muco	1	1	1	1	1	0	0	0	0000/00/00	0	2	0
1	66.66	1	62	ant tongue	1	1	0	2	2	0	0	0	2014/8/11	1	1	0
1	57.64	1	59	alveolar ridg	1	1	1	4	4	0	0	0	0000/00/00	0	2	0
1	58.20	1	66	buccal muco	0	0	0	2	2	x	x	1	2015/5/7	1	1	
1	57.54	1	56	alveolar ridg	1	0	0	4	4	0	0	0	0000/00/00	0	2	0
1	57.54	1	57	ant tongue	0	0	0	1	1	0	0	0	0000/00/00	0	2	0
1	57.41	1	46	ant tongue	1	1	0	1	1	x	x	0	0000/00/00	0	2	
1	57.31	1	64	ant tongue	1	1	1	4	2	2	1	0	0000/00/00	0	2	1
1	57.93	1	69	lip	1	0		1	1	0	0	0	0000/00/00	0	2	0
1	57.25	1	36	ant tongue	1	1	1	1	1	0	0	0	0000/00/00	0	2	0
1	57.25	1	57	alveolar ridg	1	0	0	1	1	x	x	0	0000/00/00	0	2	
1	57.25	1	44	mouth floor	1	1	1	1	1	x	x	0	0000/00/00	0	2	
1	59.57	1	45	ant tongue	1	1	1	1	1	x	x	0	2017/7/8	1	2	
1	3.54	0	50	lip	1	0	0	4	3	2	1	0	2014/9/24	1	2	1
1	1.87	0	56	ant tongue	1	1		4	4	2	1	0	2014/10/2	1	2	1
1	59.34	1	53	alveolar ridg	1	0	1	4	4	0	0	0	0000/00/00	0	1	0
1	57.48	1	78	lip	0	0	0	1	1	x	x	0	0000/00/00	0	1	
1	56.98	1	75	buccal muco	1	0	0	1	1	0	0	0	0000/00/00	0	2	0
1	56.98	1	52	ant tongue	1	1	0	無法		0	x	0	0000/00/00	0	2	0
1	57.31	1	33	rmt	1	0		1	1	0	0	0	0000/00/00	0	2	0
1	13.67	0	63	ant tongue	1	1	0	2	2	0	0	0	2015/4/23	1	3	0
1	57.21	1	52	buccal muco	0	0	0	1	1	0	0	0	0000/00/00	0	1	0
1	58.10	1	72	buccal muco	1	1	0	1	1	0	0	0	0000/00/00	0	1	0
1	56.75	1	71	ant tongue	1	1		1	1	0	0	0	0000/00/00	0	1	0
1	56.92	1	60	ant tongue	1	1	1	2	2	x	x	0	2014/12/27	1	2	

1	25.31	0	64	lip	1	1	0	1	1	x	x	0	0000/00/00	0	1	
1	63.11	1	59	ant tongue	1	1		1	1	x	x	0	0000/00/00	0	2	
1	56.79	1	56	buccal muco	1	1		2	2	0	0	0	0000/00/00	0	1	0
1	56.75	1	45	buccal muco	1	0	0	2	2	0	0	1	2014/12/26	1	2	0
1	56.72	1	55	buccal muco	0	0	0	1	1	0	0	0	0000/00/00	0	2	0
1	56.59	1	79	rmt	1	0	0	1	1	x	x	0	0000/00/00	0	2	
1	57.54	1	67	buccal muco	0	0	1	2	2	0	0	0	0000/00/00	0	2	0
1	15.34	0	47	rmt	1	0	0	4	4	x	x	0	2015/1/20	1	2	
1	56.56	1	64	buccal muco	1	1	0	3	3	0	0	0	0000/00/00	0	2	0
1	56.59	1	47	buccal muco	1	1	0	1	1	0	0	0	0000/00/00	0	1	0
1	56.82	1	41	buccal muco	1	1	1	3	3	1	0	0	0000/00/00	0	2	1
1	47.77	0	50	buccal muco	1	1	1	2	2	0	0	0	0000/00/00	0	2	0
1	56.52	1	62	hard palate	1	1	1	1	1	x	x	0	0000/00/00	0	2	
1	15.70	0	70	hard palate	1	1	1	4	4	0	0	0	2015/4/9	1	2	0
1	56.39	1	41	mouth floor	1	0	1	4	4	2	0	0	0000/00/00	0	2	1
1	56.52	1	69	buccal muco	0	0	0	3	3	0	0	0	0000/00/00	0	2	0
1	56.33	1	54	mouth floor	0	0	0	1	1	0	0	0	0000/00/00	0	1	0
1	56.33	1	75	buccal muco	0	0	0	1	1	0	0	0	0000/00/00	0	1	0
1	56.46	1	45	ant tongue	0	1	1	1	1	x	x	0	0000/00/00	0	1	
1	7.41	0	58	ant tongue	1	0	0	4	2	2	1	0	2015/3/27	1	2	1
1	56.36	1	55	buccal muco	1	0	0	1	1	0	0	0	0000/00/00	0	2	0
1	56.30	1	45	buccal muco	1	1	1	3	3	0	0	0	0000/00/00	0	2	0
1	56.26	1	41	buccal muco	0	0	0	1	1	x	x	0	0000/00/00	0	2	
1	56.16	1	55	buccal muco	1	0	1	4	4	0	0	0	0000/00/00	0	2	0
1	56.13	1	55	hard palate	0	0	0	2	2	0	0	0	0000/00/00	0	2	0
1	5.25	0	49	hard palate	1	1		4	4	1	1	0	0000/00/00	0	2	1
1	47.28	0	71	mouth floor	1	1	1	2	2	0	0	0	0000/00/00	0	3	0
1	56.36	1	70	ant tongue	1	0	1	2	2	0	0	0	0000/00/00	0	2	0
1	56.23	1	49	rmt	0	0	0	1	1	0	0	1	2015/1/3	1	1	0
1	56.10	1	58	hard palate	1	0	1	1	1	0	0	0	0000/00/00	0	2	0
1	56.13	1	47	buccal muco	1	0	1	1	1	0	0	0	0000/00/00	0	2	0
1	17.21	0	43	ant tongue	1	1	1	3	3	0	0	0	2015/7/6	1	2	0
1	44.00	0	42	lip	1	0	1	1	1	0	0	1	0000/00/00	0	2	0
1	59.15	1	50	alveolar ridg	0	0	0	1	1	0	0	0	0000/00/00	0	1	0
1	56.13	1	52	ant tongue	1	0	1	1	1	x	x	0	0000/00/00	0	2	
1	57.38	1	47	alveolar ridg	0	1	0	4	4	0	0	0	0000/00/00	0	2	0
1	48.92	0	56	alveolar ridg	0	0	0	3	1	1	0	1	2014/10/1	1	2	1
1	5.61	0	58	alveolar ridg	1	1		4	4	x	x	0	2014/10/2	1	2	
1	55.80	1	53	ant tongue	1	1		2	2	0	0	0	0000/00/00	0	2	0
1	55.70	1	76	buccal muco	0	1	0	1	1	x	x	0	0000/00/00	0	1	
1	55.80	1	43	ant tongue	1	1	1	4	4	1	0	0	0000/00/00	0	2	1
1	56.39	1	60	buccal muco	0	0		1	1	0	0	0	0000/00/00	0	2	0
1	10.26	0	53	buccal muco	0	0		2	2	0	0	1	0000/00/00	0	3	0
1	56.10	1	61	buccal muco	0	0	0	4	4	0	0	0	0000/00/00	0	2	0
1	18.03	0	36	ant tongue	1	0	1	4	4	1	1	0	0000/00/00	0	2	1
1	55.54	1	48	ant tongue	1	0		2	2	0	0	0	0000/00/00	0	2	0
1	57.28	1	54	alveolar ridg	1	0	1	1	1	0	0	0	0000/00/00	0	1	0
1	55.44	1	39	buccal muco	1	1	1	3	2	1	0	0	0000/00/00	0	2	1
1	55.48	1	76	alveolar ridg	0	1	1	4	4	0	0	0	0000/00/00	0	2	0
1	56.26	1	72	buccal muco	0	0	0	1	1	x	x	0	0000/00/00	0	1	
1	55.41	1	56	hard palate	1	1		1	1	0	0	0	0000/00/00	0	1	0
1	55.34	1	57	hard palate	1	1	0	1	1	x	x	0	0000/00/00	0	2	
1	37.44	0	61	ant tongue	1	0	1	1	1	0	0	0	0000/00/00	0	3	0
1	55.25	1	65	buccal muco	1	0	1	1	1	0	0	0	0000/00/00	0	1	0
1	55.41	1	55	ant tongue	1	1	1	3	2	1	0	0	0000/00/00	0	2	1
1	55.34	1	63	buccal muco	1	1	0	2	2	x	x	0	2015/11/5	1	2	
1	24.89	0	58	alveolar ridg	1	1	0	4	4	0	0	0	0000/00/00	0	2	0
1	55.41	1	36	buccal muco	1	1	1	1	1	x	x	0	0000/00/00	0	2	
1	3.31	0	58	rmt	1	1	1	1	1	0	0	0	0000/00/00	0	2	0
1	43.11	0	42	lip	1	0	1	1	1	0	0	1	0000/00/00	0	2	0
1	54.98	1	50	lip	0	0	0	3	2	1	0	0	0000/00/00	0	2	1
1	55.80	1	79	ant tongue	0	0	1	2	2	x	x	0	0000/00/00	0	2	
1	43.11	0	42	buccal muco	1	0	1	1	1	0	0	0	0000/00/00	0	1	0
1	38.95	0	53	buccal muco	1	1	1	2	2	0	0	0	0000/00/00	0	2	0
1	25.84	0	63	buccal muco	1	0	0	1	1	0	0	0	0000/00/00	0	2	0
1	55.38	1	76	alveolar ridg	0	1	0	1	1	x	x	0	0000/00/00	0	1	
1	54.95	1	48	ant tongue	1	1		1	1	0	0	0	0000/00/00	0	1	0
1	55.34	1	60	buccal muco	1	0	0	1	1	x	x	0	0000/00/00	0	2	
1	54.72	1	54	ant tongue	1	0		1	1	x	x	0	2015/9/18	1	2	
1	55.18	1	44	buccal muco	1	0	0	2	2	0	0	0	0000/00/00	0	2	0
1	54.66	1	65	buccal muco	1	0	1	1	1	0	0	0	0000/00/00	0	1	0
1	52.98	0	76	alveolar ridg	1	1	0	2	2	x	x	0	0000/00/00	0	2	

1	54.56	1	77	alveolar ridg	0	0	0		1	1	x	x		0	0000/00/00	0	1	
1	54.56	1	65	buccal muco	1	1	1		2	2		0	0	0	0000/00/00	0	3	0
1	54.56	1	56	alveolar ridg	1	1	1		4	4		0	0	0	0000/00/00	0	1	0
1	54.30	1	62	buccal muco	0	0	1		1	1		0	0	0	0000/00/00	0	2	0
1	54.33	1	66	alveolar ridg	1	1	1		2	2		0	0	0	0000/00/00	0	2	0
1	54.43	1	74	ant tongue	1	0	1		1	1		0	0	0	0000/00/00	0	2	0
1	54.49	1	62	buccal muco	1	1	0		2	2		0	0	0	0000/00/00	0	1	0
1	54.23	1	52	buccal muco	1	1	1		1	1		0	0	0	0000/00/00	0	2	0
1	12.43	0	77	alveolar ridg	1	1	0		4	4		0	0	0	0000/00/00	0	2	0
1	28.00	0	64	buccal muco	1	1	1		1	1	x	x		0	2015/3/12	1	2	
1	41.02	0	61	hard palate	0	0	0		1	1	x	x		0	0000/00/00	0	2	
1	54.66	1	60	alveolar ridg	1	0	0		1	1	x	x		0	0000/00/00	0	1	
1	54.75	1	50	alveolar ridg	1	1	1		4	2		2	1	0	0000/00/00	0	2	1
1	30.75	0	57	lip	1	1	0		4	2		2	0	0	0000/00/00	0	2	1
1	54.79	1	40	mouth floor	1	0	1		1	1		0	0	0	0000/00/00	0	2	0
1	54.10	1	73	ant tongue	1	0	1		3	3		0	0	0	0000/00/00	0	1	0
1	13.28	0	60	alveolar ridg	1	0	0		4	2		2	1	0	2015/5/20	1	1	1
1	54.56	1	49	ant tongue	1	1			2	2		0	0	0	0000/00/00	0	2	0
1	13.44	0	67	alveolar ridg	1	1	1		1	1	x	x		0	0000/00/00	0	2	
1	53.93	1	42	alveolar ridg	1	1	0	無法		1	x	x		0	0000/00/00	0	1	
1	67.64	1	67	alveolar ridg	1	0	0		1	1	x	x		0	0000/00/00	0	1	
1	14.66	0	50	buccal muco	1	0	0		1	1	x	x		0	0000/00/00	0	2	
1	30.23	0	66	lip	0	0	0		1	1	x	x		0	0000/00/00	0	2	
1	45.18	0	45	ant tongue	0	0	0		2	2		0	0	0	2017/7/25	1	2	0
1	53.64	1	72	lip	0	0	0		2	2		0	0	0	0000/00/00	0	2	0
1	53.64	1	44	buccal muco	0	0	0		1	1		0	0	0	0000/00/00	0	2	0
1	13.48	0	50	ant tongue	1	1			4	4		2	1	0	2015/9/24	1	3	1
1	53.57	1	35	ant tongue	1	1	0		1	1		0	0	0	0000/00/00	0	2	0
1	53.57	1	61	buccal muco	1	1			2	2	x	x		0	0000/00/00	0	2	
1	53.57	1	78	buccal muco	0	0	1		2	2		0	0	0	0000/00/00	0	2	0
1	53.57	1	81	buccal muco	0	0	0		1	1		0	0	0	0000/00/00	0	1	0
1	53.51	1	73	alveolar ridg	0	0	0		4	4		0	0	0	0000/00/00	0	2	0
1	11.54	0	68	alveolar ridg	1	1	0		4	4		0	0	0	0000/00/00	0	2	0
1	53.48	1	52	ant tongue	0	1	1		4	4		0	0	0	0000/00/00	0	2	0
1	53.41	1	59	mouth floor	1	0	0		2	2		0	0	0	0000/00/00	0	2	0
1	53.38	1	45	ant tongue	1	0	0		4	4		0	0	0	0000/00/00	0	2	0
1	55.70	1	63	ant tongue	0	0	0		1	1	x	x		0	0000/00/00	0	1	
1	53.28	1	63	alveolar ridg	1	0	1		1	1	x	x		0	0000/00/00	0	2	
1	53.28	1	62	ant tongue	1	1	1		4	4		0	0	0	0000/00/00	0	2	0
1	75.11	1	61	alveolar ridg	1	0	0		1	1		0	0	1	0000/00/00	0	2	0
1	74.39	1	50	buccal muco	1	1	1		1	1		0	0	0	0000/00/00	0	2	0
1	6.30	0	67	hard palate	1	1	0	無法		1	x	x		1	0000/00/00	0	2	
1	53.57	1	54	buccal muco	1	1	1		1	1	x	x		0	0000/00/00	0	2	
1	29.97	0	57	alveolar ridg	1	1	0		4	4		2	0	0	0000/00/00	0	2	1
1	35.97	0	59	ant tongue	0	1			1	1		0	0	0	2015/10/21	1	2	0
1	49.28	0	60	alveolar ridg	1	0	1		4	4		0	0	0	0000/00/00	0	2	0
1	52.89	1	65	lip	0	0	0		1	1		0	0	0	0000/00/00	0	2	0
1	10.26	0	39	ant tongue	0	0	0		1	1		0	0	0	0000/00/00	0	2	0
1	46.79	0	55	buccal muco	1	0			3	3		0	0	0	0000/00/00	0	3	0
1	13.74	0	53	alveolar ridg	0	0	0		1	1	x	x		0	2015/3/27	1	2	
1	52.69	1	51	mouth floor	1	0	1		1	1	x	x		0	0000/00/00	0	2	
1	26.07	0	41	mouth floor	1	0			4	1		2	1	1	0000/00/00	0	3	1
1	52.66	1	66	buccal muco	0	0	0		1	1		0	0	0	0000/00/00	0	1	0
1	52.49	1	57	ant tongue	1	0	0		1	1		0	0	0	0000/00/00	0	3	0
1	23.31	0	82	rmt	0	0	0		1	1	x	x		0	0000/00/00	0	2	
1	52.26	1	58	buccal muco	0	0	0		4	2		2	1	0	0000/00/00	0	2	1
1	53.28	1	71	hard palate	0	0	0		3	3		0	0	0	0000/00/00	0	3	0
1	52.23	1	68	alveolar ridg	0	0	1		4	4		0	0	0	0000/00/00	0	1	0
1	52.13	1	54	buccal muco	1	1			1	1		0	0	0	0000/00/00	0	2	0
1	34.62	0	63	lip	1	0	0		1	1		0	0	0	0000/00/00	0	2	0
1	52.13	1	54	lip	1	1	1		2	2		0	0	0	0000/00/00	0	2	0
1	13.25	0	75	buccal muco	0	0	0		1	1	x	x		0	0000/00/00	0	2	
1	52.13	1	43	ant tongue	1	1	1		4	2		2	1	0	0000/00/00	0	2	1
1	52.03	1	63	alveolar ridg	0	0	0		4	4		0	0	0	2018/5/28	1	1	0
1	7.87	0	65	ant tongue	0	0	0		4	3		2	1	0	2015/6/17	1	2	1
1	51.97	1	45	buccal muco	1	1	1		1	1		0	0	0	0000/00/00	0	2	0
1	51.97	1	70	alveolar ridg	0	0	0		1	1	x	x		0	0000/00/00	0	2	
1	52.26	1	60	buccal muco	1	0	0		2	2		0	0	0	0000/00/00	0	2	0
1	52.39	1	72	ant tongue	0	0	0		1	1	x	x		0	0000/00/00	0	2	
1	51.80	1	55	alveolar ridg	1	0	0		1	1	x	x		0	0000/00/00	0	2	
1	14.66	0	62	rmt	0	0	0		1	1	x	x		0	2015/8/28	1	2	
1	51.67	1	75	alveolar ridg	0	0			1	1	x	x		0	0000/00/00	0	2	

1	45.84	0	50	lip	1	0	1	3	3	0	0	0	0000/00/00	0	2	0
1	51.51	1	61	ant tongue	0	0	0	1	1	x	x	0	0000/00/00	0	1	
1	33.15	0	66	buccal muco	1	0	1	3	3	1	0	1	0000/00/00	0	2	1
1	51.51	1	65	ant tongue	0	1	1	1	1	x	x	0	0000/00/00	0	1	
1	51.34	1	56	buccal muco	1	0	1	2	2	0	0	0	2015/8/4	1	2	0
1	20.98	0	67	ant tongue	1	1	1	4	4	0	0	0	0000/00/00	0	2	0
1	51.11	1	61	buccal muco	0	0	1	1	1	0	0	0	0000/00/00	0	2	0
1	51.11	1	51	ant tongue	0	0	0	1	1	0	0	0	0000/00/00	0	2	0
1	51.05	1	60	buccal muco	0	0	0	1	1	x	x	0	0000/00/00	0	2	
1	7.48	0	75	alveolar ridge	1	0	0	4	4	2	0	0	2015/8/11	1	2	1
1	51.51	1	61	alveolar ridge	0	0	0	4	4	x	x	0	0000/00/00	0	1	
1	50.85	1	55	ant tongue	1	1	0	4	4	0	0	0	0000/00/00	0	2	0
1	51.77	1	41	buccal muco	0	0	0	1	1	0	0	0	0000/00/00	0	2	0
1	26.98	0	47	buccal muco	1	0	1	4	2	2	0	0	0000/00/00	0	2	1
1	50.79	1	69	buccal muco	1	0	1	1	1	x	x	0	0000/00/00	0	2	
1	50.79	1	53	alveolar ridge	1	0	1	4	2	2	0	0	0000/00/00	0	2	1
1	51.54	1	50	buccal muco	0	0	0	1	1	0	0	0	0000/00/00	0	1	0
1	50.66	1	60	rmt	1	0	1	2	2	0	0	0	0000/00/00	0	2	0
1	50.66	1	64	ant tongue	1	1		1	1	0	0	0	0000/00/00	0	2	0
1	50.66	1	48	ant tongue	1	0	0	4	4	x	x	0	0000/00/00	0	2	
1	52.00	1	61	lip	1	1		2	2	0	0	0	0000/00/00	0	2	0
1	8.49	0	59	alveolar ridge	0	0	1	2	2	0	0	0	0000/00/00	0	2	0
1	50.62	1	55	alveolar ridge	1	0	1	4	4	2	0	0	0000/00/00	0	2	1
1	50.98	1	53	buccal muco	0	0	0	3	1	1	0	0	0000/00/00	0	2	1
1	50.75	1	79	buccal muco	1	0	0	1	1	x	x	0	0000/00/00	0	1	
1	50.39	1	41	lip	1	0	1	2	2	x	x	0	2017/4/6	1	2	
1	51.51	1	48	ant tongue	1	1	1	3	3	0	0	0	0000/00/00	0	2	0
1	4.26	0	77	alveolar ridge	0	0	0	4	1	2	1	0	0000/00/00	0	2	1
1	50.36	1	57	lip	0	0	0	1	1	x	x	0	0000/00/00	0	1	
1	50.89	1	62	buccal muco	0	0	0	2	2	0	0	0	0000/00/00	0	1	0
1	4.23	0	52	lip	1	0	0	2	2	x	x	0	0000/00/00	0	3	
1	50.13	1	70	buccal muco	0	0	0	1	1	x	x	0	2015/9/16	1	2	
1	50.13	1	57	ant tongue	1	0	0	1	1	0	0	0	0000/00/00	0	2	0
1	50.13	1	70	ant tongue	0	0	0	1	1	0	0	0	0000/00/00	0	2	0
1	50.16	1	41	ant tongue	1	1	1	1	1	0	0	0	0000/00/00	0	2	0
1	23.34	0	54	buccal muco	0	0	1	3	3	1	1	0	2015/12/30	1	3	1
1	50.16	1	56	buccal muco	0	0	1	1	1	x	x	0	0000/00/00	0	1	
1	52.89	1	78	buccal muco	0	0	0	無法	1	x	x	0	2016/4/13	1	1	
1	16.13	0	50	buccal muco	1	0		3	1	1	1	0	0000/00/00	0	3	1
1	49.97	1	60	buccal muco	0	0	0	1	1	0	0	0	0000/00/00	0	2	0
1	50.20	1	49	buccal muco	1	0	0	1	1	0	0	0	2015/8/18	1	2	0
1	50.13	1	83	alveolar ridge	0	0	0	4	4	1	0	0	0000/00/00	0	2	1
1	18.95	0	62	buccal muco	1	1	1	1	1	x	x	0	0000/00/00	0	2	
1	50.52	1	73	rmt	0	0	0	4	4	0	0	0	0000/00/00	0	1	0
1	49.84	1	65	buccal muco	1	1	1	1	1	x	x	0	0000/00/00	0	2	
1	49.84	1	66	buccal muco	1	0	0	1	1	0	0	0	0000/00/00	0	2	0
1	36.85	0	46	ant tongue	1	1	1	2	2	0	0	0	0000/00/00	0	2	0
1	50.52	1	58	alveolar ridge	0	0	0	4	4	0	0	0	0000/00/00	0	2	0
1	52.23	1	72	alveolar ridge	0	0	0	2	2	0	0	0	0000/00/00	0	1	0
1	49.67	1	60	hard palate	1	1	1	1	1	x	x	0	0000/00/00	0	2	
1	49.38	1	54	buccal muco	1	0	1	1	1	x	x	0	0000/00/00	0	2	
1	19.31	0	62	buccal muco	0	0	0	4	4	0	0	0	0000/00/00	0	2	0
1	49.48	1	64	ant tongue	0	1	1	4	4	2	1	0	0000/00/00	0	2	1
1	49.41	1	54	buccal muco	1	0	0	1	1	x	x	0	0000/00/00	0	2	
1	21.77	0	36	alveolar ridge	1	0	1	4	4	2	1	0	2015/11/17	1	2	1
1	49.21	1	54	buccal muco	1	1	1	1	1	x	x	0	0000/00/00	0	1	
1	49.44	1	55	buccal muco	1	0	0	1	1	0	0	0	0000/00/00	0	2	0
1	49.11	1	77	lip	1	1	0	2	2	0	0	0	0000/00/00	0	2	0
1	49.11	1	43	ant tongue	1	1	1	3	1	1	1	0	0000/00/00	0	2	1
1	49.15	1	46	buccal muco	1	1	1	1	1	0	0	0	0000/00/00	0	2	0
1	49.38	1	50	rmt	0	0	0	無法	1	x	x	0	0000/00/00	0	2	
1	49.21	1	47	alveolar ridge	1	1	1	2	2	0	0	0	2015/10/30	1	2	0
1	48.92	1	70	buccal muco	1	1	1	2	2	0	0	0	0000/00/00	0	2	0
1	49.34	1	42	ant tongue	1	0	1	2	2	0	0	0	0000/00/00	0	2	0
1	49.51	1	71	ant tongue	1	1	0	1	1	0	0	0	0000/00/00	0	2	0
1	21.31	0	81	rmt	0	0	0	4	4	0	0	0	2015/10/23	1	2	0
1	19.21	0	59	buccal muco	1	1	1	4	1	2	1	0	2015/10/14	1	2	1
1	15.28	0	63	mouth floor	1	1	1	3	2	1	0	0	0000/00/00	0	2	1
1	48.89	1	54	lip	1	0	0	1	1	x	x	0	0000/00/00	0	1	
1	9.11	0	64	alveolar ridge	1	1	1	4	4	2	1	0	2016/1/27	1	2	1
1	48.69	1	72	buccal muco	1	1	1	1	1	0	0	0	0000/00/00	0	1	0
1	48.49	1	48	mouth floor	1	1	1	1	1	0	0	0	0000/00/00	0	2	0

1	45.28	0	76	alveolar ridge	1	1	1	4	4	0	0	0	0000/00/00	0	2	0
1	48.59	1	57	buccal muco	1	1		1	1	0	0	0	0000/00/00	0	2	0
1	48.82	1	47	buccal muco	1	1		1	1	x	x	0	0000/00/00	0	1	
1	48.66	1	41	ant tongue	1	1	1	3	1	1	1	0	2017/12/19	1	3	1
1	42.03	0	62	alveolar ridge	0	0	1	3	1	1	1	0	0000/00/00	0	3	1
1	48.33	1	55	ant tongue	1	1	0	4	4	0	0	0	0000/00/00	0	3	0
1	22.26	0	79	buccal muco	1	1		2	2	0	0	0	2016/9/9	1	2	0
1	48.33	1	56	mouth floor	1	1	1	4	4	2	1	0	0000/00/00	0	3	1
1	21.38	0	68	buccal muco	1	1	1	2	2	0	0	0	0000/00/00	0	2	0
1	49.84	1	58	buccal muco	1	0		3	2	1	0	0	0000/00/00	0	2	1
1	19.90	0	64	ant tongue	1	1	0	1	1	0	0	0	2016/1/4	1	2	0
1	5.05	0	49	alveolar ridge	1	1		4	4	2	1	0	0000/00/00	0	2	1
1	15.21	0	44	alveolar ridge	1	1	1	4	4	x	x	0	0000/00/00	0	2	
1	47.97	1	53	alveolar ridge	1	1	0	1	1	x	x	0	0000/00/00	0	1	
1	47.84	1	45	buccal muco	1	1	0	1	1	0	0	0	0000/00/00	0	2	0
1	14.46	0	57	ant tongue	1	0	0	4	4	x	x	0	0000/00/00	0	2	
1	22.20	0	47	alveolar ridge	1	1	1	4	4	0	0	0	0000/00/00	0	3	0
1	47.84	1	49	lip	1	1	1	1	1	x	x	0	0000/00/00	0	1	
1	48.30	1	56	alveolar ridge	1	1	1	1	1	0	0	0	0000/00/00	0	2	0
1	11.15	0	42	ant tongue	1	1	1	4	4	0	0	0	0000/00/00	0	2	0
1	28.72	0	60	buccal muco	1	1	1	3	2	1	0	0	0000/00/00	0	2	1
1	47.64	1	63	rmt	1	1	1	1	1	0	0	0	0000/00/00	0	2	0
1	47.67	1	46	ant tongue	1	1	1	無法	1	x	x	0	0000/00/00	0	2	
1	47.54	1	50	alveolar ridge	1	1	1	4	4	0	0	0	0000/00/00	0	2	0
1	47.61	1	60	buccal muco	1	1		1	1	x	x	0	0000/00/00	0	1	
1	48.00	1	50	ant tongue	1	1	1	2	2	0	0	0	0000/00/00	0	3	0
1	47.41	1	44	buccal muco	1	1	1	2	2	0	0	0	0000/00/00	0	2	0
1	12.92	0	61	ant tongue	0	0	1	4	2	2	1	0	2016/5/5	1	2	1
1	47.97	1	67	lip	1	1	0	1	1	0	0	0	0000/00/00	0	1	0
1	47.31	1	84	alveolar ridge	1	1	1	1	1	x	x	0	0000/00/00	0	1	
1	47.87	1	56	ant tongue	1	1	0	1	1	x	x	0	0000/00/00	0	2	
1	47.31	1	54	buccal muco	1	1	1	4	4	0	0	0	0000/00/00	0	1	0
1	48.59	1	56	buccal muco	1	1	1	4	4	1	1	0	0000/00/00	0	2	1
1	42.49	0	56	alveolar ridge	1	1	0	4	4	0	0	0	0000/00/00	0	2	0
1	45.64	0	77	ant tongue	1	1	0	4	4	x	x	0	0000/00/00	0	2	
1	47.05	1	58	alveolar ridge	1	0	0	4	4	0	0	0	0000/00/00	0	2	0
1	9.11	0	74	buccal muco	1	1	1	2	2	x	x	0	0000/00/00	0	2	
1	47.97	1	67	alveolar ridge	1	1	1	1	1	x	x	0	0000/00/00	0	1	
1	20.23	0	47	ant tongue	1	1	1	1	1	0	0	0	0000/00/00	0	2	0
1	46.85	1	40	buccal muco	1	1	1	4	4	2	0	0	0000/00/00	0	2	1
1	47.87	1	53	ant tongue	1	0	0	1	1	x	x	0	2015/8/24	1	1	
1	50.49	1	63	buccal muco	0	0		1	1	x	x	0	2016/9/29	1	1	
1	22.30	0	49	ant tongue	1	1	1	2	2	x	x	0	0000/00/00	0	3	
1	46.69	1	60	buccal muco	1	1	1	1	1	0	0	0	0000/00/00	0	1	0
1	46.52	1	62	buccal muco	1	1	0	1	1	x	x	0	0000/00/00	0	1	
1	47.18	1	73	ant tongue	1	0	1	1	1	x	x	0	0000/00/00	0	2	
1	46.69	1	53	ant tongue	1	1		4	4	2	1	0	0000/00/00	0	2	1
1	46.46	1	59	buccal muco	0	1		1	1	x	x	0	0000/00/00	0	2	
1	46.46	1	56	buccal muco	1	1	1	1	1	x	x	0	0000/00/00	0	1	
1	46.72	1	75	buccal muco	1	1	1	1	1	0	0	0	0000/00/00	0	2	0
1	46.49	1	70	mouth floor	1	1		1	1	x	x	0	2016/5/2	1	2	
1	46.49	1	73	ant tongue	1	1	0	1	1	0	0	0	0000/00/00	0	2	0
1	46.26	1	61	ant tongue	1	1	1	2	2	0	0	0	0000/00/00	0	3	0
1	46.46	1	65	lip	1	1	1	1	1	0	0	0	0000/00/00	0	2	0
1	20.56	0	84	ant tongue	1	1	0	無法	1	x	x	0	0000/00/00	0	2	
1	47.08	1	77	ant tongue	0	1	0	2	2	x	x	0	0000/00/00	0	2	
1	46.16	1	62	alveolar ridge	1	1	1	2	2	0	0	0	2018/2/13	1	2	0
1	6.26	0	60	alveolar ridge	1	1		4	4	x	x	1	0000/00/00	0	2	
1	46.03	1	54	buccal muco	1	1	1	4	4	2	1	0	0000/00/00	0	2	1
1	46.46	1	55	lip	1	1	1	1	1	0	0	0	0000/00/00	0	1	0
1	6.03	0	50	ant tongue	1	1	1	1	1	0	0	0	2015/10/19	1	2	0
1	46.46	1	55	buccal muco	1	1	1	1	1	0	0	0	0000/00/00	0	1	0
1	46.16	1	48	buccal muco	1	1	1	3	3	0	0	0	0000/00/00	0	2	0
1	47.90	1	63	alveolar ridge	1	1	1	無法	3	x	x	0	0000/00/00	0	3	
1	37.74	0	66	alveolar ridge	1	1	0	4	4	x	x	0	0000/00/00	0	2	
1	45.80	1	54	alveolar ridge	1	1	1	2	2	0	0	0	0000/00/00	0	2	0
1	10.43	0	68	alveolar ridge	1	1	1	4	4	2	0	0	0000/00/00	0	2	1
1	46.00	1	64	lip	0	1	1	1	1	x	x	0	0000/00/00	0	2	
1	45.74	1	46	buccal muco	1	1	1	1	1	0	0	0	0000/00/00	0	2	0
1	46.00	1	64	buccal muco	0	1	1	1	1	x	x	0	0000/00/00	0	2	
1	18.98	0	41	ant tongue	1	0		4	4	x	x	0	2016/3/9	1	3	
1	28.46	0	66	alveolar ridge	1	1		2	2	x	x	0	0000/00/00	0	2	

1	32.75	0	49	ant tongue	1	1		1	1	0	0	0	2016/3/4	1	2	0
1	45.31	1	57	ant tongue	0	0	1	1	1	x	x	0	0000/00/00	0	1	
1	45.38	1	40	rmt	1	1	1	1	1	x	x	0	2017/11/25	1	2	
1	45.48	1	46	ant tongue	1	1	0	4	4	0	0	0	0000/00/00	0	2	0
1	45.57	1	58	buccal muco	1	0	1	2	2	0	0	0	0000/00/00	0	2	0
1	45.67	1	40	ant tongue	1	1	0	4	3	2	0	0	0000/00/00	0	2	1
1	29.34	0	63	rmt	1	1	0	1	1	0	0	0	2016/3/21	1	2	0
1	45.25	1	57	buccal muco	1	1	1	4	4	1	1	0	0000/00/00	0	2	1
1	45.28	1	61	alveolar ridg	1	1	0	1	1	x	x	0	0000/00/00	0	2	
1	58.62	1	58	hard palate	1	1	1	1	1	0	0	0	0000/00/00	0	1	0
1	58.62	1	58	lip	1	1	1	1	1	0	0	0	0000/00/00	0	1	0
1	12.20	0	55	lip	1	1	1	1	1	x	x	0	0000/00/00	0	2	
1	45.05	1	57	rmt	1	1	1	1	1	x	x	0	2016/1/13	1	3	
1	46.26	1	37	buccal muco	1	1	1	4	4	0	0	0	0000/00/00	0	2	0
1	44.98	1	44	ant tongue	1	1	1	4	4	2	1	0	0000/00/00	0	2	1
1	22.26	0	51	ant tongue	1	1	1	1	1	0	0	0	2016/2/15	1	2	0
1	45.25	1	41	alveolar ridg	1	1	1	4	4	x	x	0	0000/00/00	0	2	
1	45.31	1	51	alveolar ridg	1	1	1	無法	1	x	x	0	0000/00/00	0	2	
1	5.05	0	76	lip	1	1		4	3	2	1	0	2015/10/24	1	3	1
1	44.92	1	42	ant tongue	1	0	0	4	4	0	0	0	0000/00/00	0	2	0
1	6.79	0	76	alveolar ridg	1	0	0	1	1	x	x	0	2015/12/4	1	2	
1	62.07	1	44	buccal muco	1	0	0	1	1	0	0	0	0000/00/00	0	2	0
1	59.93	1	43	rmt	1	1	1	4	4	0	0	0	0000/00/00	0	2	0
1	44.66	1	57	buccal muco	1	1	1	4	2	2	0	0	0000/00/00	0	2	1
1	44.79	1	45	buccal muco	1	1	1	2	2	0	0	0	0000/00/00	0	2	0
1	44.62	1	63	lip	1	1	1	1	1	x	x	0	0000/00/00	0	1	
1	44.52	1	74	lip	1	1	1	2	2	0	0	0	0000/00/00	0	2	0
1	44.46	1	56	ant tongue	1	1	1	4	4	0	0	0	0000/00/00	0	1	0
1	44.66	1	64	ant tongue	1	0	0	2	2	0	0	0	0000/00/00	0	2	0
1	57.05	1	59	lip	1	1		1	1	0	0	0	0000/00/00	0	2	0
1	44.33	1	43	buccal muco	1	1	1	4	2	2	1	0	0000/00/00	0	2	1
1	44.33	1	47	ant tongue	1	1	1	4	4	0	0	0	0000/00/00	0	2	0
1	44.62	1	61	buccal muco	1	1	0	4	4	0	0	0	0000/00/00	0	2	0
1	14.30	0	53	ant tongue	1	1	1	4	4	0	0	0	2016/5/16	1	2	0
1	44.33	1	50	alveolar ridg	1	1	0	1	1	x	x	0	0000/00/00	0	2	
1	58.43	1	50	ant tongue	0	0	0	1	1	0	0	0	0000/00/00	0	1	0
1	41.41	0	79	buccal muco	1	1	1	2	2	0	0	0	0000/00/00	0	2	0
1	6.95	0	48	ant tongue	1	1	1	4	4	2	1	0	2016/3/18	1	2	1
1	43.87	1	57	buccal muco	1	1	0	1	1	0	0	0	0000/00/00	0	2	0
1	2.92	0	46	ant tongue	1	1	1	4	4	2	1	1	0000/00/00	0	2	1
1	8.69	0	58	alveolar ridg	1	1	1	4	4	x	x	0	2016/3/3	1	2	
1	43.77	1	56	lip	1	1	1	1	1	x	x	0	0000/00/00	0	1	
1	44.20	1	73	buccal muco	1	1	1	2	2	x	x	0	0000/00/00	0	1	
1	7.54	0	61	buccal muco	0	0	0	4	4	x	x	0	0000/00/00	0	2	
1	43.70	1	53	buccal muco	1	1	1	2	2	0	0	0	0000/00/00	0	2	0
1	43.64	1	48	ant tongue	1	1	1	1	1	x	x	0	0000/00/00	0	2	
1	43.87	1	56	alveolar ridg	1	1	1	2	2	x	x	0	0000/00/00	0	2	
1	19.90	0	57	rmt	1	1	1	4	4	0	0	0	2016/5/10	1	2	0
1	43.31	1	54	buccal muco	1	1	0	4	2	2	0	0	0000/00/00	0	2	1
1	43.31	1	69	lip	0	0	0	2	2	0	0	0	0000/00/00	0	2	0
1	43.54	1	42	buccal muco	1	1	1	2	2	0	0	0	0000/00/00	0	2	0
1	43.70	1	62	ant tongue	1	1	0	1	1	0	0	0	0000/00/00	0	1	0
1	43.54	1	62	hard palate	1	0	1	無法	1	x	x	0	0000/00/00	0	2	
1	43.54	1	40	ant tongue	1	1	0	4	4	1	0	1	0000/00/00	0	2	1
1	43.48	1	64	lip	1	1		2	2	0	0	0	0000/00/00	0	2	0
1	43.25	1	65	ant tongue	1	1	0	1	1	0	0	0	0000/00/00	0	2	0
1	43.77	1	70	ant tongue	1	1	0	2	2	0	0	0	0000/00/00	0	2	0
1	43.18	1	39	ant tongue	1	1	1	1	1	0	0	0	0000/00/00	0	2	0
1	43.08	1	60	ant tongue	1	1	1	3	1	1	0	0	0000/00/00	0	2	1
1	37.25	0	52	alveolar ridg	1	1	1	2	2	0	0	0	0000/00/00	0	2	0
1	43.97	1	54	ant tongue	1	0	1	1	1	x	x	0	0000/00/00	0	1	
1	43.48	1	59	ant tongue	1	1	1	3	3	0	0	0	0000/00/00	0	2	0
1	42.98	1	56	rmt	1	1	1	2	2	0	0	0	0000/00/00	0	2	0
1	42.95	1	56	lip	1	1	1	1	1	x	x	0	0000/00/00	0	2	
1	43.74	1	43	ant tongue	1	1	1	4	4	0	0	0	0000/00/00	0	3	0
1	43.21	1	59	buccal muco	1	1	1	2	2	0	0	0	0000/00/00	0	2	0
1	42.98	1	31	buccal muco	1	1	0	4	4	1	1	0	0000/00/00	0	1	1
1	43.51	1	70	lip	1	1	0	1	1	x	x	0	2016/6/29	1	1	
1	43.28	1	58	ant tongue	0	1	0	1	1	x	x	0	0000/00/00	0	2	
1	42.59	1	60	ant tongue	1	1	1	1	1	0	0	0	0000/00/00	0	2	0
1	19.90	0	51	rmt	1	1	1	1	1	x	x	0	0000/00/00	0	2	
1	36.10	0	70	buccal muco	1	1	1	1	1	0	0	0	0000/00/00	0	2	0

1	42.56	1	54	ant tongue	1	1	1	1	1	0	0	0	0000/00/00	0	2	0
1	7.97	0	52	ant tongue	1	1	0	4	4	1	0	1	0000/00/00	0	2	1
1	5.41	0	80	lip	1	1	1	3	3	0	0	0	0000/00/00	0	2	0
1	42.39	1	62	alveolar ridge	0	1	0	4	4	0	0	0	0000/00/00	0	2	0
1	43.08	1	54	ant tongue	1	1	1	1	1	x	x	0	0000/00/00	0	1	
1	44.16	1	43	ant tongue	1	1	0	1	1	0	0	0	0000/00/00	0	1	0
1	42.39	1	51	ant tongue	1	1	1	3	3	0	0	0	0000/00/00	0	2	0
1	42.75	1	60	buccal muco	1	1	1	2	2	0	0	0	0000/00/00	0	2	0
1	42.75	1	59	buccal muco	1	1	0	1	1	0	0	0	0000/00/00	0	2	0
1	41.77	1	85	lip	1	0	0	1	1	x	x	0	0000/00/00	0	1	
1	42.72	1	36	alveolar ridge	1	1	1	4	4	0	0	0	0000/00/00	0	1	0
1	54.00	1	56	buccal muco	1	1	1	2	2	0	0	1	0000/00/00	0	1	0
1	54.00	1	56	lip	1	1	1	1	1	0	0	0	0000/00/00	0	1	0
1	42.95	1	60	ant tongue	1	1	1	1	1	x	x	0	0000/00/00	0	2	
1	42.07	1	55	lip	1	1	0	1	1	0	0	0	0000/00/00	0	2	0
1	42.10	1	61	alveolar ridge	1	1	1	4	4	0	0	0	0000/00/00	0	2	0
1	42.33	1	54	mouth floor	1	1	0	4	2	2	1	0	0000/00/00	0	2	1
1	42.10	1	72	ant tongue	1	1	1	1	1	x	x	0	0000/00/00	0	2	
1	42.10	1	57	rmt	1	1	1	4	4	0	0	0	0000/00/00	0	1	0
1	42.13	1	67	buccal muco	1	0	0	1	1	x	x	1	2016/4/2	1	2	
1	41.93	1	41	lip	1	1	1	1	1	0	0	0	0000/00/00	0	2	0
1	42.56	1	56	alveolar ridge	1	1	1	1	1	x	x	0	0000/00/00	0	2	
1	14.16	0	57	ant tongue	1	1	1	4	4	2	0	0	2016/7/27	1	2	1
1	41.87	1	55	lip	1	1	0	1	1	0	0	0	0000/00/00	0	1	0
1	5.77	0	60	buccal muco	1	1	1	3	3	0	0	0	0000/00/00	0	1	0
1	41.70	1	62	alveolar ridge	1	1	1	1	1	0	0	0	0000/00/00	0	2	0
1	41.80	1	63	ant tongue	1	1	1	4	4	0	0	0	0000/00/00	0	2	0
1	41.67	1	53	alveolar ridge	1	1	1	4	4	0	0	0	0000/00/00	0	2	0
1	18.66	0	54	buccal muco	1	0	1	3	2	1	1	0	2016/7/25	1	2	1
1	9.70	0	56	alveolar ridge	1	1	1	4	4	2	1	0	0000/00/00	0	2	1
1	21.80	0	67	lip	1	1	1	1	1	0	0	0	0000/00/00	0	2	0
1	41.64	1	56	ant tongue	1	1	1	1	1	0	0	0	0000/00/00	0	2	0
1	9.93	0	60	ant tongue	1	1	1	4	4	2	1	0	2016/5/13	1	2	1
1	41.48	1	49	ant tongue	0	0	0	1	1	x	x	0	0000/00/00	0	1	
1	41.41	1	54	buccal muco	1	1	1	1	1	x	x	0	0000/00/00	0	2	
1	24.33	0	43	buccal muco	1	1	0	4	4	2	1	0	2017/9/6	1	2	1
1	41.44	1	63	buccal muco	1	1	1	1	1	x	x	0	0000/00/00	0	2	
1	41.44	1	64	buccal muco	1	1		1	1	0	0	0	0000/00/00	0	1	0
1	41.38	1	44	mouth floor	1	1	1	1	1	0	0	0	0000/00/00	0	2	0
1	14.56	0	62	alveolar ridge	1	1	0	1	1	x	x	0	2014/12/9	1	1	
1	13.41	0	62	ant tongue	1	1	0	1	1	x	x	0	0000/00/00	0	2	
1	41.08	1	64	buccal muco	1	1	1	2	2	0	0	0	0000/00/00	0	2	0
1	10.62	0	43	alveolar ridge	1	1		4	4	0	0	1	0000/00/00	0	2	0
1	2.59	0	56	ant tongue	1	1	1	4	4	2	1	0	0000/00/00	0	2	1
1	41.44	1	64	buccal muco	0	0	0	3	1	1	0	0	0000/00/00	0	1	1
1	41.02	1	48	alveolar ridge	1	1	1	4	4	0	0	0	0000/00/00	0	2	0
1	39.48	0	45	rmt	1	1	1	2	2	0	0	0	0000/00/00	0	2	0
1	29.02	0	81	ant tongue	1	1	0	2	2	x	x	0	0000/00/00	0	2	
1	40.43	1	57	buccal muco	1	1		3	3	0	0	0	0000/00/00	0	2	0
1	15.70	0	62	rmt	1	1		1	1	x	x	0	0000/00/00	0	2	
1	13.05	0	48	buccal muco	1	1	1	1	1	x	x	0	0000/00/00	0	2	
1	41.18	1	54	lip	1	1		1	1	0	0	0	0000/00/00	0	1	0
1	41.21	1	63	lip	0	0		1	1	0	0	0	0000/00/00	0	2	0
1	40.95	1	63	alveolar ridge	1	1		4	4	2	0	0	0000/00/00	0	2	1
1	32.72	0	57	rmt	1	1	0	2	2	x	x	0	0000/00/00	0	2	
1	41.08	1	66	buccal muco	1	1	1	2	2	0	0	0	0000/00/00	0	2	0
1	40.43	1	63	rmt	0	0		1	1	0	0	0	0000/00/00	0	2	0
1	8.39	0	45	mouth floor	1	1	1	4	3	2	1	0	0000/00/00	0	2	1
1	40.39	1	71	buccal muco	1	1		1	1	0	0	0	2016/4/20	1	3	0
1	41.34	1	63	buccal muco	0	0	0	2	2	x	x	0	0000/00/00	0	1	
1	40.46	1	66	alveolar ridge	1	1	1	2	2	0	0	0	0000/00/00	0	2	0
1	40.26	1	55	buccal muco	1	0	0	2	2	0	0	0	0000/00/00	0	2	0
1	13.41	0	56	rmt	1	1	1	4	4	0	0	0	0000/00/00	0	1	0
1	40.23	1	42	buccal muco	1	1	1	4	4	1	0	0	0000/00/00	0	2	1
1	41.21	1	52	buccal muco	1	0	0	1	1	x	x	0	2016/8/17	1	2	
1	40.30	1	65	buccal muco	1	1	1	1	1	0	0	0	0000/00/00	0	3	0
1	40.56	1	44	buccal muco	1	1	0	2	2	0	0	0	0000/00/00	0	2	0
1	39.90	1	38	ant tongue	1	1		4	4	2	1	0	0000/00/00	0	2	1
1	39.87	1	68	lip	1	1		4	1	2	1	0	0000/00/00	0	2	1
1	39.87	1	87	ant tongue	0	0	0	2	2	x	x	0	0000/00/00	0	2	
1	12.46	0	85	rmt	1	1		4	4	2	1	0	0000/00/00	0	2	1
1	39.74	1	57	alveolar ridge	1	1	0	1	1	0	0	0	0000/00/00	0	2	0

1	39.48	1	54	hard palate	1	1		2	2	0	0	0	0000/00/00	0	2	0
1	39.84	1	68	buccal muco	1	1	0	2	2	x	x	0	0000/00/00	0	3	
1	39.38	1	71	rmt	1	1		1	1	x	x	0	0000/00/00	0	1	
1	62.95	1	65	hard palate	1	1		4	4	0	0	0	2016/1/29	1	1	0
1	39.54	1	55	alveolar ridg	1	1	1	4	4	1	0	0	0000/00/00	0	2	1
1	39.38	1	41	ant tongue	1	0	1	2	2	0	0	0	0000/00/00	0	2	0
1	4.62	0	66	buccal muco	1	1	0	4	4	2	0	0	2016/6/7	1	2	1
1	39.84	1	76	alveolar ridg	1	1	1	4	4	x	x	0	0000/00/00	0	2	
1	39.08	1	40	buccal muco	1	1		2	2	0	0	0	0000/00/00	0	2	0
1	39.18	1	60	ant tongue	1	1	1	1	1	0	0	0	0000/00/00	0	2	0
1	6.52	0	57	ant tongue	1	1	1	4	4	2	1	0	2016/8/10	1	2	1
1	39.80	1	60	ant tongue	1	1	1	1	1	0	0	0	0000/00/00	0	1	0
1	39.28	1	79	buccal muco	1	1	1	2	2	0	0	0	0000/00/00	0	2	0
1	39.34	1	55	buccal muco	1	1	1	1	1	x	x	0	0000/00/00	0	2	
1	14.95	0	41	ant tongue	1	1	1	4	4	0	0	0	0000/00/00	0	2	0
1	40.33	1	63	alveolar ridg	1	1		3	3	0	0	0	0000/00/00	0	2	0
1	38.92	1	62	ant tongue	1	1	1	1	1	0	0	0	0000/00/00	0	2	0
1	30.07	0	58	buccal muco	1	1		1	1	x	x	0	0000/00/00	0	2	
1	39.38	1	62	alveolar ridg	1	1	1	4	4	0	0	0	0000/00/00	0	1	0
1	38.92	1	34	ant tongue	1	1	1	3	2	1	0	0	0000/00/00	0	2	1
1	38.85	1	68	buccal muco	0	0	0	1	1	0	0	0	0000/00/00	0	2	0
1	1.31	0	53	buccal muco	1	1	1	2	2	0	0	0	0000/00/00	0	2	0
1	10.03	0	45	buccal muco	1	1	1	3	3	0	0	0	0000/00/00	0	2	0
1	38.66	1	54	ant tongue	1	1	1	1	1	x	x	0	0000/00/00	0	2	
1	38.49	1	73	buccal muco	1	1	1	2	2	0	0	0	0000/00/00	0	2	0
1	39.38	1	46	buccal muco	1	1	1	1	1	x	x	0	2016/7/25	1	1	
1	38.59	1	65	buccal muco	1	0		4	4	0	0	0	0000/00/00	0	2	0
1	38.59	1	52	ant tongue	1	1	1	4	4	0	0	0	0000/00/00	0	1	0
1	15.87	0	78	hard palate	1	1	1	2	2	0	0	0	2016/11/15	1	2	0
1	39.15	1	49	lip	1	0	1	1	1	x	x	0	0000/00/00	0	1	
1	38.79	1	79	buccal muco	1	1	0	4	2	2	0	0	0000/00/00	0	2	1
1	17.31	0	73	alveolar ridg	1	1	0	1	1	x	x	1	0000/00/00	0	2	
1	38.33	1	53	ant tongue	1	1	1	2	2	0	0	0	0000/00/00	0	2	0
1	38.36	1	56	buccal muco	1	1		1	1	x	x	0	0000/00/00	0	2	
1	38.66	1	56	buccal muco	1	0	0	1	1	0	0	0	2016/6/10	1	2	0
1	24.85	0	53	buccal muco	1	1	1	1	1	x	x	0	0000/00/00	0	2	
1	38.16	1	52	ant tongue	1	0	1	3	2	1	0	0	0000/00/00	0	2	1
1	38.23	1	34	ant tongue	1	1	1	4	4	2	0	0	0000/00/00	0	2	1
1	38.23	1	34	alveolar ridg	1	1	0	1	1	0	0	0	0000/00/00	0	2	0
1	6.69	0	66	rmt	1	1	1	4	4	x	x	0	2016/9/3	1	2	
1	38.26	1	52	ant tongue	1	0	0	2	2	0	0	0	0000/00/00	0	3	0
1	29.08	0	67	alveolar ridg	1	1	1	4	4	0	0	0	0000/00/00	0	1	0
1	38.10	1	81	ant tongue	0	0		1	1	x	x	0	0000/00/00	0	2	
1	37.90	1	66	buccal muco	1	1	1	1	1	0	0	0	0000/00/00	0	2	0
1	22.82	0	58	ant tongue	1	1	1	4	4	1	1	1	2016/6/7	1	3	1
1	38.16	1	57	lip	1	1		1	1	x	x	0	0000/00/00	0	1	
1	38.20	1	44	buccal muco	1	1		1	1	0	0	0	0000/00/00	0	2	0
1	38.23	1	50	alveolar ridg	1	1	0	1	1	x	x	0	2018/5/23	1	2	
1	2.20	0	53	buccal muco	1	1	1	1	1	0	0	0	0000/00/00	0	2	0
1	37.67	1	59	buccal muco	1	1	0	2	2	0	0	0	0000/00/00	0	2	0
1	37.64	1	63	ant tongue	0	0	1	2	2	0	0	0	0000/00/00	0	1	0
1	37.54	1	81	lip	1	1	1	1	1	0	0	0	0000/00/00	0	1	0
1	14.23	0	59	ant tongue	1	1	0	1	1	x	x	0	2016/9/20	1	2	
1	7.41	0	59	mouth floor	1	1		4	1	2	1	0	0000/00/00	0	2	1
1	20.59	0	60	alveolar ridg	1	1		4	4	x	x	0	2016/12/28	1	2	
1	37.90	1	61	ant tongue	1	1	1	4	2	2	1	0	0000/00/00	0	2	1
1	37.54	1	71	alveolar ridg	0	0	1	1	1	x	x	0	0000/00/00	0	2	
1	11.93	0	83	buccal muco	1	1	1	無法	1	x	x	0	0000/00/00	0	2	
1	37.44	1	40	ant tongue	1	0	0	1	1	0	0	0	0000/00/00	0	2	0
1	9.61	0	64	buccal muco	1	0	1	1	1	x	x	0	2016/8/31	1	2	
1	37.44	1	57	ant tongue	1	1	1	2	2	0	0	0	0000/00/00	0	2	0
1	37.28	1	66	lip	1	1	0	1	1	x	x	0	0000/00/00	0	2	
1	33.70	0	44	rmt	1	0		4	1	2	1	0	2017/5/4	1	3	1
1	38.00	1	55	alveolar ridg	1	1	1	2	2	x	x	0	0000/00/00	0	3	
1	37.70	1	57	buccal muco	1	1	1	1	1	x	x	1	0000/00/00	0	2	
1	37.11	1	50	buccal muco	1	1	0	2	2	0	0	0	0000/00/00	0	1	0
1	37.44	1	37	ant tongue	0	0	0	1	1	0	0	0	0000/00/00	0	2	0
1	37.44	1	79	alveolar ridg	0	0	0	4	4	1	0	0	0000/00/00	0	2	1
1	37.08	1	59	buccal muco	1	0	0	1	1	x	x	0	0000/00/00	0	1	
1	31.38	0	85	ant tongue	0	0	0	1	1	x	x	0	2016/8/31	1	3	
1	18.30	0	41	rmt	1	1	1	4	4	0	0	0	2016/11/11	1	2	0
1	37.05	1	51	buccal muco	1	1	1	3	3	0	0	0	0000/00/00	0	2	0

1	37.90	1	64	buccal muco	1	1	1	1	1		0	0	0	0000/00/00	0	1	0
1	36.95	1	63	alveolar ridg	1	1	1	4	4	x	x		0	0000/00/00	0	2	
1	36.98	1	63	buccal muco	1	1	0	1	1		0	0	0	0000/00/00	0	2	0
1	37.51	1	42	lip	1	1	1	2	2		0	0	0	0000/00/00	0	1	0
1	36.03	0	63	lip	1	1		2	2	x	x		0	0000/00/00	0	1	
1	37.44	1	45	buccal muco	1	1	1	1	1		0	0	0	0000/00/00	0	2	0
1	14.13	0	70	buccal muco	1	1	0	4	1		2	0	0	0000/00/00	0	2	1
1	37.08	1	66	alveolar ridg	1	1	1	3	3		0	0	0	0000/00/00	0	1	0
1	35.51	0	59	buccal muco	1	1	1	1	1	x	x		0	0000/00/00	0	3	
1	35.51	0	59	lip	1	1	1	1	1	x	x		0	0000/00/00	0	2	
1	17.21	0	72	alveolar ridg	1	1	1	4	4	x	x		0	2015/4/24	1	2	
1	36.72	1	49	buccal muco	1	1	1	4	4		0	0	0	0000/00/00	0	2	0
1	16.82	0	59	buccal muco	1	1	1	2	2		0	0	0	2017/5/15	1	2	0
1	36.52	1	48	alveolar ridg	1	1	1	4	4		1	0	0	0000/00/00	0	2	1
1	3.38	0	56	ant tongue	1	1	1	4	4		1	0	1	0000/00/00	0	2	1
1	36.59	1	47	buccal muco	1	1		4	4		0	0	0	0000/00/00	0	2	0
1	36.43	1	44	lip	1	1	1	1	1	x	x		0	2016/7/5	1	3	
1	32.43	0	56	ant tongue	1	0	0	1	1		0	0	0	2017/5/31	1	2	0
1	36.56	1	53	buccal muco	1	0	1	2	2		0	0	0	0000/00/00	0	2	0
1	36.49	1	72	ant tongue	1	1		1	1		0	0	0	0000/00/00	0	2	0
1	36.75	1	53	lip	1	1	0	1	1		0	0	0	0000/00/00	0	1	0
1	3.18	0	67	alveolar ridg	1	1	0	4	4		2	1	0	0000/00/00	0	2	1
1	36.43	1	65	ant tongue	1	1	1	4	2		2	0	0	0000/00/00	0	3	1
1	36.16	1	57	buccal muco	1	1	1	1	1	x	x		0	0000/00/00	0	1	
1	36.10	1	77	alveolar ridg	1	1	1	4	4		0	0	0	0000/00/00	0	2	0
1	25.93	0	74	alveolar ridg	1	1	1	2	2		0	0	0	2018/3/9	1	1	0
1	16.46	0	52	ant tongue	1	1	1	4	4	x	x		1	2016/6/7	1	3	
1	37.08	1	49	buccal muco	1	1	1	3	3		0	0	0	0000/00/00	0	3	0
1	36.07	1	65	ant tongue	1	1	0	4	4		0	0	0	0000/00/00	0	1	0
1	0.36	0	68	buccal muco	1	1	1	1	1		0	0	0	0000/00/00	0	1	0
1	36.03	1	43	buccal muco	1	1	1	1	1		0	0	0	0000/00/00	0	2	0
1	36.07	1	56	mouth floor	1	0	1	4	4		2	1	0	0000/00/00	0	2	1
1	12.72	0	58	hard palate	1	1	1	4	2		2	0	0	0000/00/00	0	2	1
1	17.51	0	75	buccal muco	1	1	0	1	1	x	x		0	0000/00/00	0	2	
1	36.66	1	37	buccal muco	1	1		1	1		0	0	0	0000/00/00	0	2	0
1	35.84	1	57	buccal muco	1	1	1	3	1		1	0	0	0000/00/00	0	2	1
1	35.74	1	52	buccal muco	1	1	0	2	2	x	x		0	0000/00/00	0	2	
1	32.46	0	45	rmt	1	1	1	4	4		2	1	0	0000/00/00	0	2	1
1	45.77	1	72	buccal muco	1	1	1	1	1	x	x		0	0000/00/00	0	2	
1	25.70	0	42	buccal muco	1	0	0	1	1		0	0	0	0000/00/00	0	2	0
1	14.62	0	54	lip	1	0	1	1	1	x	x		0	0000/00/00	0	2	
1	35.57	1	43	ant tongue	1	1	1	2	2		0	0	0	0000/00/00	0	2	0
1	35.61	1	52	buccal muco	1	1	0	4	4		0	0	0	2016/12/20	1	1	0
1	35.44	1	76	buccal muco	1	1		3	3		1	0	0	0000/00/00	0	2	1
1	35.38	1	69	lip	1	1	1	1	1	x	x		0	0000/00/00	0	1	
1	32.52	0	54	buccal muco	1	1	1	4	4		0	0	0	0000/00/00	0	2	0
1	12.39	0	44	rmt	1	1		4	1		2	1	0	2016/10/27	1	2	1
1	21.97	0	62	buccal muco	1	1	0	1	1	x	x		1	0000/00/00	0	2	
1	2.95	0	57	buccal muco	1	1	1	4	3		2	1	0	2016/9/6	1	3	1
1	35.18	1	47	ant tongue	1	1	1	2	2		0	0	0	0000/00/00	0	2	0
1	34.92	1	57	alveolar ridg	1	1	1	1	1		0	0	0	0000/00/00	0	2	0
1	34.82	1	48	ant tongue	1	1	1	4	1		2	1	0	0000/00/00	0	2	1
1	35.28	1	39	buccal muco	1	1		4	4		0	0	0	0000/00/00	0	1	0
1	34.82	1	65	alveolar ridg	1	1	1	4	4		0	0	0	0000/00/00	0	2	0
1	34.98	1	72	ant tongue	1	1	0	3	2		1	0	0	2016/10/26	1	2	1
1	35.05	1	53	buccal muco	1	1	1	1	1		0	0	0	0000/00/00	0	2	0
1	34.82	1	74	ant tongue	0	0	0	2	2		0	0	0	0000/00/00	0	3	0
1	58.82	1	61	hard palate	1	1	1	3	3		0	0	0	0000/00/00	0	2	0
1	35.74	1	64	buccal muco	1	0	1	1	1	x	x		0	0000/00/00	0	1	
1	19.70	0	47	rmt	1	1	1	1	1	x	x		0	2016/9/16	1	2	
1	34.72	1	70	alveolar ridg	1	1	0	4	4		0	0	0	0000/00/00	0	2	0
1	27.87	0	65	ant tongue	1	1		1	1		0	0	0	0000/00/00	0	2	0
1	12.13	0	83	ant tongue	1	0	0	2	2		0	0	0	0000/00/00	0	2	0
1	34.69	1	59	lip	1	1	0	2	2		0	0	0	0000/00/00	0	2	0
1	34.56	1	45	ant tongue	1	1	1	1	1	x	x		0	0000/00/00	0	2	
1	16.46	0	65	alveolar ridg	1	1	1	4	4		0	0	0	0000/00/00	0	3	0
1	36.36	1	62	lip	1	0	1	1	1		0	0	0	0000/00/00	0	1	0
1	34.46	1	55	ant tongue	1	1	1	1	1		0	0	0	0000/00/00	0	2	0
1	34.30	1	55	alveolar ridg	1	1	1	4	4		0	0	0	0000/00/00	0	2	0
1	36.20	1	58	rmt	1	1	1	4	4		0	0	0	0000/00/00	0	2	0
1	26.33	0	69	buccal muco	1	0		3	1		1	0	0	0000/00/00	0	2	1
1	35.15	1	73	buccal muco	1	1	1	1	1		0	0	0	0000/00/00	0	2	0

1	34.95	1	65	mouth floor	1	1	1	4	4	0	0	0	0000/00/00	0	2	0
1	25.44	0	52	lip	1	1		1	1	0	0	0	0000/00/00	0	2	0
1	34.10	1	58	buccal muco	1	0	1	4	4	0	0	0	0000/00/00	0	2	0
1	34.26	1	53	alveolar ridg	1	1	1	2	2	x	x	0	0000/00/00	0	2	
1	34.23	1	62	buccal muco	1	1		1	1	x	x	0	0000/00/00	0	2	
1	34.00	1	56	buccal muco	1	1	1	4	4	2	1	0	0000/00/00	0	2	1
1	9.48	0	83	ant tongue	1	1	1	2	2	0	0	0	0000/00/00	0	2	0
1	34.95	1	65	buccal muco	1	1		1	1	0	0	0	0000/00/00	0	1	0
1	34.75	1	46	ant tongue	1	0	0	2	2	0	0	0	0000/00/00	0	1	0
1	10.59	0	53	alveolar ridg	1	1	1	4	4	x	x	0	2017/2/15	1	2	
1	34.92	1	49	lip	1	1	0	1	1	0	0	0	0000/00/00	0	2	0
1	33.84	1	67	ant tongue	1	1	1	2	2	0	0	0	0000/00/00	0	2	0
1	35.34	1	62	buccal muco	1	1	1	1	1	x	x	0	0000/00/00	0	2	
1	33.77	1	32	ant tongue	1	1	1	1	1	0	0	0	0000/00/00	0	2	0
1	34.03	1	43	buccal muco	1	1		無法		0	x	0	2017/2/25	1	2	0
1	33.61	1	68	ant tongue	1	1		1	1	x	x	0	0000/00/00	0	2	
1	5.64	0	58	hard palate	1	0	0	4	4	0	0	0	0000/00/00	0	2	0
1	15.44	0	61	ant tongue	1	1	1	2	2	x	x	0	0000/00/00	0	2	
1	33.44	1	68	ant tongue	1	0	0	1	1	x	x	0	0000/00/00	0	2	
1	5.25	0	56	ant tongue	1	1	0	4	2	2	1	0	2016/11/9	1	2	1
1	33.21	1	65	alveolar ridg	1	1	1	4	4	1	0	0	0000/00/00	0	2	1
1	12.16	0	59	buccal muco	1	1	1	2	2	x	x	0	2017/3/20	1	2	
1	18.69	0	63	alveolar ridg	1	1	1	1	1	0	0	0	0000/00/00	0	2	0
1	33.28	1	59	hard palate	1	1	0	1	1	0	0	0	0000/00/00	0	2	0
1	33.84	1	58	ant tongue	0	1	0	1	1	x	x	0	0000/00/00	0	2	
1	8.75	0	74	lip	1	1		2	2	0	0	0	2016/11/30	1	3	0
1	33.25	1	54	mouth floor	1	1		1	1	x	x	0	0000/00/00	0	2	
1	25.21	0	46	lip	1	1	1	3	3	0	0	0	0000/00/00	0	2	0
1	13.11	0	67	ant tongue	1	1		2	2	x	x	0	0000/00/00	0	2	
1	16.10	0	65	alveolar ridg	1	1	0	4	4	2	0	0	2017/5/8	1	2	1
1	33.28	1	74	buccal muco	1	0	0	2	2	0	0	0	0000/00/00	0	2	0
1	35.57	1	61	lip	1	1	1	1	1	0	0	0	0000/00/00	0	2	0
1	32.75	1	40	alveolar ridg	1	1		4	4	0	0	0	0000/00/00	0	2	0
1	32.95	1	44	buccal muco	1	0	1	2	2	0	0	0	0000/00/00	0	2	0
1	32.75	1	56	buccal muco	1	1		4	4	0	0	0	0000/00/00	0	2	0
1	32.82	1	65	ant tongue	1	0	0	1	1	0	0	0	0000/00/00	0	2	0
1	19.87	0	47	rmt	1	1	1	4	4	x	x	1	0000/00/00	0	2	
1	32.82	1	49	rmt	1	1	1	2	2	0	0	0	0000/00/00	0	3	0
1	33.18	1	50	ant tongue	1	0		1	1	0	0	0	0000/00/00	0	2	0
1	32.72	1	58	buccal muco	1	1	1	1	1	0	0	0	0000/00/00	0	1	0
1	20.23	0	73	ant tongue	1	1	0	1	1	0	0	0	2017/5/9	1	1	0
1	11.54	0	67	buccal muco	1	1		3	2	1	0	0	2017/3/15	1	2	1
1	32.66	1	57	ant tongue	1	1	1	4	2	2	0	0	0000/00/00	0	2	1
1	32.36	1	60	ant tongue	1	1	1	2	2	0	0	0	0000/00/00	0	1	0
1	32.46	1	67	buccal muco	1	1	1	1	1	0	0	0	0000/00/00	0	2	0
1	32.26	1	62	hard palate	1	1	1	1	1	0	0	0	0000/00/00	0	2	0
1	33.67	1	70	lip	1	1	1	2	2	x	x	1	0000/00/00	0	2	
1	32.75	1	49	buccal muco	1	1	1	4	4	0	0	0	0000/00/00	0	2	0
1	32.36	1	68	buccal muco	1	0	1	1	1	x	x	0	0000/00/00	0	1	
1	32.16	1	56	buccal muco	1	1	1	無法		0	0	0	0000/00/00	0	2	0
1	32.23	1	61	ant tongue	1	1	0	1	1	0	0	0	0000/00/00	0	2	0
1	32.00	1	62	alveolar ridg	1	1	1	4	4	0	0	0	0000/00/00	0	2	0
1	32.69	1	48	hard palate	1	1	0	4	4	0	0	0	0000/00/00	0	2	0
1	15.25	0	56	lip	1	1	0	4	4	0	0	0	0000/00/00	0	2	0
1	31.93	1	36	buccal muco	1	1	0	1	1	0	0	0	0000/00/00	0	2	0
1	32.69	1	48	buccal muco	1	1	0	1	1	0	0	0	2018/1/7	1	2	0
1	31.93	1	54	buccal muco	1	1	1	3	3	0	0	0	0000/00/00	0	2	0
1	14.30	0	56	buccal muco	1	1	0	1	1	0	0	0	0000/00/00	0	2	0
1	32.00	1	47	buccal muco	1	1	0	2	2	0	0	0	0000/00/00	0	2	0
1	20.16	0	70	hard palate	1	1	1	2	2	x	x	0	0000/00/00	0	1	
1	31.80	1	58	ant tongue	1	1	0	2	2	0	0	0	0000/00/00	0	2	0
1	32.85	1	77	rmt	1	1	1	4	4	0	0	0	0000/00/00	0	2	0
1	31.80	1	43	buccal muco	1	1		4	2	2	1	0	0000/00/00	0	2	1
1	31.70	1	66	buccal muco	0	1	0	1	1	0	0	0	0000/00/00	0	2	0
1	31.70	1	62	lip	1	1	0	1	1	x	x	0	0000/00/00	0	1	
1	31.51	1	66	ant tongue	1	1	1	2	2	0	0	0	0000/00/00	0	1	0
1	31.57	1	50	alveolar ridg	1	1	1	2	2	0	0	0	0000/00/00	0	2	0
1	31.54	1	51	ant tongue	1	1	1	2	2	0	0	0	0000/00/00	0	2	0
1	31.38	1	51	buccal muco	1	1	1	1	1	x	x	0	0000/00/00	0	2	
1	31.34	1	46	ant tongue	1	1	1	1	1	x	x	0	0000/00/00	0	2	
1	31.34	1	62	ant tongue	1	1	1	1	1	0	0	0	0000/00/00	0	2	0
1	14.10	0	37	ant tongue	1	1	1	1	1	0	0	0	2017/1/25	1	3	0

1	8.26	0	55	alveolar ridg	1	1	1	4	4	x	x	1	2017/6/5	1	2	
1	33.87	1	43	buccal muco	1	1	1	3	3	0	0	0	0000/00/00	0	2	0
1	34.07	1	63	buccal muco	1	1	1	1	1	0	0	0	0000/00/00	0	2	0
1	31.28	1	52	ant tongue	1	1	1	3	3	0	0	0	0000/00/00	0	2	0
1	4.95	0	53	buccal muco	1	1	1	4	4	2	1	0	2017/3/16	1	2	1
1	31.05	1	56	buccal muco	1	1	1	4	4	0	0	0	0000/00/00	0	2	0
1	31.25	1	48	alveolar ridg	1	1	1	4	4	0	0	1	2018/2/2	1	2	0
1	31.08	1	36	ant tongue	1	1	1	4	4	1	0	0	0000/00/00	0	2	1
1	32.82	1	49	ant tongue	1	1	1	1	1	0	0	0	0000/00/00	0	2	0
1	30.85	1	56	alveolar ridg	1	1	1	1	1	x	x	0	0000/00/00	0	2	
1	8.43	0	48	ant tongue	1	1	1	4	4	2	1	0	2017/5/12	1	2	1
1	30.89	1	76	buccal muco	1	0	1	2	2	0	0	0	0000/00/00	0	2	0
1	30.79	1	50	ant tongue	1	0	0	1	1	x	x	0	0000/00/00	0	1	
1	31.84	1	59	rmt	1	1		3	1	1	1	0	0000/00/00	0	2	1
1	11.84	0	60	buccal muco	0	1	1	3	2	1	1	0	2017/5/24	1	2	1
1	30.69	1	44	lip	1	0	1	1	1	0	0	0	0000/00/00	0	1	0
1	30.66	1	53	buccal muco	1	1	1	4	4	0	0	0	0000/00/00	0	1	0
1	30.43	1	65	ant tongue	1	0	0	4	1	2	0	0	0000/00/00	0	2	1
1	10.82	0	67	alveolar ridg	1	1		1	1	x	x	0	2017/1/4	1	2	
1	9.44	0	73	buccal muco	1	1	0	2	2	x	x	0	2017/5/26	1	2	
1	30.39	1	81	alveolar ridg	1	0	0	4	4	0	0	0	0000/00/00	0	2	0
1	30.56	1	42	hard palate	1	1	1	1	1	x	x	0	0000/00/00	0	2	
1	30.79	1	54	buccal muco	1	1	0	1	1	0	0	0	0000/00/00	0	1	0
1	31.38	1	52	rmt	1	1	1	2	2	0	0	0	0000/00/00	0	1	0
1	30.56	1	59	ant tongue	1	1	1	4	4	0	0	0	0000/00/00	0	2	0
1	28.89	0	46	ant tongue	1	0	1	1	1	0	0	0	2017/5/15	1	2	0
1	30.66	1	53	alveolar ridg	1	1	1	3	3	x	x	0	0000/00/00	0	1	
1	30.39	1	51	buccal muco	1	1	1	1	1	x	x	0	0000/00/00	0	2	
1	30.20	1	53	hard palate	1	1	1	3	2	1	0	0	0000/00/00	0	3	1
1	14.26	0	57	ant tongue	1	1	1	無法	2	x	x	0	0000/00/00	0	3	
1	30.23	1	36	buccal muco	1	1	1	1	1	0	0	0	0000/00/00	0	2	0
1	30.46	1	58	buccal muco	1	1	1	3	1	1	0	0	0000/00/00	0	2	1
1	14.26	0	65	lip	1	1	1	2	2	x	x	0	2017/5/22	1	2	
1	30.30	1	45	buccal muco	1	1	1	1	1	x	x	0	0000/00/00	0	1	
1	6.16	0	56	hard palate	1	1		4	4	0	0	0	2017/5/26	1	2	0
1	30.10	1	42	ant tongue	1	1	1	1	1	0	0	0	2018/2/23	1	1	0
1	17.57	0	55	ant tongue	1	1	1	1	1	0	0	0	2017/8/11	1	2	0
1	30.00	1	53	alveolar ridg	1	0	1	2	2	0	0	0	0000/00/00	0	2	0
1	4.23	0	54	ant tongue	1	1	1	4	2	2	1	0	2017/2/16	1	3	1
1	29.93	1	40	buccal muco	1	1	1	4	1	2	1	0	0000/00/00	0	2	1
1	30.39	1	51	buccal muco	1	1	1	4	4	0	0	0	0000/00/00	0	1	0
1	29.74	1	52	ant tongue	1	1		2	2	0	0	0	0000/00/00	0	2	0
1	29.77	1	59	buccal muco	1	1	1	1	1	0	0	0	0000/00/00	0	1	0
1	30.10	1	75	lip	0	0	0	1	1	x	x	0	0000/00/00	0	2	
1	26.33	0	54	buccal muco	1	1		4	4	1	0	0	2017/7/7	1	2	1
1	10.10	0	57	alveolar ridg	1	0	0	4	4	2	1	0	2017/6/21	1	2	1
1	9.08	0	50	ant tongue	1	1		4	1	2	1	0	2017/8/9	1	2	1
1	29.64	1	69	alveolar ridg	1	1	1	2	2	0	0	0	2017/11/6	1	2	0
1	29.31	1	48	buccal muco	1	1		4	4	0	0	0	0000/00/00	0	2	0
1	17.41	0	83	buccal muco	1	1	1	1	1	x	x	0	0000/00/00	0	2	
1	29.54	1	63	ant tongue	1	1		1	1	0	0	0	0000/00/00	0	1	0
1	30.00	1	53	lip	1	1		1	1	x	x	0	0000/00/00	0	2	
1	29.31	1	57	rmt	1	1	1	1	1	0	0	0	0000/00/00	0	2	0
1	30.20	1	73	rmt	1	0	1	4	4	0	0	0	0000/00/00	0	1	0
1	29.41	1	56	mouth floor	1	1	1	1	1	x	x	0	0000/00/00	0	2	
1	14.98	0	67	ant tongue	1	1	1	4	4	1	0	1	2017/11/17	1	1	1
1	30.20	1	44	buccal muco	1	1	1	1	1	x	x	0	0000/00/00	0	1	
1	29.70	1	56	lip	1	1	0	1	1	x	x	0	0000/00/00	0	3	
1	5.15	0	74	alveolar ridg	1	1		1	1	0	0	0	0000/00/00	0	2	0
1	28.92	1	39	ant tongue	1	1		4	4	0	0	0	0000/00/00	0	2	0
1	29.48	1	64	buccal muco	1	1	1	1	1	x	x	0	0000/00/00	0	2	
1	20.26	0	59	alveolar ridg	1	1		1	1	x	x	0	0000/00/00	0	2	
1	29.05	1	72	ant tongue	1	0	0	2	2	0	0	0	0000/00/00	0	2	0
1	28.98	1	39	rmt	1	1	1	3	2	1	0	0	0000/00/00	0	2	1
1	19.74	0	36	buccal muco	1	1	1	3	3	0	0	0	2017/9/15	1	2	0
1	20.69	0	71	alveolar ridg	1	1	1	4	4	x	x	0	0000/00/00	0	2	
1	29.44	1	54	ant tongue	1	1	1	1	1	0	0	0	0000/00/00	0	2	0
1	28.72	1	50	ant tongue	1	1	1	1	1	x	x	0	0000/00/00	0	2	
1	28.72	1	78	buccal muco	1	1	1	2	2	0	0	0	0000/00/00	0	2	0
1	29.44	1	63	ant tongue	1	1		2	2	x	x	0	0000/00/00	0	2	
1	28.56	1	62	alveolar ridg	1	1	1	1	1	x	x	0	0000/00/00	0	2	
1	29.51	1	77	alveolar ridg	1	1	1	1	1	x	x	0	0000/00/00	0	2	

1	14.95	0	58	rmt	1	1		4	4	0	0	0	0000/00/00	0	2	0
1	25.08	0	72	ant tongue	1	1		1	1	x	x	0	0000/00/00	0	1	
1	28.52	1	59	buccal muco	1	0	0	4	2	2	1	0	0000/00/00	0	2	1
1	29.48	1	46	ant tongue	1	1	1	4	4	2	0	0	0000/00/00	0	2	1
1	28.85	1	64	ant tongue	1	1	1	3	1	1	0	0	0000/00/00	0	2	1
1	29.25	1	67	buccal muco	1	1	0	4	4	0	0	0	0000/00/00	0	2	0
1	28.89	1	56	alveolar ridge	1	0	0	4	4	0	0	0	0000/00/00	0	2	0
1	18.16	0	47	ant tongue	1	1	0	1	1	0	0	0	2017/7/8	1	3	0
1	28.30	1	60	alveolar ridge	1	1	1	4	4	0	0	0	0000/00/00	0	2	0
1	7.08	0	65	buccal muco	1	1	1	4	4	2	1	0	0000/00/00	0	2	1
1	28.23	1	68	buccal muco	1	1	1	1	1	x	x	0	0000/00/00	0	1	
1	28.10	1	80	buccal muco	1	1	1	1	1	x	x	0	0000/00/00	0	3	
1	28.13	1	37	buccal muco	1	1	1	4	1	2	1	0	0000/00/00	0	2	1
1	27.84	1	84	ant tongue	1	1	0	2	2	0	0	0	0000/00/00	0	3	0
1	27.87	1	52	buccal muco	1	1	1	4	4	0	0	0	0000/00/00	0	2	0
1	27.80	1	45	ant tongue	1	1	1	4	4	0	0	0	0000/00/00	0	2	0
1	61.05	1	61	rmt	1	1	0	無法	1	x	x	0	0000/00/00	0	1	
1	27.67	1	67	ant tongue	1	1	1	4	2	2	1	0	0000/00/00	0	2	1
1	28.79	1	59	lip	1	1	1	3	3	0	0	0	0000/00/00	0	2	0
1	28.85	1	46	alveolar ridge	1	1	1	4	4	2	1	0	0000/00/00	0	2	1
1	27.67	1	52	alveolar ridge	1	1	1	1	1	0	0	0	0000/00/00	0	2	0
1	28.43	1	42	ant tongue	1	1	1	1	1	0	0	0	2017/10/17	1	2	0
1	28.79	1	68	buccal muco	1	0	1	2	2	0	0	0	0000/00/00	0	1	0
1	27.54	1	67	rmt	1	1	1	2	2	0	0	0	0000/00/00	0	2	0
1	27.67	1	44	buccal muco	1	1	1	1	1	0	0	0	0000/00/00	0	2	0
1	28.56	1	65	lip	1	1	1	3	2	1	0	0	0000/00/00	0	2	1
1	10.85	0	52	ant tongue	1	0	1	4	4	2	1	0	2017/8/2	1	2	1
1	27.48	1	56	ant tongue	1	1	1	3	1	1	0	0	0000/00/00	0	2	1
1	28.13	1	62	hard palate	1	1		1	1	0	0	0	0000/00/00	0	1	0
1	27.38	1	56	buccal muco	1	0	1	4	4	0	0	0	0000/00/00	0	2	0
1	27.57	1	61	lip	1	1	1	1	1	0	0	0	0000/00/00	0	1	0
1	27.18	1	57	alveolar ridge	1	1	1	2	2	0	0	0	0000/00/00	0	2	0
1	8.92	0	75	alveolar ridge	1	1	0	1	1	x	x	0	2017/8/30	1	2	
1	29.93	1	63	buccal muco	1	1	1	1	1	0	0	0	2017/12/20	1	2	0
1	27.15	1	60	hard palate	1	1	1	3	1	1	0	0	0000/00/00	0	2	1
1	27.44	1	56	alveolar ridge	1	1		4	4	2	1	0	0000/00/00	0	2	1
1	27.11	1	46	lip	1	1	1	2	2	x	x	0	0000/00/00	0	1	
1	16.56	0	55	alveolar ridge	1	1	1	4	4	2	1	0	2017/7/17	1	3	1
1	27.41	1	48	alveolar ridge	1	1	1	4	4	0	0	0	2018/6/19	1	2	0
1	26.66	1	41	lip	1	1	1	1	1	x	x	0	0000/00/00	0	2	
1	26.66	1	56	buccal muco	1	1	0	4	4	0	0	0	0000/00/00	0	2	0
1	26.66	1	65	alveolar ridge	1	1	0	4	4	0	0	0	0000/00/00	0	2	0
1	26.52	1	53	buccal muco	1	1	1	1	1	0	0	0	0000/00/00	0	2	0
1	26.56	1	74	lip	1	1	1	2	2	0	0	0	0000/00/00	0	2	0
1	26.52	1	41	buccal muco	1	1	1	1	1	0	0	0	0000/00/00	0	2	0
1	8.82	0	40	ant tongue	0	1	1	4	4	3	1	0	2017/8/14	1	2	1
1	26.89	1	38	alveolar ridge	1	1	1	4	4	0	0	0	0000/00/00	0	2	0
1	26.95	1	60	buccal muco	0	1	1	4	4	0	0	0	0000/00/00	0	2	0
1	26.26	1	67	ant tongue	1	1	1	3	1	1	0	0	0000/00/00	0	3	1
1	26.49	1	57	lip	1	1	1	1	1	0	0	0	0000/00/00	0	2	0
1	26.43	1	65	ant tongue	1	0	1	1	1	0	0	0	0000/00/00	0	2	0
1	12.82	0	52	ant tongue	1	0		4	4	2	0	0	2018/2/3	1	2	1
1	26.26	1	31	ant tongue	0	0		1	1	x	x	0	0000/00/00	0	2	
1	26.49	1	56	ant tongue	0	0	0	1	1	0	0	0	0000/00/00	0	3	0
1	8.92	0	73	buccal muco	1	1	1	4	4	2	1	0	2017/8/14	1	2	1
1	26.56	1	66	buccal muco	1	1	1	4	4	2	1	0	0000/00/00	0	2	1
1	26.43	1	62	buccal muco	1	1	0	2	2	0	0	0	0000/00/00	0	1	0
1	26.30	1	74	hard palate	1	1	1	4	4	0	0	0	0000/00/00	0	2	0
1	26.23	1	74	rmt	1	1	1	1	1	0	0	0	0000/00/00	0	2	0
1	26.30	1	56	alveolar ridge	1	1	1	4	4	2	1	0	0000/00/00	0	2	1
1	9.44	0	44	buccal muco	1	1	1	4	4	0	0	0	0000/00/00	0	2	0
1	1.54	0	66	hard palate	0	0	0	1	1	0	0	0	0000/00/00	0	1	0
1	26.39	1	60	ant tongue	1	1	1	1	1	0	0	0	0000/00/00	0	2	0
1	26.03	1	39	ant tongue	1	1	1	4	4	0	0	0	2018/1/18	1	2	0
1	25.97	1	67	alveolar ridge	0	1		4	4	0	0	0	0000/00/00	0	1	0
1	26.26	1	60	buccal muco	1	1	1	4	4	0	0	0	0000/00/00	0	2	0
1	25.97	1	33	ant tongue	1	1	1	1	1	0	0	0	0000/00/00	0	3	0
1	25.97	1	56	buccal muco	1	1	1	4	4	1	1	0	0000/00/00	0	3	1
1	26.43	1	37	buccal muco	1	0		1	1	0	0	0	2017/7/14	1	1	0
1	26.75	1	53	buccal muco	1	1	0	2	2	0	0	0	0000/00/00	0	1	0
1	18.00	0	53	buccal muco	1	1	1	3	3	0	0	1	0000/00/00	0	2	0
1	26.07	1	54	lip	1	1	1	無法	1	x	x	0	0000/00/00	0	1	

1	25.74	1	50	mouth floor	1	1	1		4	4		0		0	0	0000/00/00	0	2	0
1	25.77	1	51	ant tongue	1	1	1		1	1	x		x		0	0000/00/00	0	2	
1	26.49	1	57	buccal muco	1	1	1		1	1		0		0	0	0000/00/00	0	2	0
1	25.90	1	53	buccal muco	1	1	1		4	2		2		1	0	0000/00/00	0	2	1
1	26.20	1	55	rmt	1	1	1		2	2		0		0	0	0000/00/00	0	2	0
1	25.97	1	42	alveolar ridg	1	1	0		3	2		1		0	0	0000/00/00	0	3	1
1	26.43	1	65	ant tongue	1	1	0		1	1	x		x		0	0000/00/00	0	1	
1	25.34	1	52	buccal muco	1	1			4	4		0		0	0	0000/00/00	0	2	0
1	27.34	1	56	ant tongue	1	1			4	4		0		0	0	0000/00/00	0	2	0
1	10.66	0	49	ant tongue	0	0	1		4	4		0		0	1	2018/1/13	1	3	0
1	25.31	1	65	rmt	1	1			1	1	x		x		0	0000/00/00	0	2	
1	45.25	1	63	lip	1	1	1		1	1	x		x		0	0000/00/00	0	2	
1	10.98	0	53	buccal muco	1	1	1		4	3		2		0	0	2017/10/3	1	2	1
1	24.92	1	60	alveolar ridg	1	1	1		1	1		0		0	0	0000/00/00	0	1	0
1	25.74	1	65	buccal muco	1	1	1		3	3		0		0	0	0000/00/00	0	2	0
1	48.95	1	55	buccal muco	1	0	0		1	1	x		x		0	0000/00/00	0	2	
1	36.85	1	36	ant tongue	1	1	1		3	1		1		0	0	0000/00/00	0	2	1
1	22.07	0	63	buccal muco	1	1			1	1		0		0	0	0000/00/00	0	2	0
1	25.97	1	55	buccal muco	1	1			4	1		2		0	0	0000/00/00	0	2	1
1	24.66	1	54	rmt	1	1	1		4	4	x		x		0	0000/00/00	0	2	
1	25.61	1	52	buccal muco	1	1	1		4	3		2		1	0	0000/00/00	0	2	1
1	13.11	0	58	buccal muco	1	1	1		4	4		1		1	0	2018/4/19	1	2	1
1	25.02	1	47	ant tongue	1	1	0		1	1		0		0	0	0000/00/00	0	2	0
1	24.56	1	67	buccal muco	1	1	1		2	2		0		0	0	0000/00/00	0	2	0
1	25.08	1	70	lip	1	1			1	1	x		x		0	0000/00/00	0	2	
1	24.66	1	64	ant tongue	1	1	1		2	2		0		0	0	0000/00/00	0	2	0
1	24.59	1	55	buccal muco	1	1	1		1	1	x		x		0	2018/4/26	1	1	
1	8.33	0	51	alveolar ridg	1	1	1		4	4		0		0	0	0000/00/00	0	2	0
1	24.49	1	65	buccal muco	1	1	1		4	4		0		0	0	0000/00/00	0	2	0
1	8.00	0	64	alveolar ridg	1	1			4	4		2		1	0	0000/00/00	0	1	1
1	24.39	1	55	alveolar ridg	1	1	1		1	1	x		x		0	0000/00/00	0	2	
1	24.43	1	39	buccal muco	1	1	0		3	1		1		0	0	0000/00/00	0	2	1
1	24.36	1	48	alveolar ridg	1	1			4	4		0		0	0	0000/00/00	0	2	0
1	14.23	0	67	buccal muco	0	0	1		4	4	x		x		0	2016/2/24	1	2	
1	9.67	0	76	buccal muco	1	1			1	1		0		0	0	0000/00/00	0	2	0
1	26.49	1	42	rmt	1	0			4	4		0		0	0	0000/00/00	0	2	0
1	24.20	0	71	buccal muco	1	1	0		1	1	x		x		0	2018/4/25	1	2	
1	24.23	1	60	ant tongue	1	1			1	1	x		x		0	0000/00/00	0	2	
1	24.13	1	61	mouth floor	1	1	1		1	1		0		0	0	0000/00/00	0	2	0
1	24.10	1	65	ant tongue	1	0	1		2	2		0		0	0	0000/00/00	0	2	0
1	24.43	1	48	ant tongue	1	1			4	4		0		0	0	0000/00/00	0	2	0
1	24.13	1	51	buccal muco	1	1	1		4	4		0		0	0	0000/00/00	0	2	0
1	6.30	0	56	ant tongue	1	1	0		2	2	x		x		0	2017/11/22	1	2	
1	10.92	0	65	alveolar ridg	1	1			4	4		0		0	0	2018/4/7	1	2	0
1	23.90	1	63	lip	1	1	1		1	1	x		x		0	0000/00/00	0	1	
1	23.93	1	52	mouth floor	1	1	1		1	1		0		0	0	0000/00/00	0	2	0
1	23.97	1	64	buccal muco	1	1	1		1	1		0		0	0	2018/2/27	1	1	0
1	23.90	1	57	ant tongue	1	1	1		3	1		1		0	0	0000/00/00	0	2	1
1	23.77	1	59	ant tongue	1	1	1		4	4		1		1	0	0000/00/00	0	2	1
1	23.77	1	58	hard palate	1	1	1		4	1		2		1	1	0000/00/00	0	2	1
1	23.80	1	40	ant tongue	1	1	1		1	1		0		0	0	0000/00/00	0	2	0
1	23.67	1	65	mouth floor	1	1	0		1	1	x		x		0	0000/00/00	0	2	
1	23.54	1	51	buccal muco	1	1	1		2	2		0		0	0	0000/00/00	0	2	0
1	23.74	1	56	buccal muco	1	1	0		3	3		0		0	0	0000/00/00	0	1	0
1	7.90	0	51	alveolar ridg	1	1	1		2	2	x		x		0	0000/00/00	0	3	
1	23.51	1	73	lip	1	1			1	1		0		0	0	0000/00/00	0	2	0
1	23.48	1	80	hard palate	1	1	1		1	1		0		0	0	0000/00/00	0	2	0
1	23.80	1	80	buccal muco	1	1	1		1	1		0		0	0	0000/00/00	0	2	0
1	34.92	1	63	buccal muco	1	1	1		1	1	x		x		0	0000/00/00	0	2	
1	34.79	1	57	buccal muco	1	1	1		2	2		0		0	0	0000/00/00	0	2	0
1	7.08	0	76	buccal muco	1	1	1	無法		1	x		x		0	0000/00/00	0	2	
1	23.48	1	55	mouth floor	1	1	1		2	2		0		0	0	0000/00/00	0	2	0
1	23.54	1	46	alveolar ridg	1	1		無法				0	x		0	0000/00/00	0	2	0
1	23.57	1	66	ant tongue	1	1	1		3	1		1		0	0	0000/00/00	0	2	1
1	6.89	0	39	ant tongue	1	1	1		3	1		1		1	0	0000/00/00	0	1	1
1	14.52	0	56	buccal muco	1	1	1		3	2		1		0	0	2018/2/2	1	2	1
1	23.74	1	48	buccal muco	1	1	1		2	2		0		0	0	0000/00/00	0	2	0
1	23.48	1	62	ant tongue	0	0	0		1	1		0		0	0	0000/00/00	0	2	0
1	23.08	1	68	ant tongue	1	0	0	無法		1	x		x		0	0000/00/00	0	2	
1	23.11	1	53	buccal muco	1	1	1		1	1		0		0	0	0000/00/00	0	2	0
1	23.11	1	63	buccal muco	1	1	0		4	1		2		1	0	0000/00/00	0	3	1
1	23.08	1	57	ant tongue	1	1			3	3		1		0	0	0000/00/00	0	2	1

1	6.10	0	50	buccal muco	1	0	1	4	4	2	0	0	0000/00/00	0	2	1
1	34.13	1	55	buccal muco	1	1	1	1	1	0	0	0	0000/00/00	0	2	0
1	34.13	1	55	alveolar ridg	1	1	1	1	1	0	0	0	0000/00/00	0	2	0
1	22.82	1	69	lip	1	1	1	1	1	0	0	0	0000/00/00	0	2	0
1	22.98	1	62	buccal muco	1	1	1	4	1	2	1	0	0000/00/00	0	2	1
1	10.59	0	66	alveolar ridg	1	0	0	1	1	x	x	0	0000/00/00	0	2	
1	22.79	1	52	ant tongue	1	1	1	1	1	0	0	0	0000/00/00	0	2	0
1	23.21	1	64	buccal muco	1	1	1	3	2	1	1	0	0000/00/00	0	2	1
1	90.59	0	55	alveolar ridg	1	1		4	4	x	x	0	0000/00/00	0	2	
1	22.52	1	58	ant tongue	1	1	1	3	1	1	1	0	0000/00/00	0	2	1
1	22.59	1	47	buccal muco	1	1	1	1	1	x	x	0	0000/00/00	0	1	
1	12.20	0	59	ant tongue	1	1	1	1	1	x	x	0	2017/3/20	1	2	
1	22.85	1	54	buccal muco	1	1	1	4	4	0	0	0	0000/00/00	0	2	0
1	22.39	1	42	buccal muco	1	1		2	2	x	x	0	0000/00/00	0	1	
1	22.30	1	58	ant tongue	1	0	0	3	3	0	0	0	0000/00/00	0	2	0
1	22.13	1	52	ant tongue	1	1	1	1	1	0	0	0	0000/00/00	0	2	0
1	23.31	1	78	lip	0	0		2	2	0	0	0	0000/00/00	0	2	0
1	20.98	0	58	alveolar ridg	1	1		4	4	0	0	0	0000/00/00	0	2	0
1	22.16	1	64	buccal muco	1	1		2	2	0	0	0	0000/00/00	0	1	0
1	9.87	0	53	ant tongue	1	1	1	3	2	1	0	0	2018/2/14	1	3	1
1	9.11	0	57	rmt	1	1	1	4	4	2	1	0	2018/1/12	1	2	1
1	23.93	1	57	alveolar ridg	1	1	1	1	1	0	0	0	0000/00/00	0	2	0
1	22.00	1	55	lip	1	1		3	2	1	0	0	2018/4/24	1	2	1
1	12.07	0	62	alveolar ridg	1	1	1	4	4	0	0	1	2018/3/17	1	1	0
1	21.90	1	58	alveolar ridg	1	1	1	4	4	x	x	0	0000/00/00	0	2	
1	22.10	1	62	ant tongue	1	0	1	4	2	2	0	0	0000/00/00	0	2	1
1	22.39	1	49	buccal muco	1	1	1	4	4	0	0	0	0000/00/00	0	2	0
1	21.90	1	63	ant tongue	1	1	1	4	2	2	1	0	0000/00/00	0	2	1
1	21.90	1	60	buccal muco	1	1	1	4	4	0	0	0	0000/00/00	0	2	0
1	21.08	0	57	buccal muco	1	1	1	4	3	2	1	0	2018/2/9	1	2	1
1	21.74	1	47	mouth floor	1	1	1	2	2	0	0	0	0000/00/00	0	2	0
1	21.74	1	51	rmt	1	1	1	2	2	0	0	0	0000/00/00	0	2	0
1	21.80	1	65	buccal muco	1	1	1	1	1	x	x	0	0000/00/00	0	1	
1	22.30	1	62	lip	1	1	1	1	1	x	x	0	0000/00/00	0	1	
1	21.61	1	68	buccal muco	1	1	0	1	1	0	0	0	0000/00/00	0	1	0
1	22.07	1	47	buccal muco	1	1	1	3	3	0	0	0	0000/00/00	0	1	0
1	16.62	0	47	ant tongue	1	1	1	2	2	0	0	0	0000/00/00	0	2	0
1	14.66	0	55	buccal muco	1	1	1	4	4	2	0	0	2018/3/7	1	2	1
1	21.93	1	85	alveolar ridg	1	1		4	4	0	0	0	0000/00/00	0	2	0
1	22.43	1	64	lip	1	1	1	2	2	x	x	0	0000/00/00	0	2	
1	21.61	1	64	buccal muco	1	1	1	1	1	0	0	0	0000/00/00	0	2	0
1	21.48	1	70	buccal muco	1	1	0	1	1	x	x	0	0000/00/00	0	2	
1	21.51	1	57	lip	1	1		1	1	0	0	0	0000/00/00	0	2	0
1	21.38	1	45	alveolar ridg	1	1	1	3	1	1	0	0	0000/00/00	0	3	1
1	21.44	1	57	lip	1	1		1	1	0	0	0	0000/00/00	0	1	0
1	21.28	1	58	buccal muco	1	1	0	4	3	3	1	0	0000/00/00	0	2	1
1	21.34	1	65	mouth floor	1	1	1	4	1	2	0	0	0000/00/00	0	2	1
1	21.44	1	46	alveolar ridg	1	1	1	1	1	x	x	0	0000/00/00	0	2	
1	21.28	1	70	buccal muco	1	1	1	1	1	x	x	0	0000/00/00	0	1	
1	6.98	0	47	ant tongue	1	1	1	4	4	2	0	0	2017/12/28	1	3	1
1	21.18	1	68	lip	1	1	0	1	1	0	0	0	0000/00/00	0	1	0
1	20.92	1	53	buccal muco	1	1		1	1	x	x	0	0000/00/00	0	2	
1	20.92	1	63	buccal muco	1	1	1	1	1	x	x	0	0000/00/00	0	1	
1	20.82	1	38	ant tongue	1	1	1	4	4	0	0	0	0000/00/00	0	2	0
1	20.75	1	53	rmt	1	1	1	2	2	x	x	0	2018/1/4	1	2	
1	20.66	1	67	ant tongue	1	1		1	1	x	x	0	0000/00/00	0	2	
1	20.69	1	85	buccal muco	1	1		1	1	x	x	0	2018/6/4	1	2	
1	20.66	1	62	buccal muco	1	1	1	1	1	x	x	0	0000/00/00	0	2	
1	21.61	1	46	ant tongue	1	1	1	1	1	0	0	0	0000/00/00	0	2	0
1	9.48	0	68	alveolar ridg	1	1		4	4	2	1	0	0000/00/00	0	2	1
1	21.25	1	51	ant tongue	1	1		1	1	0	0	0	0000/00/00	0	2	0
1	20.66	1	58	buccal muco	1	1	1	3	1	1	0	0	0000/00/00	0	2	1
1	20.52	1	65	buccal muco	1	1	1	2	2	0	0	0	0000/00/00	0	2	0
1	20.69	1	73	buccal muco	1	1		4	4	0	0	0	0000/00/00	0	2	0
1	21.28	1	39	ant tongue	1	1	1	2	2	0	0	0	0000/00/00	0	2	0
1	20.92	1	60	alveolar ridg	1	1		1	1	x	x	0	0000/00/00	0	2	
1	14.36	0	56	ant tongue	1	1		4	4	3	1	0	2018/2/20	1	3	1
1	20.43	1	38	ant tongue	1	0		4	4	2	1	0	0000/00/00	0	2	1
1	20.26	1	47	mouth floor	1	1		3	2	1	1	0	0000/00/00	0	2	1
1	6.95	0	38	ant tongue	1	1	1	4	4	1	0	0	2018/1/18	1	2	1
1	10.10	0	47	rmt	1	1	1	4	4	2	1	0	2018/5/30	1	2	1
1	20.10	1	70	alveolar ridg	0	0	0	4	4	2	0	0	0000/00/00	0	2	1

1	20.33	1	50	alveolar ridg	1	1		1	1	0	0	0	0000/00/00	0	2	0
1	5.93	0	62	buccal muco	1	0		2	2	x	x	0	0000/00/00	0	2	
1	20.00	1	81	alveolar ridg	1	1	1	4	4	0	0	0	0000/00/00	0	2	0
1	19.90	1	45	ant tongue	1	1	1	1	1	x	x	0	0000/00/00	0	2	
1	19.90	1	37	alveolar ridg	1	1	1	1	1	x	x	0	0000/00/00	0	2	
1	19.87	1	46	ant tongue	1	1	0	3	3	0	0	0	0000/00/00	0	2	0
1	20.46	1	89	ant tongue	1	1	0	2	2	0	0	0	0000/00/00	0	2	0
1	12.89	0	41	buccal muco	1	1	1	1	1	x	x	0	2018/1/17	1	2	
1	19.38	1	68	mouth floor	1	1		2	2	x	x	0	0000/00/00	0	2	
1	19.61	1	51	ant tongue	1	0	1	1	1	0	0	0	0000/00/00	0	1	0
1	19.57	1	67	lip	1	1	1	2	2	0	0	0	0000/00/00	0	1	0
1	19.34	1	67	ant tongue	1	1		1	1	x	x	0	0000/00/00	0	2	
1	19.08	1	55	alveolar ridg	1	1		4	4	0	0	0	0000/00/00	0	2	0
1	19.08	1	82	lip	1	1	1	1	1	x	x	0	0000/00/00	0	2	
1	20.69	1	69	lip	1	1		1	1	0	0	0	0000/00/00	0	2	0
1	4.39	0	54	ant tongue	1	1	0	4	4	2	1	0	2018/2/22	1	2	1
1	18.89	1	54	lip	1	0	0	4	1	2	0	0	0000/00/00	0	2	1
1	18.98	1	49	alveolar ridg	1	1	0	4	4	0	0	0	0000/00/00	0	1	0
1	18.95	1	43	buccal muco	1	1	1	1	1	0	0	0	0000/00/00	0	2	0
1	20.46	1	68	ant tongue	1	1	1	1	1	x	x	0	0000/00/00	0	2	
1	18.75	1	74	buccal muco	1	1	1	1	1	x	x	0	0000/00/00	0	2	
1	18.89	1	47	ant tongue	1	0		1	1	0	0	0	0000/00/00	0	2	0
1	19.61	1	86	buccal muco	1	1	0	1	1	x	x	0	2018/9/7	1	1	
1	10.62	0	66	ant tongue	1	1		4	4	2	0	0	0000/00/00	0	2	1
1	18.62	1	69	ant tongue	1	0		4	3	2	1	0	0000/00/00	0	2	1
1	18.49	1	62	alveolar ridg	1	1	1	4	4	0	0	0	0000/00/00	0	2	0
1	18.89	1	53	buccal muco	1	1	1	3	3	0	0	0	0000/00/00	0	2	0
1	18.43	1	52	lip	1	1	1	1	1	x	x	0	0000/00/00	0	1	
1	19.18	1	58	ant tongue	1	1	1	1	1	0	0	0	2018/8/27	1	2	0
1	18.49	1	48	mouth floor	1	1	1	2	2	0	0	0	0000/00/00	0	2	0
1	19.15	1	54	rmt	1	1		2	2	0	0	0	0000/00/00	0	2	0
1	19.08	1	73	hard palate	1	1	1	4	4	x	x	0	0000/00/00	0	2	
1	18.39	1	72	buccal muco	1	1	1	1	1	x	x	0	0000/00/00	0	1	
1	18.16	1	70	alveolar ridg	0	0	1	2	2	0	0	0	0000/00/00	0	2	0
1	18.13	1	64	buccal muco	1	1	1	1	1	x	x	0	0000/00/00	0	2	
1	18.13	1	66	ant tongue	1	0	0	2	2	0	0	0	0000/00/00	0	2	0
1	18.49	1	59	buccal muco	1	1	1	4	4	1	0	0	0000/00/00	0	2	1
1	17.93	1	35	ant tongue	1	1	1	2	2	0	0	0	0000/00/00	0	3	0
1	17.74	1	65	alveolar ridg	1	1	1	4	4	0	0	0	0000/00/00	0	2	0
1	18.46	1	59	lip	1	1		1	1	x	x	0	0000/00/00	0	2	
1	17.84	1	72	buccal muco	0	1	1	2	2	0	0	0	0000/00/00	0	2	0
1	18.16	1	62	alveolar ridg	1	1	1	1	1	0	0	0	0000/00/00	0	2	0
1	18.00	1	43	alveolar ridg	1	1	1	4	4	0	0	0	0000/00/00	0	2	0
1	17.74	1	70	buccal muco	1	1		2	2	0	0	0	0000/00/00	0	2	0
1	17.61	1	50	lip	1	1	1	2	2	0	0	0	0000/00/00	0	2	0
1	19.57	1	55	buccal muco	1	1	0	1	1	0	0	0	0000/00/00	0	1	0
1	17.54	1	81	ant tongue	1	0	1	1	1	0	0	0	0000/00/00	0	2	0
1	17.57	1	53	ant tongue	1	1	1	1	1	0	0	0	0000/00/00	0	2	0
1	18.72	1	56	alveolar ridg	1	1		4	4	2	0	0	0000/00/00	0	2	1
1	6.98	0	59	alveolar ridg	1	1	1	4	4	2	0	0	2018/6/12	1	2	1
1	21.48	1	60	ant tongue	1	1	1	1	1	0	0	0	0000/00/00	0	2	0
1	17.38	1	57	buccal muco	1	1		4	4	1	1	0	0000/00/00	0	3	1
1	9.25	0	39	ant tongue	1	1	1	4	2	2	1	0	2018/6/1	1	2	1
1	17.38	1	77	buccal muco	1	1	0	2	2	0	0	0	0000/00/00	0	2	0
1	27.61	1	63	lip	1	1		1	1	0	0	0	0000/00/00	0	1	0
1	17.34	1	58	buccal muco	1	1	1	4	2	2	0	0	0000/00/00	0	2	1
1	17.51	1	58	mouth floor	1	1		1	1	0	0	0	0000/00/00	0	3	0
1	18.26	1	66	lip	0	0	0	1	1	0	0	0	0000/00/00	0	2	0
1	17.08	1	57	mouth floor	1	1		4	4	0	0	0	0000/00/00	0	2	0
1	17.15	1	61	alveolar ridg	1	1	1	4	4	0	0	0	0000/00/00	0	2	0
1	0.92	0	45	ant tongue	1	1	1	4	4	2	0	0	0000/00/00	0	2	1
1	17.08	1	66	buccal muco	1	1	1	1	1	0	0	0	0000/00/00	0	2	0
1	17.02	1	62	hard palate	1	1	1	1	1	x	x	0	0000/00/00	0	2	
1	17.02	1	55	rmt	1	1		4	3	2	0	0	0000/00/00	0	2	1
1	18.16	1	46	alveolar ridg	1	1	1	4	1	2	0	0	0000/00/00	0	2	1
1	17.38	1	52	rmt	1	1		4	4	0	0	0	0000/00/00	0	3	0
1	17.61	1	57	hard palate	1	1		4	4	x	x	0	0000/00/00	0	1	
1	16.82	1	52	mouth floor	1	1	1	2	2	0	0	0	0000/00/00	0	2	0
1	11.70	0	65	alveolar ridg	1	1	1	2	2	0	0	0	2018/5/3	1	2	0
1	16.79	1	69	buccal muco	1	1	1	3	2	1	0	0	2018/10/19	1	2	1
1	17.70	1	51	ant tongue	1	1		4	4	0	0	0	0000/00/00	0	2	0
1	16.69	1	59	buccal muco	1	1	0	1	1	0	0	0	0000/00/00	0	1	0

1	16.89	1	43	alveolar ridge	1	1	1	4	4	0	0	0	0000/00/00	0	2	0
1	16.82	1	44	rmt	1	1		4	1	2	0	0	2019/2/15	1	2	1
1	16.46	1	51	alveolar ridge	1	0	1	4	4	0	0	0	0000/00/00	0	2	0
1	16.82	1	68	alveolar ridge	1	1	1	4	4	0	0	0	0000/00/00	0	2	0
1	16.43	1	33	buccal mucosa				2	2	0	0	0	0000/00/00	0	3	0
1	16.33	1	69	hard palate	1	1	0	1	1	x	x	0	0000/00/00	0	2	
1	8.49	0	42	ant tongue	1	1	1	4	3	3	1	0	2018/6/5	1	3	1
1	16.16	1	68	lip	1	1		1	1	0	0	0	0000/00/00	0	2	0
1	16.82	1	57	buccal muco	1	1		1	1	0	0	0	0000/00/00	0	2	0
1	16.33	1	64	alveolar ridge	1	1	1	1	1	x	x	0	0000/00/00	0	2	
1	15.90	1	70	ant tongue				1	1	0	0	0	2018/10/1	1	2	0
1	16.13	1	57	ant tongue				2	2	0	0	0	0000/00/00	0	2	0
1	16.10	1	79	buccal muco	1	1	1	2	2	0	0	0	2018/5/25	1	2	0
1	16.10	1	56	ant tongue	1	1	0	3	3	0	0	0	0000/00/00	0	1	0
1	16.36	1	70	buccal muco	1	1	0	2	2	x	x	0	0000/00/00	0	2	
1	16.59	1	48	alveolar ridge	1	1	1	4	4	0	0	0	0000/00/00	0	2	0
1	15.70	1	63	buccal mucosa				4	4	0	0	0	0000/00/00	0	2	0
1	13.93	0	47	alveolar ridge	1	1	1	4	4	x	x	0	2018/10/8	1	2	
1	15.51	1	56	ant tongue				1	1	0	0	1	0000/00/00	0	1	0
1	15.54	1	59	lip	1	1	0	1	1	x	x	0	0000/00/00	0	2	
1	15.77	1	67	lip	1	1	0	1	1	0	0	0	0000/00/00	0	2	0
1	15.64	1	53	buccal muco	1	1	1	1	1	0	0	0	0000/00/00	0	2	0
1	16.00	1	57	ant tongue	1	0	1	3	2	1	0	0	0000/00/00	0	2	1
1	15.93	1	78	ant tongue	1	1		4	3	3	1	0	0000/00/00	0	2	1
1	6.56	0	69	alveolar ridge				4	4	0	0	0	0000/00/00	0	1	0
1	15.25	1	62	buccal mucosa				3	3	0	0	0	0000/00/00	0	2	0
1	15.28	1	46	ant tongue	1	1		4	3	3	1	0	0000/00/00	0	2	1
1	15.48	1	68	mouth floor	1	0	0	無法		0	x	0	0000/00/00	0	2	0
1	15.44	1	49	rmt	1	1		3	3	0	0	0	0000/00/00	0	2	0
1	15.25	1	54	lip	1	1	1	1	1	x	x	0	0000/00/00	0	2	
1	15.18	1	55	alveolar ridge	1	1	1	1	1	0	0	0	0000/00/00	0	2	0
1	15.08	1	47	mouth floor	1	1	1	2	2	0	0	0	0000/00/00	0	2	0
1	15.77	1	59	alveolar ridge	1	1	1	1	1	x	x	0	0000/00/00	0	2	
1	14.98	1	50	ant tongue	1	1	1	1	1	0	0	0	0000/00/00	0	2	0
1	13.77	0	74	buccal muco	1	1	1	1	1	0	0	0	0000/00/00	0	2	0
1	16.07	1	42	buccal muco	1	1	0	2	2	0	0	0	0000/00/00	0	1	0
1	15.08	1	57	ant tongue				3	3	0	0	0	0000/00/00	0	2	0
1	14.98	1	58	buccal muco	1	1	0	1	1	0	0	0	0000/00/00	0	1	0
1	14.92	1	66	alveolar ridge	1	1		4	4	0	0	0	0000/00/00	0	2	0
1	14.98	1	59	alveolar ridge	1	1	1	1	1	x	x	0	2018/6/23	1	1	
1	15.93	1	58	buccal muco	1	1	1	2	2	0	0	0	0000/00/00	0	1	0
1	15.25	1	50	alveolar ridge	1	1	1	4	4	2	1	0	0000/00/00	0	2	1
1	14.72	1	57	buccal muco	1	1	1	4	4	0	0	0	0000/00/00	0	2	0
1	14.62	1	57	lip	1	1		1	1	x	x	0	0000/00/00	0	2	
1	14.72	1	74	buccal muco	1	1	1	1	1	x	x	0	0000/00/00	0	2	
1	14.72	0	56	ant tongue	1	1	1	3	3	1	0	0	0000/00/00	0	2	1
1	14.59	1	68	alveolar ridge	1	1	1	4	4	3	0	0	0000/00/00	0	2	1
1	14.79	1	45	buccal muco	1	1	1	1	1	x	x	0	0000/00/00	0	2	
1	14.56	1	65	rmt				4	4	0	0	0	0000/00/00	0	2	0
1	14.59	1	38	buccal muco	0	0	1	4	2	3	0	0	0000/00/00	0	2	1
1	14.52	1	53	alveolar ridge	1	1	1	1	1	x	x	0	0000/00/00	0	2	
1	16.59	1	62	alveolar ridge	1	1	1	4	4	0	0	0	0000/00/00	0	1	0
1	8.56	0	58	ant tongue	1	1		1	1	x	x	0	0000/00/00	0	2	
1	14.49	1	43	ant tongue	1	1		1	1	0	0	0	0000/00/00	0	2	0
1	11.08	0	79	alveolar ridge	1	1	1	4	4	x	x	0	0000/00/00	0	2	
1	6.26	0	73	alveolar ridge	1	1	1	4	4	x	x	0	2018/6/20	1	2	
1	14.46	1	50	ant tongue	1	1		4	2	2	0	0	0000/00/00	0	2	1
1	14.26	1	58	alveolar ridge			1	2	2	x	x	0	2018/7/17	1	3	
1	14.39	1	54	buccal muco	1	1		1	1	0	0	0	0000/00/00	0	2	0
1	14.07	1	44	buccal mucosa				3	3	0	0	0	0000/00/00	0	2	0
1	14.30	1	63	buccal muco	1	1	0	1	1	0	0	0	0000/00/00	0	2	0
1	10.89	0	63	lip	1	0	0	1	1	0	0	0	0000/00/00	0	3	0
1	78.07	1	66	hard palate	1	1	0	1	1	0	0	0	0000/00/00	0	1	0
1	14.36	1	62	ant tongue	1	1	0	4	3	3	1	0	0000/00/00	0	2	1
1	14.82	1	54	buccal muco	1	1	1	3	3	0	0	0	0000/00/00	0	1	0
1	14.10	1	63	buccal mucosa				2	2	0	0	0	0000/00/00	0	2	0
1	16.52	1	70	alveolar ridge	1	1	1	4	4	0	0	0	0000/00/00	0	2	0
1	3.31	0	53	buccal muco	1	1	0	4	2	3	0	0	2018/6/19	1	2	1
1	14.56	1	55	buccal muco	1	1	0	4	4	0	0	0	0000/00/00	0	2	0
1	13.80	1	45	buccal muco	1	1	0	4	4	0	0	0	0000/00/00	0	2	0
1	14.52	1	69	buccal muco	0	1	1	3	3	x	x	0	0000/00/00	0	2	
1	13.97	1	78	ant tongue	1	1	1	3	3	0	0	0	0000/00/00	0	2	0

1	13.61	1	33	ant tongue	1	1	1	1	1	0	0	0	0000/00/00	0	2	0
1	13.44	1	66	buccal muco	1	1	0	2	2	0	0	0	2018/9/3	1	2	0
1	13.44	1	66	buccal muco	1	1	0	1	1	x	x	0	0000/00/00	0	2	
1	13.64	1	61	buccal muco	1	1	0	2	2	0	0	0	2018/7/21	1	2	0
1	15.08	1	63	buccal muco	1	0	0	1	1	0	0	0	0000/00/00	0	1	0
1	13.77	1	52	buccal muco	1	1	0	4	4	0	0	0	0000/00/00	0	2	0
1	26.20	1	55	lip	1	1	1	1	1	0	0	0	0000/00/00	0	2	0
1	13.48	1	48	buccal muco	1	1	1	1	1	x	x	0	0000/00/00	0	2	
1	13.41	1	72	alveolar ridg	1	1	0	1	1	0	0	0	0000/00/00	0	2	0
1	19.41	1	43	alveolar ridg	1	1	0	4	4	0	0	0	0000/00/00	0	2	0
1	13.87	1	66	ant tongue	1	1	1	2	2	x	x	0	0000/00/00	0	2	
1	13.38	1	73	buccal muco	0	1	0	2	2	0	0	0	0000/00/00	0	3	0
1	13.51	1	57	buccal mucosa				3	3	0	0	0	0000/00/00	0	2	0
1	13.31	1	55	ant tongue	1	1	1	3	3	x	x	0	0000/00/00	0	2	
1	13.70	1	59	ant tongue	1	1	0	4	4	0	0	0	0000/00/00	0	2	0
1	9.90	0	63	alveolar ridg	1	0	0	1	1	0	0	0	2018/8/22	1	2	0
1	13.11	1	68	ant tongue	1	1	1	1	1	x	x	0	2018/11/6	1	2	
1	13.51	1	69	rmt				4	4	3	1	0	0000/00/00	0	2	1
1	12.98	1	50	mouth floor	1	1	1	4	4	0	0	0	0000/00/00	0	2	0
1	13.21	1	68	ant tongue	1	1	1	1	1	x	x	0	0000/00/00	0	2	
1	13.21	1	52	buccal muco	1	1	1	2	2	0	0	0	0000/00/00	0	2	0
1	7.02	0	40	buccal mucosa				4	4	2	0	0	2018/6/25	1	3	1
1	12.89	1	51	alveolar ridg	1	1	1	4	4	0	0	0	0000/00/00	0	2	0
1	13.31	1	52	ant tongue	1	1	1	2	2	0	0	0	2018/7/16	1	2	0
1	13.21	1	71	buccal muco	1	1	1	1	1	x	x	0	0000/00/00	0	1	
1	13.93	1	76	buccal muco	1	1	1	2	2	x	x	0	0000/00/00	0	2	
1	12.69	1	51	buccal muco	1	0	0	4	4	2	0	0	2019/3/23	1	2	1
1	12.69	1	70	buccal mucosa				2	2	x	x	0	0000/00/00	0	2	
1	12.72	1	51	buccal muco	1	1	1	4	4	3	1	0	0000/00/00	0	2	1
1	14.36	1	41	buccal muco	1	1	0	2	2	0	0	0	0000/00/00	0	2	0
1	13.15	1	62	buccal muco	1	1	1	1	1	0	0	0	0000/00/00	0	2	0
1	12.92	1	53	rmt				4	4	0	0	0	0000/00/00	0	2	0
1	12.52	1	69	alveolar ridg	1	1	1	2	2	0	0	0	0000/00/00	0	2	0
1	6.36	0	66	alveolar ridg	1	1	1	4	4	3	1	0	0000/00/00	0	2	1
1	12.43	1	57	alveolar ridg	1	1	1	4	4	0	0	0	0000/00/00	0	2	0
1	12.52	1	56	hard palate				4	4	x	x	0	2018/12/17	1	2	
1	13.11	1	48	ant tongue	1	1	1	3	1	1	0	0	0000/00/00	0	2	1
1	12.98	1	63	buccal muco	1	1	0	3	3	0	0	0	0000/00/00	0	2	0
1	8.89	0	46	ant tongue	1	1	1	2	2	x	x	0	0000/00/00	0	2	
1	12.75	1	45	buccal mucosa				2	2	0	0	1	0000/00/00	0	3	0
1	12.33	1	68	buccal mucosa				1	1	0	0	0	0000/00/00	0	1	0
1	10.95	0	47	buccal mucosa				4	4	0	0	0	0000/00/00	0	2	0
1	12.20	1	47	alveolar ridg	1	1	1	4	4	0	0	0	0000/00/00	0	2	0
1	12.20	1	65	ant tongue	1	1	1	1	1	x	x	0	2019/5/30	1	2	
1	12.10	1	76	ant tongue	0	0	0	1	1	0	0	0	0000/00/00	0	2	0
1	11.87	1	55	buccal mucosa				2	2	0	0	0	0000/00/00	0	2	0
1	11.77	1	47	buccal mucosa				4	2	2	0	0	0000/00/00	0	2	1
1	11.84	1	59	ant tongue	1	1	1	1	1	0	0	0	0000/00/00	0	2	0
1	11.77	1	58	buccal mucosa				2	2	0	0	0	0000/00/00	0	2	0
1	11.87	1	65	ant tongue	1	1	1	1	1	x	x	0	0000/00/00	0	2	
1	11.64	1	61	rmt	1	1	1	4	4	0	0	0	0000/00/00	0	2	0
1	11.54	1	70	ant tongue	1	1	1	2	2	0	0	0	2019/2/21	1	2	0
1	11.51	1	69	alveolar ridg	0	1	1	4	4	0	0	0	0000/00/00	0	2	0
1	11.57	1	72	buccal mucosa				4	4	0	0	0	0000/00/00	0	1	0
1	11.61	1	79	ant tongue	1	1	1	1	1	x	x	0	0000/00/00	0	2	
1	11.51	1	58	buccal mucosa				2	2	0	0	0	0000/00/00	0	1	0
1	11.48	1	58	hard palate	1	1	1	1	1	x	x	0	2018/12/21	1	2	
1	11.34	1	38	ant tongue	1	0	0	2	2	0	0	0	2019/5/24	1	2	0
1	11.61	1	71	ant tongue	0	0	0	3	3	0	0	0	0000/00/00	0	2	0
1	11.31	1	63	ant tongue	0	0	0	3	3	0	0	0	0000/00/00	0	3	0
1	11.28	1	64	buccal mucosa				4	2	3	1	0	0000/00/00	0	2	1
1	12.23	1	62	ant tongue	1	1	0	2	2	0	0	0	0000/00/00	0	2	0
1	11.31	1	68	buccal mucosa				4	2	3	1	0	0000/00/00	0	2	1
1	10.95	1	73	buccal mucosa				1	1	x	x	1	0000/00/00	0	1	
1	10.95	1	41	ant tongue				4	4	3	1	0	2018/11/24	1	2	1
1	4.66	0	58	mouth floor	1	1	0	4	4	0	0	0	0000/00/00	0	2	0
1	10.62	1	79	buccal muco	1	1	1	1	1	x	x	0	0000/00/00	0	2	
1	10.66	1	58	buccal mucosa				4	4	0	0	0	0000/00/00	0	2	0
1	10.26	1	54	ant tongue				3	3	0	0	0	0000/00/00	0	2	0
1	10.72	1	89	ant tongue				2	2	x	x	0	0000/00/00	0	2	
1	10.03	1	60	buccal mucosa				2	2	0	0	0	2018/11/21	1	2	0
1	10.16	1	36	alveolar ridge				4	4	0	0	0	0000/00/00	0	2	0

1	3.11	0	58	rmt				4	4	3	1	1	0000/00/00	0	2	1
1	10.16	1	47	alveolar ridge				1	1	x	x	0	0000/00/00	0	2	
1	9.90	1	64	ant tongue				1	2	0	0	0	0000/00/00	0	1	0
1	10.00	1	40	buccal mucosa				4	4	3	1	0	0000/00/00	0	2	1
1	9.54	0	67	ant tongue				4	4	3	1	0	2019/5/16	1	2	1
1	9.54	1	45	buccal mucosa				2	2	0	0	0	0000/00/00	0	2	0
1	87.34	1	76	alveolar ridge	1	1	1	2	1	x	x	0	0000/00/00	0	2	
1	9.67	1	73	buccal mucosa				1	1	x	x	0	0000/00/00	0	2	
1	9.54	1	70	alveolar ridge				4	4	2	0	0	0000/00/00	0	2	1
1	7.48	0	61	ant tongue				3	2	1	0	0	2019/3/26	1	2	1
1	9.44	1	54	ant tongue				1	1	x	x	0	0000/00/00	0	2	
1	9.44	1	78	alveolar ridge				1	1	x	x	0	0000/00/00	0	2	
1	9.44	1	51	rmt				4	4	0	0	1	2018/12/31	1	2	0
1	9.48	1	58	alveolar ridge				2	2	0	0	0	0000/00/00	0	1	0
1	9.28	1	65	alveolar ridge				2	2	0	0	0	0000/00/00	0	2	0
1	9.21	1	57	buccal mucosa				2	2	0	0	0	0000/00/00	0	2	0
1	9.18	1	44	rmt				1	1	x	x	0	0000/00/00	0	2	
1	9.21	1	58	alveolar ridge				1	1	x	x	0	0000/00/00	0	1	
1	8.98	1	67	lip				2	2	0	0	0	0000/00/00	0	2	0
1	8.95	1	65	ant tongue				4	2	2	1	0	0000/00/00	0	2	1
1	8.98	1	76	buccal mucosa				1	1	x	x	0	0000/00/00	0	2	
1	8.98	1	59	ant tongue				4	4	0	0	0	0000/00/00	0	2	0
1	8.89	1	56	ant tongue				4	2	2	0	0	0000/00/00	0	2	1
1	8.85	1	60	alveolar ridge				4	4	0	0	0	2018/12/20	1	2	0
1	8.85	1	84	alveolar ridge				2	2	0	0	0	0000/00/00	0	1	0
1	8.89	1	62	lip				4	4	x	x	0	2018/11/30	1	2	
1	9.08	1	58	ant tongue				2	2	0	0	0	0000/00/00	0	2	0
1	0.79	0	72	alveolar ridge				4	4	0	0	0	0000/00/00	0	2	0
1	8.66	1	40	ant tongue				1	1	0	0	0	0000/00/00	0	2	0
1	9.02	1	41	buccal mucosa				4	4	0	0	0	0000/00/00	0	2	0
1	9.08	0	50	buccal mucosa				4	4	0	0	0	2019/2/25	1	2	0
1	9.18	1	48	ant tongue				3	3	0	0	0	0000/00/00	0	2	0
1	8.39	1	70	buccal mucosa				1	1	x	x	0	0000/00/00	0	2	
1	3.02	0	66	ant tongue				4	2	3	1	0	2018/12/4	1	2	1
1	8.36	1	71	ant tongue				2	2	0	0	0	0000/00/00	0	2	0
1	8.30	1	64	ant tongue				4	4	0	0	0	0000/00/00	0	2	0
1	8.36	1	57	rmt				1	1	x	x	0	0000/00/00	0	2	
1	8.30	1	69	alveolar ridge				4	4	x	x	0	0000/00/00	0	2	
1	8.16	1	62	buccal mucosa				4	4	0	0	0	0000/00/00	0	2	0
1	8.07	1	66	alveolar ridge				4	4	3	1	0	0000/00/00	0	2	1
1	8.16	1	66	buccal mucosa				1	1	0	0	0	0000/00/00	0	2	0
1	7.84	1	57	alveolar ridge	1	1	1	1	2	x	x	0	2019/3/15	1	2	
1	8.16	1	74	ant tongue				1	1	0	0	0	0000/00/00	0	2	0
1	7.64	1	73	ant tongue				2	2	x	x	0	0000/00/00	0	2	
1	7.44	1	49	mouth floor				2	2	0	0	0	0000/00/00	0	2	0
1	7.64	1	60	ant tongue				3	1	1	0	0	0000/00/00	0	2	1
1	7.48	1	56	buccal mucosa				1	1	0	0	0	0000/00/00	0	2	0
1	7.15	1	65	buccal mucosa				1	1	0	0	0	0000/00/00	0	2	0
1	6.98	1	78	buccal mucosa				1	1	x	x	0	0000/00/00	0	2	
1	6.95	1	34	ant tongue				2	2	0	0	0	0000/00/00	0	2	0
1	6.75	1	45	buccal mucosa				4	2	3	1	0	0000/00/00	0	2	1
1	6.75	1	67	ant tongue				4	4	2	1	0	2019/2/9	1	2	1
1	6.66	1	62	buccal mucosa				4	3	3	1	0	0000/00/00	0	2	1
1	6.98	1	57	mouth floor				1	1	0	0	0	0000/00/00	0	2	0
1	6.82	1	69	buccal mucosa				3	3	0	0	0	0000/00/00	0	2	0
1	6.43	1	53	alveolar ridge				1	1	x	x	0	0000/00/00	0	2	
1	6.30	1	67	buccal mucosa				2	2	x	x	0	0000/00/00	0	2	
1	6.43	1	68	ant tongue				1	1	x	x	0	0000/00/00	0	1	
1	6.30	1	56	ant tongue				4	4	0	0	0	0000/00/00	0	2	0
1	2.46	0	69	alveolar ridge				4	4	3	1	0	2019/1/15	1	2	1
1	6.36	1	33	buccal mucosa				4	2	3	1	0	0000/00/00	0	2	1
1	6.49	1	58	alveolar ridge				2	2	0	0	0	0000/00/00	0	2	0
1	6.00	1	68	alveolar ridge				1	1	x	x	0	0000/00/00	0	1	
1	5.90	1	81	lip				1	1	0	0	0	0000/00/00	0	2	0
1	6.52	1	49	buccal mucosa				4	3	3	1	0	2019/6/25	1	2	1
1	5.80	1	74	alveolar ridge				1	1	x	x	0	0000/00/00	0	2	
1	5.74	1	50	ant tongue				3	3	0	0	0	0000/00/00	0	2	0
1	5.80	1	56	alveolar ridge				1	1	x	x	1	0000/00/00	0	2	
1	5.64	1	69	buccal mucosa				4	4	0	0	0	0000/00/00	0	2	0
1	6.30	1	71	mouth floor				1	1	0	0	0	2019/6/14	1	2	0
1	5.61	1	70	buccal mucosa				3	3	0	0	0	0000/00/00	0	2	0
1	5.54	1	69	buccal mucosa				1	1	0	0	0	0000/00/00	0	1	0

1	5.44	1	57	ant tongue				1	1	x	x	0	0000/00/00	0	2	
1	5.54	1	72	buccal mucosa				1	1	x	x	0	0000/00/00	0	2	
1	5.54	1	49	buccal mucosa				3	3	0	0	0	0000/00/00	0	1	0
1	6.30	1	59	alveolar ridge				4	1	2	0	0	0000/00/00	0	2	1
1	5.57	1	45	ant tongue				4	4	0	0	0	0000/00/00	0	2	0
1	5.34	1	55	buccal mucosa				1	1	x	x	0	2019/4/2	1	2	
1	5.38	1	55	ant tongue				3	3	0	0	1	0000/00/00	0	1	0
1	5.38	1	64	alveolar ridge				4	4	0	0	0	0000/00/00	0	1	0
1	5.34	1	49	ant tongue				2	2	0	0	0	0000/00/00	0	2	0
1	5.57	1	57	lip				1	1	0	0	0	0000/00/00	0	2	0
1	2.79	0	74	alveolar ridge				4	4	0	0	0	0000/00/00	0	2	0
1	5.18	1	67	rmt				4	4	0	0	0	0000/00/00	0	1	0
1	5.38	1	55	alveolar ridge				4	4	0	0	0	0000/00/00	0	2	0
1	5.21	1	52	buccal mucosa				1	1	x	x	0	0000/00/00	0	1	
1	5.51	1	48	buccal mucosa				1	1	0	0	0	0000/00/00	0	2	0
1	15.93	1	58	hard palate	1	1	1	1	1	0	0	0	0000/00/00	0	1	0
1	13.87	1	69	alveolar ridg(e)	0	1	1	1	1	x	x	0	0000/00/00	0	2	
