# Supplementary material for: Survival and clinicopathological characteristics of different histological grades of oral cavity squamous cell carcinoma: A single-center retrospective study
Source: PLoS One. 2020 Aug 25;15(8):e0238103. doi: 10.1371/journal.pone.0238103 (PMC7447052; doi:10.1371/journal.pone.0238103)
Supplement: Attachment — Submitted filename: Response to Reviewers.docx [file pone.0238103.s002.docx]

**Response letter**

Tittle: Survival and Clinicopathological Characteristics of Different Histological Grades of Oral Cavity Squamous Cell Carcinoma: A Single-Center Retrospective Study

**General response:**

We sincerely thank the editor and all reviewers for their valuable feedback that we have used to improve the quality of our manuscript. The reviewer comments are laid out below in italicized font and specific concerns have been numbered. Our response is given in normal font and changes/additions to the manuscript are given in ”Track changes”.

**Journal Requirements**

*In the ethics statement in the manuscript and in the online submission form, please provide additional information about the patient records used in your retrospective study, including: a) whether all data were fully anonymized before you accessed them; b) the date range (month and year) during which patients' medical records were accessed; and c) the source of the medical records analyzed in this work (e.g. hospital, institution or medical center name). If patients provided informed written consent to have data from their medical records used in research, please include this information.*

Response:

a) All data were fully anonymized before I accessed them.

b) The date range from January, 2008 to June, 2019 during which patients' medical records were accessed.

c) the source of the medical accessed from Changhua Christian Hospital.

*Thank you for stating the following financial disclosure: [The funders had no role in study design, data collection and analysis, decision to publish, or preparation of the manuscript.]. At this time, please address the following queries:*

Response: The authors received no specific funding for this work.

*We note that you have indicated that data from this study are available upon request. PLOS only allows data to be available upon request if there are legal or ethical restrictions on sharing data publicly. For information on unacceptable data access restrictions, please see http://journals.plos.org/plosone/s/data-availability#loc-unacceptable-data-access-restrictions.*

Response: I have uploaded the minimal anonymized data set necessary to replicate my study findings as either Supporting Information files.

**Point-to-point response:**

*Reviewer #1: The authors present a complete and well organised manuscript that highlights the importance of providing histological grade of oral squamous carcinomas. Although the significance of the findings might be deeply related to the demographical and social characteristics of the cohort (betel chewing for example), the main suggestions about the value of the histological grading in OSCC in this report are still universally valuable and in line with practice with other type of malignancies affecting other organs/systems and that are stratified by histological grade.*

Response: Thank you very much for your positive comments.

*The authors exclude from the cohort cases of carcinomas of the lip not affecting labial mucosa, but Table 5 reports tumors of the lips, which is confusing. Indicating this group as "labial mucosa" sounds more correct and takes away this confusing.*

Response: In this study, we only enrolled those patients with SCC on mucosa of lip. We have excluded those patients with cutaneous squamous cell carcinoma of the vermilion lip from the very first. For better understanding, we have made change on the Table to prevent misunderstanding. Thank you for the comment.

*The authors correctly point that the histological grade might be to a certain degree subjective, and here the grading is performed by only one individual, which should be pointed also in the discussion as one of the challenges with this manuscript.*

Response: Thank you very much for your suggestion. In our center, once malignant tumors diagnosed, the resected specimens should be evaluated by two experienced pathologists. We apologize for our negligence. We have made change for the manuscript and added the drawback you have mentioned into the Discussion. The change can be found on line 91 to 93 and line 337 to 339.

*Finally, the authors use OSCC but also SCC and should keep one form; in page 2 and page 15 "should not be disregarded" might be better expressed as for "should be followed more closely than OSCC in early stages but higher differentiation degree"; in page 12 it should be "poor differentiation" not "poor differentiate"; in page 15 I guess authors mean "diagnostic field" and not "diagnostic filed".*

Response:

1. I have made change accordingly. The change can be found in the revised manuscript.

2. I have made change accordingly. The change can be found on line 42 to 43 and line 298 to 300.

3. I have made change accordingly. The change can be found on line 241 and line 342.

*Reviewer #2: Dear authors, Thank you for the opportunity to review this manuscript in a very interesting topic. I have several concerns about your manuscript pointed bellow. I strongly recommend an English language review. You should considerer to reestructure the manuscript, this atual version is presented in a very confuse way.*

*Abstract - The abstract section is very confuse and do not reflect clearly the content of the study.*

Response:

*- The introduction section is a little poor, the authors should consider to include one or two more paragraphs to state the problem to the readers.*

Response: Thanks for your suggestion. I have added paragraphs into Introduction accordingly. The change can be found on line 57 to 65.

*Patients and Methods - The authors have to add the number and the date of the ethical board protocol aprouvement. (page 3)*

Response: Thanks for your suggestion. I have added IRB number and the approved date accordingly.

*- In my point of view include lip squamous cell carcinoma is a bias in your study. It is well known that, in this location, the neoplasia have a complete different biological behaviour. (page 4)*

Response: In this study, we only enrolled those patients with SCC on mucosa of lip. We have excluded those patients with cutaneous squamous cell carcinoma of the vermilion lip from the very first. For better understanding, we have made change on Table to prevent misunderstanding. Thank you for the comment.

*- The authors have to explain better the histopathological classification, especially how it has been performed, this is the central part of the study. Only one pathologist performed the analysis? How many slides per day? Directly in a microscope? With an image manager program? (page 4) - Has the immunohistochemistry been performed in a regular basis? How many cases? In wich conditions?(page 5)*

Response: Thank you very much for your suggestion. In our center, the resected specimens of malignant tumors should be evaluated by two experienced pathologists. We apologize for our negligence. I have added a paragraph to strengthen Patients and Methods. Thanks again for your valuable suggestion.

*- As mentioned, the inclusion of the lip is a bias in your study. In my point of view, the authors should exclude this region. (page 5)*

Response: In this study, we only enrolled those patients with SCC on mucosa of lip. We have excluded those patients with cutaneous squamous cell carcinoma of the vermilion lip from the very first. For better understanding, we have made change on Table to prevent misunderstanding. Thank you for the comment.

*Results – In general the results were presented in a very confuse way, the authors should rewrite this part of the manuscript. The tables are very difficult to understand and some of them are unnecessary.*

Response: Thanks for your suggestion. For better understanding, I have reconstructed Tables and re-write Result. In Table 3, I have removed the unnecessary parameters including age, gender, and site of tumors because the main concern was I want to compare grade of tumors with the other common prognostic factors. Thanks for your valuable suggestion to increase the quality of our manuscript.

*Discussion*

*– In my point of view the discussion section is a bit confuse and include a restrict number of references, considering this is a classic topic (only 18 references in total).*

*- The authors should rewrite the discussion section according to the recostructured results section in a possible future revised version.*

Response: Thanks for your suggestion. I have indeed found several articles related to the topic during the past years and I have added the articles into my reference. I have re-written the Discussion extensively. Thanks for your valuable suggestion to improve the quality of our manuscript.

*Reviewer #3: Although the finding are significant, the manuscript is not written well. Therefore I advise you to get the help from a language expert. There is a significant amount of literature missing in the reference list.*

Response: I have indeed found several articles related to the topic during the past years and I have added the articles into my reference. I have re-written manuscript extensively and used Enago for language editing. Thanks for your valuable suggestion to improve the quality of our manuscript.

We have revised the manuscript extensively based on the reviewer’s comments. If there are any other modifications we could make, we would like very much to do so and we greatly appreciate your help. We hope that our manuscript will be considered for publication in your journal. Thank you very much.

Sincerely,

Kuo-Yang Tsai

Department of Oral and Maxillofacial Surgery, Changhua Christian Hospital

No. 235, Xuguang Rd, Changhua City, Changhua County 500, Taiwan

Phone: +886 933127916

Email: [72837@cch.org.tw](mailto:72837@cch.org.tw)
