# Supplementary material for: Survival and clinicopathological characteristics of different histological grades of oral cavity squamous cell carcinoma: A single-center retrospective study
Source: PLoS One. 2020 Aug 25;15(8):e0238103. doi: 10.1371/journal.pone.0238103 (PMC7447052; doi:10.1371/journal.pone.0238103)
Supplement: Attachment — Submitted filename: Response to Reviewers.docx [file pone.0238103.s003.docx]

**Response letter**

Tittle: Survival and Clinicopathological Characteristics of Different Histological Grades of Oral Cavity Squamous Cell Carcinoma: A Single-Center Retrospective Study

**General response:**

We sincerely thank the editor and all reviewers for their valuable feedback that we have used to improve the quality of our manuscript. The reviewer comments are laid out below in italicized font and specific concerns have been numbered. Our response is given in normal font and changes/additions to the manuscript are given in ”Track changes”.

**Point-to-point response:**

*Reviewer #1: I commend the authors for having worked with the text and accepting our suggestions, and the presentation of the data and the discussion are more complete and precise. The manuscript has improved on the language side as well, but there are still mistakes in the text (page 8 "...grade 2, years, and grade 3 OSCC..."; page 9 "...most..." instead of "...mostly..."; page 16 "re-wide excision" never heard before, I guess the authors mean "wider re-excision").*

Response: Thank you very much for your positive comments and suggestions. I have revised the manuscript accordingly.

*In page 17 the authors write that "...grading attempts are inexact, subjective, not reproducible." It is also my experience that most squamous carcinomas fall in the definition of moderate grade of differentiation, but the authors are quite sharp in their definition. And just using keratinising vs non keratinising could give the same problem and be potentially subjective. This has been dealt with by other authors who suggested a to-tier grading to improve the concordance of the grading [I can quickly suggest for example Krishnan, Karpagalvi...Patil, J Oral Maxillofac Pathology (2016) and Steigen, Søland...Olsen, J Oral Pathol Med (2020)].*

Response: Thank you very much for your suggestion. I found the conclusion in Steigen et al. was similar to my point of view, they suggested that simpler/uncomplicated scoring protocols will increase the reproducibility. In their study, Intrarater comparison of WHO grading system, after in binary categorization, mean agreement could reach 92.5%. I have added the article into my reference and revised the manuscript in smooth tone. Thanks again for your valuable help.

*Also, in the text the authors now write that two pathologists classified the material and not just one, but the numbers of the groups were the same as far as I can see, and the authors write nothing on how the two pathologists came to an agreement. Did the pathologists agree on all cases? In this case why do the authors write the grading is not reproducible?*

Response: In the present study, we investigated the grade of OSCC in retrospective fashion (based on final pathological report). The pathological report was accomplished by two pathologists within 10 days after surgery had been done. And the intervention of third pathologist was engaged if the first two pathologists could not reach a consensus. In our study we only presented the final agreement of the grade of tumors documented on the pathological report. Grading was achieved by human through microscope and it is inevitable that human made different judgment in those borderline cases. Steigen et al. also reported that simpler/uncomplicated scoring protocols will increase the reproducibility (two options better than three options, and three options better than fine or four options). In our study, we did not verify the reproducibility of OSCC grading, however, it is very hard to achieve 100% the same result when all our cases graded in the other center or other country.

*This point (only one pathologist) and a point previously addressed (grade 3 cases should not be "disregarded") have not been addressed in the abstract, that should be corrected as well. The text is written in good English all in all but the imprecisions deter from the scientific value of the study, real piety since I find the study really useful also in clinical settings, reminding pathologists to provide clinicians with a histological variable that can be evaluated on routinely provided HE slides.*

Response:

Thank you very much for your suggestion. I have revised the Abstract accordingly. In the present study, I would like to suggest clinicians do not overlook the importance of the grade of OSCC, in 2018 AJCC Head and Neck guideline, tumor invade depth was added in TNM staging. In somedays, histological grade of OSCC or a new grading system maybe added into TNM stage.

*Reviewer #2: The main questions addressed in the first round of revision were not properly answered. Including the lip OSCC is one of the major concern in the manuscript.*

Response: Thanks for your comments. In AJCC 2018 head and neck cancer guideline, oral cavity had included the mucosal lip. In Southeast Asia, the main factors contributing to mucosal lip SCC are betel nuts chewing, smoking and alcohol consumption and these factors are the same in the oral cavity SCC. Meanwhile, I have reviewed many researches for OSCC, and found these article also included mucosal lip SCC into their study (I take two study for example: Brinkman D, Callanan D, O'Shea R. Impact of 3 mm margin on risk of recurrence and survival in oral cancer. Oral Oncol. 2020;110:104883. doi:10.1016/j.oraloncology.2020.104883 and Mochizuki Y, Harada H, Ikuta M, et al. Clinical characteristics of multiple primary carcinomas of the oral cavity. Oral Oncol. 2015;51(2):182-189. doi:10.1016/j.oraloncology.2014.11.013 ). If you insist excluding the entity of the mucosal lip in the present study, we would like very much to do so and we greatly appreciate your help.

In the first round of revision, we have revised the manuscript extensively based on your suggestions. In the present study, we investigated the grade of OSCC in retrospective fashion (based on final pathological report). The pathological report was accomplished by two pathologists within 10 days after surgery had been done. And the intervention of third pathologist was engaged if the first two pathologists could not reach a consensus. Immunohistochemical confirmation for cytokeratin was performed to detect poorly differentiated OSCC because features of squamous differentiation were minimal or absent. In our study we only presented the final agreement of the grade of tumors documented on the pathological report. In the present study, I would like to suggest clinicians do not overlook the importance of the grade of OSCC, in 2018 AJCC Head and Neck guideline, tumor invade depth was added in TNM staging. In somedays, histological grade of OSCC or a new grading system maybe added into TNM stage. If there are any other modifications we could make, we would like very much to do so.

We have revised the manuscript extensively based on the reviewer’s comments. If there are any other modifications we could make, we would like very much to do so and we greatly appreciate your help. We hope that our manuscript will be considered for publication in your journal. Thank you very much.

Sincerely,

Kuo-Yang Tsai

Department of Oral and Maxillofacial Surgery, Changhua Christian Hospital

No. 235, Xuguang Rd, Changhua City, Changhua County 500, Taiwan

Phone: +886 933127916

Email: [72837@cch.org.tw](mailto:72837@cch.org.tw)
